# DelSIEVE: cell phylogeny modeling of single nucleotide variants and deletions from single-cell DNA sequencing data

**DOI:** 10.1186/s13059-025-03738-9

**Published:** 2025-08-25

**Authors:** Senbai Kang, Nico Borgsmüller, Monica Valecha, Magda Markowska, Jack Kuipers, Niko Beerenwinkel, David Posada, Ewa Szczurek

**Affiliations:** 1 Faculty of Mathematics, Informatics and Mechanics, University of Warsaw, Warsaw, Poland; 2 Department of Biosystems Science and Engineering, ETH Zurich, Basel, 4058 Switzerland; 3 SIB Swiss Institute of Bioinformatics, Basel, 4058 Switzerland; 4 CINBIO, Universidade de Vigo, Vigo, 36310 Spain; 5 Galicia Sur Health Research Institute (IIS Galicia Sur), SERGAS-UVIGO, Vigo, Spain; 6 Medical University of Warsaw, Postgraduate School of Molecular Medicine, Warsaw, Poland; 7 Department of Biochemistry, Genetics, and Immunology, Universidade de Vigo, Vigo, 36310 Spain; 8 Institute of AI for Health, Helmholtz Zentrum München, German Research Center for Environmental Health, Neuherberg, Germany

**Keywords:** Single-cell DNA sequencing, Statistical phylogenetic models, Cell phylogeny reconstruction, Single nucleotide variants, Deletions, Acquisition bias correction, Colorectal cancer, Triple negative breast cancer

## Abstract

With rapid advancements in single-cell DNA sequencing (scDNA-seq), various computational methods have been developed to study evolution and call variants on single-cell level. However, modeling deletions remains challenging because they affect total coverage in ways that are difficult to distinguish from technical artifacts. We present DelSIEVE, a statistical method that infers cell phylogeny and single-nucleotide variants, accounting for deletions, from scDNA-seq data. DelSIEVE distinguishes deletions from mutations and artifacts, detecting more evolutionary events than previous methods. Simulations show high performance, and application to cancer samples reveals varying amounts of deletions and double mutants in different tumors.

**Supplementary information:**

The online version contains supplementary material available at 10.1186/s13059-025-03738-9.

## Background

Cancer is a genetic disease driven by the accumulation of somatic mutations, resulting in highly heterogeneous cell populations [1–5]. The most common types of somatic mutations are single nucleotide variants (SNVs), followed by *deletions*, including point deletions, small deletions, and copy number aberrations. These events together can result in the activation of oncogenes and the inactivation of tumor suppressor genes, thus promoting tumor proliferation [2, 3, 5–8].

Single-cell DNA sequencing (scDNA-seq) technologies exhibit great potential for the analysis of intratumor genetic heterogeneity at the highest resolution of individual cells [9–12]. However, these technologies typically suffer from a low signal-to-noise ratio. Most rely on whole-genome amplification (WGA) before sequencing [12–16], with non-scWGA methods either not providing enough coverage to call SNVs [17] or only sequencing a panel of genes instead of whole genome or exome [18]. The WGA step introduces several biases in the sequencing data, including an uneven coverage of the genome, amplification errors, and allelic bias, where one of the paternal or maternal alleles is over- or underrepresented. Importantly, allelic bias can sometimes result in allelic (ADO) or locus dropout (LDO), where one or both alleles fail to be amplified [12–14].

Several different methods for calling SNVs from scDNA-seq data have been proposed. For instance, Monovar [19] employs consensus filtering based on the data from multiple cells, while other methods [20–22] leverage phase information from germline single nucleotide polymorphisms. The called SNVs are typically used for the reconstruction of the cell phylogeny [23–30]. As the cell phylogeny can be informative for SNV calling, SCIPhI [31] and our more recent model SIEVE [32] jointly infer the cell phylogeny and call SNVs.

However, the majority of methods that model the cell phylogeny or call SNVs from scDNA-seq data do not account for deletions and consider only diploid genotypes during tumor evolution. The difficulty of accurate modeling of SNVs in the presence of deletions arises because the effects of deletions, back mutations, double mutants, allelic imbalance, and dropouts on sequencing data are often hard to distinguish. For example, several events might be the cause if only reads supporting the alternative nucleotide are observed. Assuming that one of the alleles has such alternative nucleotide, the other allele could either be deleted during evolution, be dropped out during amplification, or be mutated to exactly the same alternative nucleotide.

To address these ambiguities, methods such as SCARLET [33] or SCIPhIN [34] leveraged the idea that deletions occur along the cell phylogeny and thus groups of related cells should share the same deletions. However, these methods are unable to identify important evolutionary events such as double mutants (mutations affecting both alleles at a variant site) and do not fully exploit the information conveyed by sequencing coverage.

We reasoned that combining the information encoded in the raw read counts, especially the signal in sequencing coverage, and the phylogenetic relations among cells should result in more accurate inference of phylogenetic trees and variants in the presence of deletions. Indeed, despite the inherent noise in scDNA-seq data, it is expected that the sequencing coverage is proportional to the number of sequenced alleles and should provide information on the loss of alleles. On the other hand, the cell phylogeny should help to tell if the loss occurs during evolution or due to technical artifacts.

Here we introduce DelSIEVE (deletions enabled SIngle-cell EVolution Explorer), a statistical phylogenetic model that leverages both the signal from cell phylogeny and the coverage information, and explicitly accounts for the effect that deletions have on mutated sites. DelSIEVE can call seven different genotypes that not only include single or double mutants, but also single or double deletions, and is able to discern those from technical events such as ADO or LDO. Thanks to this increased expressive power, DelSIEVE is able to discern 28 types of genotype transitions, associated with 17 types of mutation events, many more than any existing method.

## Results

### Overview of the DelSIEVE model

**Fig. 1:**
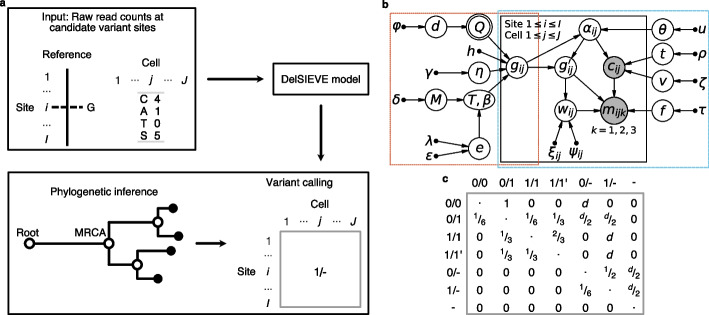
Overview of the DelSIEVE model. **a** Analysis workflow of DelSIEVE with an example of input data. At candidate variate site $i \in \{ 1, \dots , I \}$, the reference nucleotide is G. For cell $j \in \{ 1, \dots , J \}$ at site *i*, observed are the sequencing depth of 5 (marked by *S*) as well as read counts for nucleotide *C* being 4 and *A* being 1. DelSIEVE first infers from the input data the cell phylogeny, based on which the genotype state of each node in the tree is then determined through maximum likelihood estimation. For instance, 1/- is inferred as the genotype state of cell *j* at site *i*. **b** Probabilistic graphical model of DelSIEVE. The orange frame shows the part corresponding to the statistical phylogenetic model, and the blue frame encloses the part corresponding to the model of raw read counts. Shaded circular nodes represent observed variables, while unshaded circular nodes represent hidden random variables. Nodes with double circles are deterministic random variables, meaning that they are fixed once the values of their parent nodes are determined. Small black dots correspond to fixed hyper parameters. Arrows denote local conditional probability distributions of child nodes given parent nodes. **c** Instantaneous transition rate matrix of the statistical phylogenetic model. The hidden random variable *d* is the deletion rate, measured relatively to the mutation rate. The elements in the diagonal of the matrix are denoted by dots and have negative values opposite to the sum of the other entries in the same row, ensuring that the sum of each row equals zero

DelSIEVE takes as input raw read counts for all four nucleotides for each cell $j \in \{ 1, \dots , J \}$ at each candidate site $i \in \{ 1, \dots , I \}$ in the form of the read counts of three alternative nucleotides with values in descending order, together with the total sequencing coverage (Fig. 1a).

From that input data, the model first infers a tree phylogeny, which incorporates a trunk between the root (a normal cell) and the most recent common ancestor (MRCA) of the sampled tumor cells. DelSIEVE operates in a genotype state space that accounts both for SNVs and deletions of candidate variant sites. Specifically, apart from genotypes that were previously modeled by SIEVE: 0/0 (*wildtype*), 0/1 (*single mutants*), 1/1 (*double mutants*, where the two alternative nucleotides are the same), and $1/1^{\prime }$ (*double mutants*, where the two alternative nucleotides are different), DelSIEVE additionally considers 0/- (*reference-remaining single deletion*), 1/- (*alternative-remaining single deletion*), and - (*double deletions*). Here, $0, 1, 1^{\prime }$ and - represent the reference nucleotide, an alternative nucleotide, a second alternative nucleotide different from that denoted by 1, and deletions, respectively. The genotype state of each node in the tree is inferred using maximum likelihood estimation. As an effect, DelSIEVE is able to discern 28 types of genotype transitions, which we categorize into 17 different mutation events (eight more than SIEVE; see Mutation event classification section and Table 3). These genotype transitions include 12 that were already previously modeled by SIEVE, and are complemented by 16 transition events associated to deletions.

The power of DelSIEVE lies in its probabilistic graphical model, where the hidden variable describing the genotype for site *i* in cell *j*, denoted $g_{ij}$, is used as the bridge between the statistical phylogenetic model and the model of raw read counts (Fig. 1b). The model accounts for the possible mutations using a deletion-aware instantaneous transition rate matrix (Methods; Fig. 1c). DelSIEVE employs a Dirichlet-Multinomial distribution to model the raw read counts for all nucleotides, and models the sequencing coverage using a negative binomial distribution, dependent on the number of alleles which can change due to deletions (see Methods for a detailed description).

### DelSIEVE accurately calls deletions

We first used simulated data to benchmark one of DelSIEVE’s advantages, namely calling deletions (see Simulation design section in Additional file 1: Supplementary notes). To our knowledge, DelSIEVE is the only method that can differentiate alternative-remaining single deletion (genotype 1/-), reference-remaining single deletion (0/-), and double deletions (-), and thus it was not compared to any other method for these tasks.

**Fig. 2:**
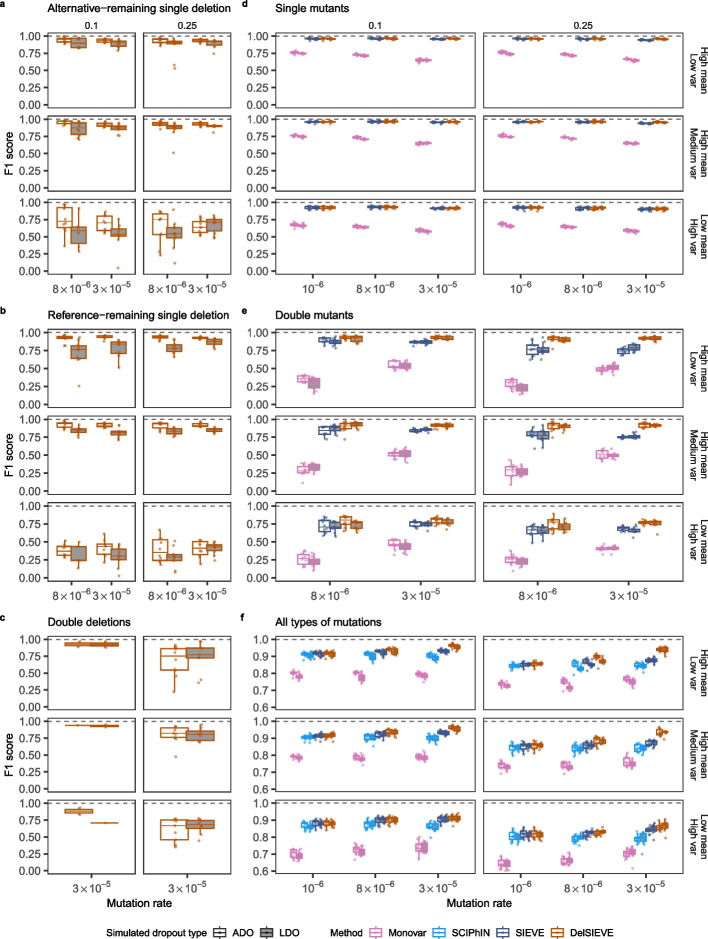
F1 score for the benchmark of the DelSIEVE model. Varying are the mutation rate (the horizontal axis), the relative deletion rate (the vertical strip), the coverage quality (the horizontal strip), and the simulated dropout type (the shaded or blank boxes). Each simulation is repeated $n = 10$ times, with each repetition denoted by colored dots. The gray dashed lines represent the optimal values of each metric. Box plots comprise medians, boxes covering the interquartile range (IQR), and whiskers extending to 1.5 times the IQR below and above the box. Data points were removed if the proportion of simulated ground truth was less than 0.1%. Both DelSIEVE and SIEVE were configured to match the dropout mode (ADO or LDO) employed during the simulation process. Box plots of the F1 score for calling alternative-remaining single deletion (**a**), reference-remaining single deletion (**b**), double deletions (**c**), single mutants (**d**), and double mutants (**e**). The results in **c** when mutation rate was $8 \times 10^{-6}$ were omitted as very few double deletions were generated (less than 0.2%; see Simulation design section in Additional file 1: Supplementary notes). **f** Box plots of the F1 score for calling all types of mutations considered in **a**–**e**

For calling alternative- and reference-remaining single deletions, DelSIEVE achieved F1 scores with medians $\ge 0.87$ and $\ge 0.76$, respectively, when the data was of medium or high coverage quality (with high mean and low or medium variance of coverage; Fig. 2a, b). For calling these two genotypes, the corresponding recall of DelSIEVE has medians $\ge 0.72$ and $\ge 0.62$ (Additional file 2: Fig. S1a, c), the precision medians $\ge 0.96$ and $\ge 0.97$ (Additional file 2: Fig. S1b, d), and the false positive rate (FPR) medians $\approx 0$ (Additional file 2: Fig. S2a, b). These results show that DelSIEVE can correctly and reliably identify most of the alternative- and reference-remaining single deletions.

When the data was of low coverage quality (low mean and high variance of coverage), the F1 score medians of DelSIEVE dropped to $\ge 0.55$ and $\ge 0.29$ for calling alternative- and reference-remaining single deletions, respectively (Fig. 2a, b). The low quality of the data affected more the calling of the reference-remaining single deletion (Additional file 2: Fig. S1a–d), which was expected as such low coverage provided little information for this task. Furthermore, the FPR of DelSIEVE was still $\approx 0$ for the low quality data.

When calling double deletions, DelSIEVE obtained high F1 scores medians $\ge 0.75$ (Fig. 2c). Its performance decreased as the deletion rate increased or the coverage quality of the data decreased (Fig. 2c, Additional file 2: Fig. S1e, f), but the FPR was kept at a negligible level ($\approx 0$; see Additional file 2: Fig. S2c).

We observed that the performance of DelSIEVE only slightly decreased in the presence of LDO, in comparison to the results obtained when it was applied to data simulated under the ADO mode. Given that DelSIEVE explicitly models the sequencing coverage, it was anticipated that data simulated under the LDO mode would introduce additional uncertainties to the inference.

### DelSIEVE reliably identifies mutations in the presence of deletions

We next assessed DelSIEVE’s performance in calling single and double mutants against Monovar and SIEVE (Fig. 2d, e, Additional file 2: Figs. S3, S4).

Regarding calling single mutants, DelSIEVE and SIEVE performed comparatively well (minimum median F1 score 0.9) and outperformed Monovar (minimum median F1 score 0.58; see Fig. 2d). This advantage can be due to the fact that in contrast to Monovar, DelSIEVE and SIEVE consider the cell phylogeny during variant calling. As the mutation rate increased, the recall of both DelSIEVE and SIEVE slightly increased (Additional file 2: Fig. S3a), while the precision slightly decreased (Additional file 2: Fig. S3b), resulting in relatively constant F1 scores. In contrast, Monovar experienced a decrease in both recall and precision as the mutation rate increased (Additional file 2: Fig. S3a, b). Moreover, DelSIEVE and SIEVE had comparable recall (Additional file 2: Fig. S3a), while DelSIEVE showed higher precision (Additional file 2: Fig. S3b) and lower FPR (Additional file 2: Fig. S4a) than SIEVE, especially when the mutation rate was high ($3 \times 10^{-5}$). We speculate that this might be because SIEVE has to interpret the evident signal of deletions as ADO or LDO events occurring in addition to mutations.

Additionally, as the mutation rate increased, the FPR of all methods also increased (Additional file 2: Fig. S4a). It was noteworthy that, when the mutation rate was high ($\ge 3 \times 10^{-5}$), DelSIEVE and SIEVE had slightly higher FPR than Monovar for calling single mutants (Additional file 2: Fig. S4a). However, this loss was negligible compared to SIEVE and DelSIEVE’s advantage over Monovar when considering precision, recall, and F1 score.

In the task of calling double mutants, Monovar obtained minimum median F1 scores of 0.21, while SIEVE and DelSIEVE exhibited much better performance with minimum median F1 scores 0.65 and 0.93, respectively (Fig. 2e). More specifically, DelSIEVE and SIEVE had a comparable recall (Additional file 2: Fig. S3c), but the former reached higher precision (minimum medians 0.75 and 0.61, respectively; see Additional file 2: Fig. S3d). Again, this discrepancy in performance could be due to SIEVE’s inclination to explaining deletions as dropout events occurring on top of double mutants.

DelSIEVE also had the lowest FPR ($\approx 0$) (Additional file 2: Fig. S4b). These findings highlighted the superior accuracy of DelSIEVE in identifying double mutants in the presence of deletions. On top of that, the slight advantage of Monovar over methods incorporating phylogeny for calling single mutants was not observed for calling double mutants. In contrast, Monovar had a significantly elevated FPR in this task compared to all other methods.

### DelSIEVE outperforms alternative models in variant calling, regardless of the variant type

To compare to one more predecessor model, SCIPhIN, which does not distinguish among single and double mutants, as well as alternative-remaining single deletion, reference-remaining single deletion, and double deletions, we considered all genotypes other than wildtype as general “mutations” and computed the related performance metrics (see Variant calling and phylogenetic accuracy section in Additional file 1: Supplementary notes).

Overall, Monovar was outperformed by the other three methods (Fig. 2f, Additional file 2: Fig. S5a–c), which had similar performance when the mutation rate was low ($10^{-6}$). As the mutation rate increased, DelSIEVE performed better than SIEVE and SCIPhIN (Fig. 2f). Specifically, DelSIEVE had higher recall compared to SIEVE and SCIPhIN (Additional file 2: Fig. S5a), with similar precision and FPR (Additional file 2: Fig. S5b, c). With the increase of the relative deletion rate and the decrease of the coverage quality, the performance of all methods slightly dropped. The dropout mode under which the data was simulated seemed to have an insignificant effect on all methods, except for the precision and FPR of Monovar, which were worse under the LDO mode (Additional file 2: Fig. S5b, c).

### DelSIEVE can identify ADO and LDO

**Fig. 3:**
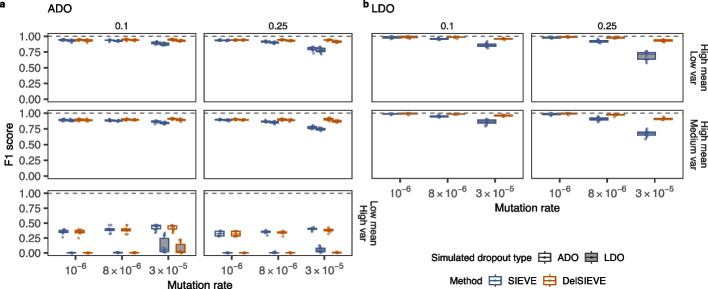
F1 score for the benchmark of calling ADO and LDO. Varying are the mutation rate (the horizontal axis), the relative deletion rate (the vertical strip), the coverage quality (the horizontal strip), and the simulated ADO type (the shaded or blank boxes). Each simulation is repeated $n = 10$ times, with each repetition denoted by colored dots. The gray dashed lines represent the optimal values of each metric. Box plots comprise medians, boxes covering the interquartile range (IQR), and whiskers extending to 1.5 times the IQR below and above the box. Both DelSIEVE and SIEVE were configured to match the dropout mode (ADO or LDO) employed during the simulation process. Box plots of the F1 score for calling ADO (**a**) and LDO (**b**). The F1 scores were unavailable in **b** when data was of low coverage quality due to unavailable precision. The results of calling LDO for data simulated with ADO are not available in **b**, as both models were configured for the same dropout mode as used in the simulated data and were not able to call LDO in this case

We then evaluated DelSIEVE’s performance in calling ADO and LDO against SIEVE (Fig. 3, Additional file 2: Figs. S6, S7), which are the only two methods that can infer these events. Though unsupported originally, we implemented the LDO mode in SIEVE for this comparison (see Configurations of methods section in Additional file 1: Supplementary notes).

ADO calling was affected by the coverage quality. When the data was of medium or high coverage quality, DelSIEVE reached a minimum median F1 score of 0.9, higher than SIEVE (0.77; see Fig. 3a). The performance of DelSIEVE remained consistent regardless of changes in the mutation and deletion rates, in contrast to SIEVE. This was anticipated because higher mutation or deletion rates resulted in an increased number of deletions being generated. DelSIEVE was capable of differentiating deletions from ADOs, while SIEVE wrongly accounted for deletions as ADOs occurring on top of single or double mutants. This behavior reduced SIEVE’s recall and precision, and increased FPR (Additional file 2: Fig. S6a, b, Additional file 2: Fig. S7a), as when calling single and double mutants (see DelSIEVE reliably identifies mutations in the presence of deletions section). The performance of DelSIEVE and SIEVE in calling ADO declined when the data had low coverage quality (Fig. 3a, Additional file 2: Fig. S6a, b, Additional file 2: Fig. S7a). This decrease in performance was further exacerbated when the data was simulated under the LDO mode.

When calling LDOs from data of medium or high coverage quality, DelSIEVE showed a minimum median F1 score of 0.91, higher than SIEVE did (0.68; see Fig. 3b). Specifically, DelSIEVE and SIEVE were comparable in terms of recall (Additional file 2: Fig. S6c), but DelSIEVE had a higher precision and lower FPR as the mutation and deletion rates increased (Additional file 2: Fig. S6d, Additional file 2: Fig. S7b). However, when the data was of low coverage quality, both methods reported no LDO, resulting in zero recall and FPR as well as not available precision and F1 score values.

### DelSIEVE estimates accurate cell phylogenies

We further benchmarked DelSIEVE’s performance in reconstructing the cell phylogeny against SiFit, SCIPhIN, and SIEVE (Additional file 2: Fig. S8). To measure phylogenetic accuracy, we used the branch score (BS) distance, which considers both tree topology and branch lengths and the normalized Robinson-Foulds (RF) distance, which only considers the tree topology (see Variant calling and phylogenetic accuracy section in Additional file 1: Supplementary notes). The results of SCIPhIN were excluded in the computation of the BS score as it does not estimate branch lengths.

DelSIEVE and SIEVE outperformed SiFit when branch lengths were considered, showing the advantage of correcting the acquisition bias (Additional file 2: Fig. S8a). Moreover, all methods tended to overestimate branch lengths when the mutation rate was higher ($\ge 8 \times 10^{-6}$).

The performance of DelSIEVE and SIEVE in topology reconstruction was similar (maximum median normalized RF distance 0.29 and 0.28, respectively), and better compared to SiFit (maximum median normalized RF distance 0.37) and SCIPhIN (0.33; see Additional file 2: Fig. S8b), especially when the mutation rate increased. DelSIEVE and SIEVE were robust to variations in mutation rates in comparison to SiFit and SCIPhIN, while the performance of all methods declined as the coverage quality decreased. The high performance of DelSIEVE in variant calling and phylogenetic reconstruction is likely due to the benefit of sharing information between these two tasks.

### The dropout mode configuration of DelSIEVE has negligible effect on performance

The previous results were obtained with DelSIEVE configured to match the dropout mode (ADO or LDO) employed during the simulation process. To investigate the effects of model misspecification, we further ran DelSIEVE (and, for completeness, where possible, also SIEVE) under a dropout mode different from that used to simulate the data (see Configurations of methods section in Additional file 1: Supplementary notes).

The configuration of the dropout mode, regardless of that used in the simulated data, did not significantly affect DelSIEVE’s calling of deletions (Additional file 2: Fig. S9a–c), or DelSIEVE’s and SIEVE’s calling of single and double mutants (Additional file 2: Fig. S9d, e). We also observed that for simulated data of high coverage quality under the ADO mode, the dropout mode of DelSIEVE and SIEVE did not affect ADO calling (Additional file 2: Fig. S10). However, for data of the high coverage quality but under the LDO mode, it was favored to run those methods under the same dropout mode. On the contrary, when the data was of low coverage quality, it was favorable to run both methods under the ADO mode, regardless of that used to generate the data. Finally, the dropout configuration did not affect the phylogeny reconstruction of SIEVE and DelSIEVE (Additional file 2: Fig. S11a, b), except for the high mutation rate and coverage quality for the BS score of DelSIEVE, where running under ADO mode slightly increased the estimated branch lengths (Additional file 2: Fig. S11a). Since the real dataset analyzed in this work resembles the low coverage quality data, running DelSIEVE under ADO mode should have a negligible effect on the tree reconstruction.

Given that the LDO versus ADO mode configuration affects the model’s performance only slightly and given that LDOs are relatively rare compared to ADOs, we ran DelSIEVE in ADO mode for the analysis of the real datasets discussed below.

### DelSIEVE is robust to occurrence of doublets and moderate copy number aberrations (CNAs)

DelSIEVE accounts for deletions in variant sites, and as such it considers copy number 2, 1, and 0. However, occurrences of CNAs do not only reduce the copy number for some sites through deletions, but also increase it via amplifications. CNAs, acting on entire genomic regions that potentially span several variant sites at once, violate the independent site assumption. In addition, doublet cells may occur during library preparation, wherein genomic material from two distinct cells is captured as if originating from a single cell. Thus, both CNAs and doublet cells may introduce substantial noise and ambiguity into sequencing data, obscuring phylogenetic inference and variant calling.

To evaluate the effect of CNAs and doublet cells on DelSIEVE’s performance, we simulated CNAs by sampling the copy number *n* from $\{ n \in \mathbb {Z} \mid 0 \le n \le 10,\; n \ne 2 \}$ for either $\frac{1}{3}$ or for $\frac{2}{3}$ of all sites (see Simulation design section in Additional file 1: Supplementary notes). Both data with and without CNA-affected sites were used as input to DelSIEVE. We also simulated doublets by having either 2% or 10% of cells mixed with other cells (see Simulation design section in Additional file 1: Supplementary notes). To create the most challenging scenario, we simulated datasets incorporating both extensive CNAs affecting $\frac{2}{3}$ of all sites and doublets involving 10% of cells.

Moderate abundance of CNAs had negligible effect on calling all variants (Additional file 2: Figs. S12–S15). The extensive amount of CNAs in $\frac{2}{3}$ of the sites affected mostly the deletion-related genotype calling, namely the alternative- and reference-remaining genotypes (Additional file 2: Figs. S12, S13). Occurrence of doublet cells did not affect variant calling performance. Consequently, the combination of extensive CNAs and doublets had similar effects as extensive CNAs only (Additional file 2: Figs. S12–S15). Calling single ADOs was robust to moderate CNAs and occurrence of doublets and was only impaired by large abundance of CNAs (Additional file 2: Fig. S16).

As for the inference of cell phylogeny, the presence of CNAs had little impact on the inferred branch lengths and tree structure (Additional file 2: Fig. S17). The only exception was that when CNAs appeared in $\frac{2}{3}$ of all sites, DelSIEVE tended to infer longer branch lengths than they truly were. The reason might be that for the excessive number of deletions introduced by CNAs, DelSIEVE had to explain them as individual evolutionary events, which inevitably inflated the branch lengths. Furthermore, regardless of the presence of CNAs, doublets barely impacted the inference of branch lengths, though they impaired the inference of tree structure, resulting in increased normalized RF distance.

Overall, DelSIEVE demonstrated robustness to moderate levels of CNAs and doublets. Only when CNAs were excessively abundant in the input data did they significantly affect the inference of deletion-related genotypes, single ADOs, and tree branch lengths. In contrast, smaller amounts of CNAs had minimal impact on these aspects. Doublets primarily influenced the accuracy of tree structure inference. Notably, for the detection of single and double mutant genotypes, DelSIEVE remained fully robust under both CNA and doublet conditions.

### Runtime of DelSIEVE differs from SIEVE by only a constant

The time complexity analysis for the likelihood computation of DelSIEVE and SIEVE indicated the same worst-case complexity ($\mathcal {O}(I J K^2)$; see DelSIEVE likelihood section in Additional file 1: Supplementary notes), conditional on the same number of MCMC iterations. DelSIEVE, given the same number of candidate variant sites (*I*) and cells (*J*), is expected to obtain longer runtime by only a constant of 3, due to the expanded genotype state space of seven genotypes ($K = 7$), compared SIEVE with four genotypes ($K = 4$). Indeed, in such a case the time complexity of likelihood computation for DelSIEVE is $7^2 / 4^2 \approx 3$ times greater than that for SIEVE. To empirically compare the runtimes between DelSIEVE and SIEVE under the default multithreading mode (Additional file 2: Fig. S18; see Runtime analysis section in Supplementary notes), we designed two simulation scenarios, each with 100 cells and five replicates, where one scenario had median number 798 of candidate variant sites and the other had median number 1585 of candidate variant sites (Additional file 2: Fig. S18a). We ran DelSIEVE and SIEVE with both stages for $10^5$ iterations, with the same number of sites per thread. The results showed that the runtimes of both stages of DelSIEVE were around three times longer than those of SIEVE, regardless of the number of candidate variant sites in the input data (Additional file 2: Fig. S18b). This observation was in agreement with the above theoretical runtime estimates.

### DelSIEVE identifies deletions in triple negative breast cancer (TNBC) cells

**Fig. 4:**
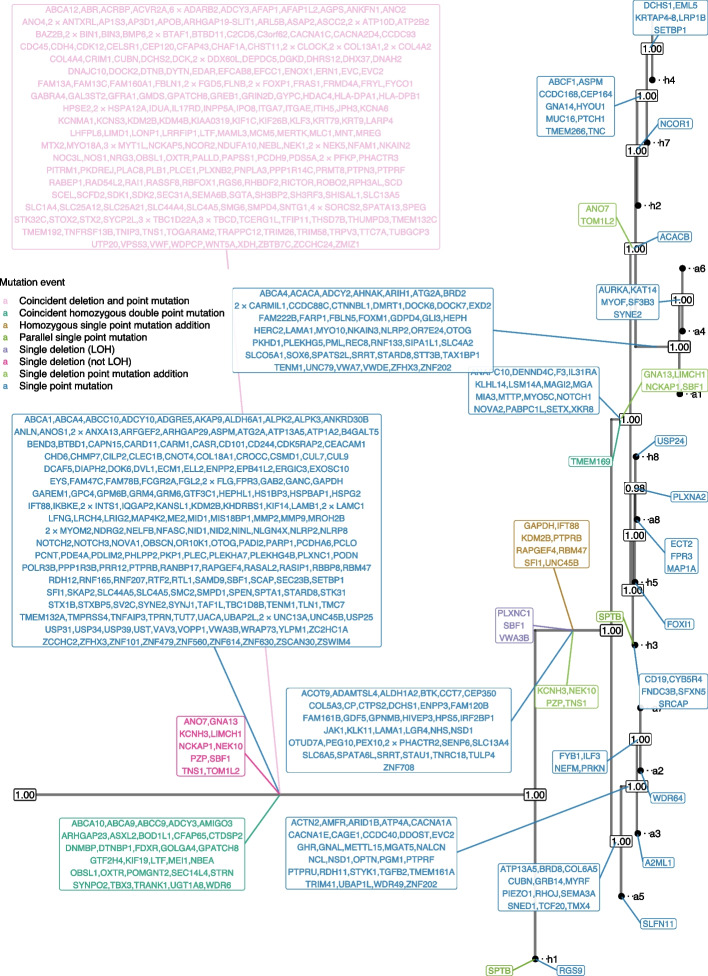
Results of phylogenetic inference for the TNBC16 dataset. Shown is DelSIEVE’s maximum clade credibility tree. Tumor cell names are annotated to the leaves of the tree. The numbers at each node represent the posterior probabilities (threshold $p> 0.5$). At each branch, depicted in different colors are non-synonymous genes that are either TNBC-related single mutations (in blue) or other mutation events (in other colors; see the legend)

We applied DelSIEVE to real scDNA-seq datasets previously analyzed using SIEVE [32] (see Configurations of methods section in Additional file 1: Supplementary notes). For the single-cell whole-exome sequencing (scWES) dataset TNBC16, containing data for 16 cells [35], DelSIEVE reported a maximum clade credibility cell phylogeny with a long trunk and with high posterior probabilities for most nodes (Fig. 4, Additional file 2: Fig. S19). The cell phylogeny was very similar to that reported by SIEVE, with the normalized RF and BS distances being 0.07 and $3.88 \times 10^{-6}$, respectively.

We first compared the distribution of mutation event types in non-synonymous genes as reported by DelSIEVE and SIEVE (Additional file 2: Fig. S20a). DelSIEVE identified a total of 689 mutation events, substantially more than the 454 ones detected by SIEVE. This indicates that DelSIEVE offers improved sensitivity in mutation detection. Notably, approximately 39% of the events identified by DelSIEVE were associated with various forms of deletions—including single deletion (both LOH and non-LOH), single deletion point mutation addition, and coincident deletion and point mutation events. These categories fall outside the detection capability of SIEVE, highlighting the enhanced scope of DelSIEVE in capturing complex mutation events.

DelSIEVE identified the same types of mutation events as reported by SIEVE, except for back mutations. In terms of numbers, DelSIEVE explained the same data with fewer point mutations. Specifically, DelSIEVE identified 31 coincident homozygous double point mutations (transitions from 0/0 to 1/1; 44 for SIEVE), eight homozygous single point mutation additions (from 0/1 to 1/1; nine for SIEVE), and two parallel single point mutations (from 0/0 to 0/1 that occurred more than once in the tree; same for SIEVE). SIEVE found seven single back mutations (from 0/1 to 0/0; *BRD8*, *COL6A5*, *GRB14*, *MYRF*, *RHOJ*, *SEMA3A*, *TMX4*), which were gained in the trunk and lost afterward in the branch leading to the sibling clade of a2/a3/a5/a7 clade. In contrast, DelSIEVE identified those as unique mutations that occurred only in the ancestor of this clade.

In addition, DelSIEVE identified several deletions, including a large number of 245 coincident deletions and point mutations (from 0/0 to 1/-), three single deletions which could be categorized as LOH (from 0/1 to 0/- or 1/-, or from $1/1^{\prime }$ to 1/-), ten single deletions which were not LOH (from 0/0 to 0/-, or from 1/1 to 1/-), and finally ten single deletion point mutation additions (from 0/- to 1/-). For instance, DelSIEVE inferred that gene *NEK1* and *NEK5*, which had been reported to be related to breast tumors [36], experienced both a deletion and a mutation on the trunk, resulting in all sequenced cells having genotype 1/-. Another gene, *LIMCH1*, known to be related to TNBC [37], had an allele deleted first on the trunk (genotype changed from 0/0 to 0/-), and then the remaining allele mutated for a subgroup of cells (genotype changed from 0/- to 1/-). Additionally, by referring to the COSMIC database [38] (https://cancer.sanger.ac.uk), we found that four tumor suppressor genes, namely *ACVR2A*, *CDK12*, *NCOR2*, and *ROBO2*, had both a deletion and point mutation simultaneously in the trunk of the tree, indicating that they might have lost their normal functionalities. The substantial amount of evolutionary events related to deletions highlights the importance of the extended functionality of DelSIEVE as compared to SIEVE.

**Fig. 5:**
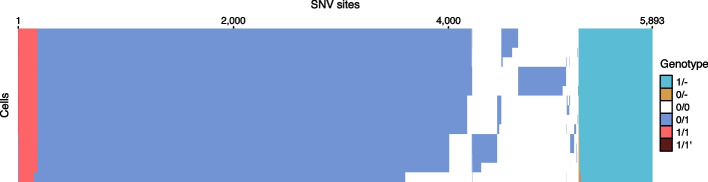
Results of variant calling for the TNBC16 dataset. Cells in the rows are in the same order as that of leaves in the phylogenetic tree in Fig. 4

In total, DelSIEVE identified 5893 variant sites, close to the 5895 variant sites reported by SIEVE (Fig. 5). Among the 683 sites inferred by DelSIEVE that contain deletions (mostly 1/-; 11.6% of all variant sites), 377 were previously determined according to SIEVE to have double mutants and the remaining 306 to have a single mutant genotype. This observation was in accordance with the simulation results, where SIEVE tended to explain deletions as dropout events within single and double mutants. The proportions of different genotypes called by DelSIEVE and SIEVE are summarized in Additional file 3: Table S4 (same for the following datasets).

### DelSIEVE identifies rare mutations in colorectal cancer (CRC) cells

We then applied DelSIEVE to a scWGS dataset, CRC28 [32], containing data for 28 single cells coming from three biopsies: tumor proximal (TP; with nine cells), tumor distal (TD; with seven cells), and tumor central (TC; with 12 cells). The estimated cell phylogeny was supported by high posterior probabilities with a long trunk (Additional file 2: Figs. S21, S22), which was similar to that reported by Kang et al. using SIEVE (the normalized RF and the BS distances were 0.08 and $8.03 \times 10^{-7}$, respectively). In particular the TP and TD subclones also formed sister clades in the tree reported by DelSIEVE, with the TC subclone forming a separate clade, suggesting regular tumor growth and limited cell migration.

DelSIEVE overall found comparable, but slightly more mutation events in non-synonymous genes than SIEVE did (88 against 85; Additional file 2: Fig. S20b). Similarly to SIEVE, DelSIEVE identified mutations in known CRC driver genes, for instance, *APC*, and of genes related to the metastatic progression of CRC, such as *ASAP1* and *RGL2* on the trunk of the tree. However, DelSIEVE identified a subset of mutation events that were not detected by SIEVE, comprising approximately 5% of the total events, including two coincident deletions and point mutations, one single deletion which was not LOH, and one single deletion mutation addition. For example, DelSIEVE identified that *ACSL5*, potentially related to intestinal carcinogenesis [39], underwent a somatic deletion of one allele (genotype changed from 0/0 to 0/-) on the trunk and a point mutation to the remaining allele (genotype changed from 0/- to 1/-) for the most recent common ancestor of TP and TD subclones.

DelSIEVE identified the same number of variant sites as SIEVE (8,029; see Additional file 2: Fig. S23), in which 13 sites contained deletions (mostly 1/-; 0.16% of all variant sites). According to SIEVE, nine of those sites were inferred to have double mutants and four to have single mutants. Overall, both DelSIEVE and SIEVE found very few mutation events that were not single mutations. The contrasting results obtained by DelSIEVE for TNBC16 (with multiple deletions) compared to CRC28 (only few deletions) underscored an important feature of the method. While DelSIEVE employs a sophisticated and expressive modeling approach, it primarily relies on the data for the inference, without “overcalling” deletions.

### DelSIEVE identified rare somatic mutations in CRC samples mixed with normal cells

We finally analyzed another scWES dataset, CRC48 (CRC0827 in [40]), with cells collected at three anatomical locations: adenomatous polyps (containing 13 normal cells), cancer tissue 1 (17 cells), and cancer tissue 2 (18 cells). DelSIEVE pinpointed two tumor subclones, associated with their anatomical locations, each subclone containing exactly the same cells as found by Kang et al. using SIEVE (Additional file 2: Figs. S24, S25). The rest of the cells collected from tumor biopsies were clustered together with cells from adenomatous polyps, suggesting that they might be normal cells residing inside cancer tissues, as pointed out by both the original study [40] and Kang et al. [32]. There were some distinctions between the cell phylogenies reported by DelSIEVE and SIEVE, with normalized RF and BS distances being 0.33 and $1.99 \times 10^{-6}$, respectively. This discrepancy is higher than observed for the previous datasets and might be due to the overall lower signal level in the data. Indeed, the CRC48 dataset has a substantially lower ratio between the number of candidate variant sites and the number of cells ($707 / 48 \approx 14.7$) compared to TNBC16 ($5912 / 16 = 369.5$) and CRC28 ($8470 / 28 = 302.5$), and therefore contains potentially less phylogenetic information.

DelSIEVE found fewer mutation events in non-synonymous genes for this dataset compared to SIEVE (141 against 148; Additional file 2: Fig. S20c), and none of them was related to deletions. Comparison of the colorectal cancer to TNBC results indicates that DelSIEVE identifies more evolutionary events than SIEVE only when they are substantially evidenced in the data. Thus, despite higher expressibility, DelSIEVE does not necessarily find more complex evolutionary histories and genotypes than SIEVE. DelSIEVE identified many single point mutations on the branch leading to the two tumor subclones, including a reported CRC driver mutation in gene *SYNE1* [41], as well as a mutation related to DNA mismatch repair, in gene *MLH3* [42]. DelSIEVE also found two parallel single point mutations (*CHD3* and *PLD2*). Furthermore, DelSIEVE identified only one site containing deletions (among 679 variant sites, and only 0/-; see Additional file 2: Fig. S26), which was previously inferred by SIEVE to have a single mutant genotype.

### Sequencing coverage agrees with deletion sites identified by DelSIEVE

**Fig. 6:**
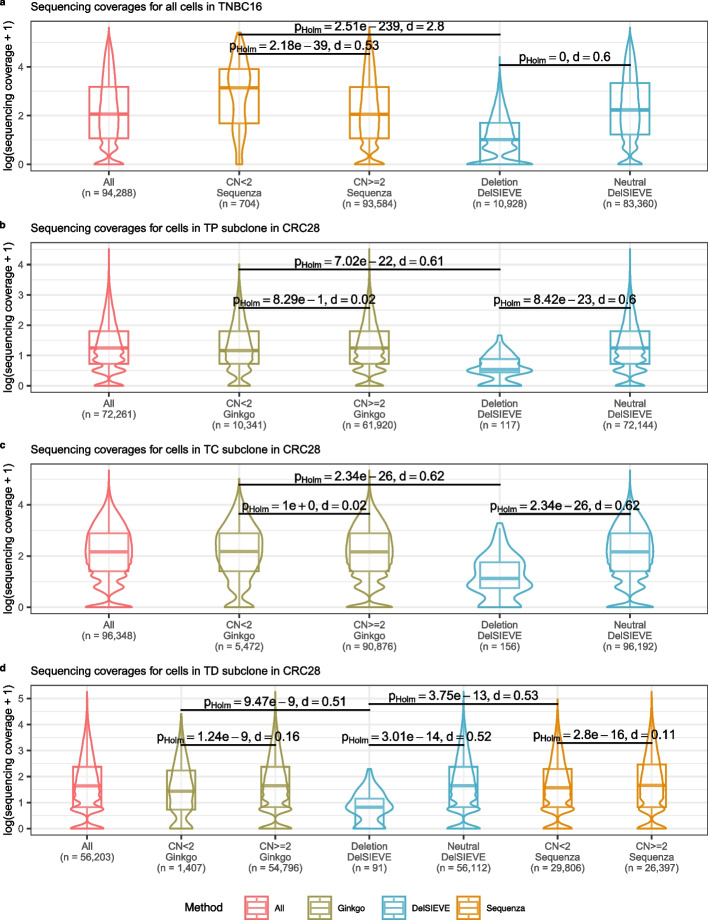
Comparison of sequencing coverage on clone (for TNBC16) and subclone (for CRC28) level. Compared were the sites shared in the output of DelSIEVE, Sequenza [43], and Ginkgo [44], if available. For Sequenza and Ginkgo, sites were divided into two groups with copy number (CN) $< 2$ and $\ge 2$. For DelSIEVE, sites were also divided into two groups, one with deletions, the other copy neutral. Sequencing coverage transformed with $\log {p1}$ across all cells in the clone or subclone at all sites were plotted for reference. In each group, the violin and the box plots matched the color of the method. The total number of data points in each group was marked with *n* on the horizontal axis. Box plots comprise medians, boxes covering the interquartile range (IQR), and whiskers extending to 1.5 times the IQR below and above the box. Within- and between-group comparisons were conducted between CN $< 2$ and $\ge 2$ of Sequenza and Ginkgo, between deletions and copy neutral of DelSIEVE, and between deletions of DelSIEVE and CN $< 2$ of Sequenza and Ginkgo. Each comparison was conducted on the sequencing coverage on the original scale, showing the result of two-sided Mann-Whitney *U* test, with the *p *value corrected by Holm–Bonferroni method, and the absolute value of the effect size (Cohen’s *d*). Comparison of sequencing coverage for all cells in TNBC16 (**a**) as well as in TP (**b**), TC (**c**), and TD (**d**) subclones in CRC28

To further validate the ability of DelSIEVE to reliably call deletions, we inspected whether the sites identified as deleted in the analyzed datasets had lower coverage than sites lacking deletions. We next compared the strength of the coverage reduction effect on deleted sites with the results from dedicated copy number calling methods, Sequenza [43] (applicable to bulk sequenced samples from TNBC16, TD subclone from CRC28, and cancer tissue 1 and 2 from CRC48), as well as Ginkgo [44] (Fig. 6) (applicable to WGS of single cells in CRC28). The comparison was performed only for the candidate variant sites, and the raw sequencing coverage was scaled by the corresponding size factors of the single cells.

Since Sequenza was designed for bulk-seq data, we adjusted the resolution of DelSIEVE’s and Ginkgo’s results with Sequenza for the sake of comparison. To this end, a site in a given sample was called a variant site (a deletion for DelSIEVE, deletion or amplification for Gingko) if the method identified that variant in at least one cell from the sample. All other sites were considered neutral, where no cells had deletions or amplifications.

For the TNBC16 dataset the mean value of sequencing coverage in the group of sites with deletions identified by DelSIEVE (3.59) was significantly lower compared to the mean for sites without deletions (22.04), with effect size Cohen’s *d* $= 0.6$ (Fig. 6a). In contrast, the mean coverage for 44 sites identified as containing deletions by Sequenza was 36.13, significantly larger than 19.78, the mean coverage for sites with amplifications (Cohen’s *d* $= 0.53$), controverting Sequenza’s copy number calls for the TNBC16 dataset. Furthermore, a direct comparison revealed that sites identified as deleted by DelSIEVE showed much lower coverage levels than those identified as deleted by Sequenza (Cohen’s *d* $= 2.8$).

For the CRC28 dataset and the TP subclone (Fig. 6b) as well as the TC subclone (Fig. 6c), the mean coverage at sites with deletions called by DelSIEVE was significantly lower than that from sites without deletions (Cohen’s *d* $= 0.6$ and 0.62 for TP and TC subclones, respectively). However, such differences were not significant for Ginkgo (Cohen’s *d* $= 0.02$ for both TP and TC subclones).

For the TD subclone (Fig. 6d), DelSIEVE, Ginkgo, and Sequenza had a lower mean coverage for sites with deletions compared to those without, where DelSIEVE exhibited a more evident distinction (Cohen’s *d* $= 0.52$) than Ginkgo and Sequenza (Cohen’s *d* $= 0.16$ and 0.11, respectively). Moreover, for sites containing deletions called by the three methods, DelSIEVE had the lowest mean coverage, which was significantly different from the mean coverages of Ginkgo and Sequenza (Cohen’s *d* $= 0.51$ and 0.53, respectively).

We also visualized across the entire genome the reported CNs of Ginkgo in all cells and of Sequenza in TD subclone (Additional file 2: Fig. S27a, b). Based on the CNs called by Ginkgo, it was evident that the phylogenetic distance between TP and TD subclones was shorter than that between either of them and TC subclone, as in the tree reported by DelSIEVE (Additional file 2: Figs. S21, S22). Moreover, although Sequenza inferred a majority of deletions, Ginkgo only inferred a small number of deletions, in accordance with the results of DelSIEVE, where only few deletions were identified.

Finally, for the CRC48 dataset, sites with and without deletions identified by DelSIEVE showed a pronounced mean coverage difference, for both cancer tissue 1 (Additional file 2: Fig. S28; Cohen’s *d* $= 0.39$) and cancer tissue 2 (Additional file 2: Fig. S29; *d* $= 0.48$). The mean coverage difference between sites identified as deleted or not by Sequenza was negligible for both subclones (Cohen’s *d* $= 0.04$ for cancer tissue 1; *d* $= 0.09$ for cancer tissue 2). Moreover, the mean coverage was much lower for sites identified to carry deletions by DelSIEVE than for sites identified as carrying deletions by Sequenza (Cohen’s *d* $= 0.45$ for cancer tissue 1; *d* $= 0.51$ for cancer tissue 2).

### DelSIEVE showed good convergence metrics with well-mixed chains

We additionally diagnosed the convergence of the DelSIEVE model by running an additional MCMC chain for each real dataset with a different random seed and dispersed starting values. Trace plots of the log-likelihood and several hidden random variables, in the order they were sampled across both chains, provided clear visual evidence of convergence (Additional file 2: Figs. S30–S32). These plots indicated that the MCMC chains in DelSIEVE mixed well and explored the parameter space efficiently, sampling from the same target distribution well before the burn-in threshold. We removed samples from the burn-in phase for both chains, which were the first 10% of all samples. The remaining samples, which were assumed to come from the target posterior distribution, were used to compute the Gelman-Rubin statistic [45] to quantitatively evaluate convergence (Additional file 2: Fig. S33). The Gelman-Rubin statistic of every hidden random variable for each real dataset was close to 1, supporting the conclusion that the chains had likely converged. Taken together, these qualitative and quantitative convergence metrics suggested that DelSIEVE achieved efficient mixing and convergence. However, as with all convergence metrics, we note that these diagnostics provide strong evidence but cannot absolutely guarantee full exploration of the posterior.

## Discussion

We present DelSIEVE, a statistical method designed to jointly infer deletions, SNVs, and the cell phylogeny from scDNA-seq data. Built upon SIEVE, which combines the inference of SNVs and the cell phylogeny, DelSIEVE takes a step forward by also considering point deletions. In a nutshell, DelSIEVE features a statistical phylogenetic model with genotypes relating both to deletions and to single and double mutants, a model of raw read counts allowing for both ADO and LDO, and a mechanism for acquisition bias correction for the branch lengths. Moreover, DelSIEVE effectively captures additional uncertainties, for instance, errors due to systematic context-dependent PCR amplification. These non-random errors occur more often at specific DNA sequence motifs, and the Dirichlet-Multinomial distribution used in DelSIEVE effectively models this overdispersion by allowing probabilities to vary across sites and cells, reflecting both average error rates and extra variability from context effects.

Deletions often play an essential role in tumor evolution. We have shown that SIEVE tends to explain deletions as a result of dropouts and overestimates the amount of single and double mutants. Compared to SIEVE, DelSIEVE exhibits improved performance in terms of calling double mutants, while performing similarly in estimating the cell phylogeny and calling single mutants.

The difficulty of identifying deletions from scDNA-seq data is mainly due to the fact that dropouts and uneven coverage, prevalent in this type of data, can also decrease the observed coverage at a site. DelSIEVE is the only method capable of discerning detailed types of deletions such as alternative-remaining, reference-remaining, and double deletions. DelSIEVE outperforms Monovar, SCIPhIN, and SIEVE in variant calling on simulated data. With high enough coverage quality, DelSIEVE outperforms the only other approach, SIEVE, for ADO and LDO calling. When applied to three real scDNA-seq datasets from TNBC and CRC samples, which were previously analyzed using SIEVE, DelSIEVE identified rare deletions and double mutants in the CRC samples, akin to the results of SIEVE. However, for the TNBC dataset, DelSIEVE identified multiple deletions while revealing fewer single and double mutants compared to SIEVE, consistent with the benchmarking results.

A potential improvement to DelSIEVE would be to add the identification of insertions. Moreover, the current procedure for preselecting the candidate variant sites is limited to those sites that potentially contain nucleotide substitutions. To address this limitation, a possible enhancement would be to enable this procedure to preselect sites of tumor suppressor genes that are solely associated with deletions. The inclusion of these sites, which are known to elevate the risk of tumor development [3, 46], could further refine DelSIEVE’s utility in understanding tumorigenesis and potential therapeutic targets. Furthermore, an important assumption underlying DelSIEVE is that the genomic sites are independent for computational reasons. However, this assumption is violated for copy number aberrations (CNAs). Thus, DelSIEVE is designed only for the identification of point mutations, not for the detection of copy number changes in consecutive genomic regions. It is worth emphasizing that as a method for the joint inference of SNVs, deletions, and cell phylogeny, DelSIEVE should ideally be applied on scDNA-seq data amplified with isothermal-based methods, which offer high coverage across the genome but low uniformity of sequencing coverage, suitable for calling SNVs instead of CNAs.

Despite these limitations, DelSIEVE is one of the most sophisticated statistical phylogenetics models available. The expanded capabilities of DelSIEVE make it a valuable tool for unraveling complex genomic dynamics and understanding evolutionary relationships among cells.

## Conclusions

DelSIEVE is a novel probabilistic model that from raw read counts of scDNA-seq data jointly infers cell phylogeny and somatic variants, including SNVs and their deletions. We prove in our simulations that DelSIEVE is able to reliably differentiate several types of deletions and SNVs, while also reporting highly credible cell phylogenies. DelSIEVE can also call different types of dropout events, namely ADOs and LDOs, provided that the data is of enough quality, which is highly promising as the technology continues to advance. The application of DelSIEVE is not limited to tumors; the model can also be employed to investigate evolutionary dynamics in other tissue types.

## Methods

### Statistical phylogenetic model behind DelSIEVE

For the genotype state space $G = \{ 0/0, 0/1, 1/1, 1/1^{\prime }, \text {0/-}, \text {1/-}, \text {-} \}$ given for the DelSIEVE model, we define the instantaneous transition rate matrix *Q* as visualized in Fig. 1c. We set the somatic mutation rate to 1 [47], where the relative measurements for the back mutation rate and deletion rate are $\frac{1}{3}$ and *d*, respectively. Thus, *Q* is deterministic and depends on the value of the relative deletion rate *d*, namely $P \left( Q \,|\, d \right) = 1$. Each entry in *Q* represents the transition rate from the genotype in the row to that in the column during an infinitesimal time $\Delta t$, while each row in *Q* sums up to 0. The continuous-time homogeneous Markov chain underlying *Q* is time non-reversible and reducible. For instance, genotypes that have both alleles present can transition to genotypes with one or both alleles lost, but not vice versa. To be specific, genotypes $\{ 0/0, 0/1, 1/1, 1/1^{\prime } \}$ and genotypes $\{ \text {0/-}, \text {1/-} \}$ form two ergodic, transient communicating classes, while genotype $\{ \text {-} \}$ forms a closed communicating class. As a result, the limiting distribution of the Markov chain exists, where the value corresponding to genotype - is 1, while the others are 0.

Denote by $g_{ij}$ the hidden variable describing the genotype for site $i \in \{ 1, \dots , I \}$ in cell $j \in \{ 1, \dots , J \}$. Based on the well-established theory of statistical phylogenetic models [47], the joint conditional probability of the genotype states of all sequenced cells at site *i*, namely $\varvec{g}^{(L)}_{i}$, is

1 $$\begin{aligned} P \left( \left. \varvec{g}^{(L)}_{i} \,\right| \, \mathcal {T}, \varvec{\beta }, Q, h, \eta \right) = \sum \limits _{\varvec{g}^{(A)}_{i} \setminus \left\{ g_{i(2J)} \right\} } P \left( \varvec{g}^{(L)}_{i}, \varvec{g}^{(A)}_{i} \setminus \left\{ \left. g_{i(2J)} \right\} \,\right| \, \mathcal {T}, \varvec{\beta }, Q, h, \eta \right) . \end{aligned}$$

Intuitively, this means that to compute the likelihood of the genotypes of the variant sites at the leaves, we marginalize out the genotypes at the ancestor nodes from the total likelihood. The variables in Eq. (1) have the following meaning: $\mathcal {T}$ is the rooted binary tree topology, whose root, representing a normal cell with diploid genome, has only one child, the most recent common ancestor (MRCA) of all sequenced cells. $\mathcal {T}$ has *J* sequenced cells as leaves, labeled by $\{ 1, \dots , J \}$, and *J* extinct, ancestor cells as internal nodes, labeled by $\{ J + 1, \dots , 2J \}$, where node 2*J* is the root of $\mathcal {T}$. The leaves have genotypes $\varvec{g}^{(L)}_{i} = (g_{i1}, \dots , g_{ij}, \dots , g_{iJ})^T$, where $g_{ij} \in G$, while the internal nodes have genotypes $\varvec{g}^{(A)}_{i} = \left( g_{i(J+1)}, \dots , g_{ij}, \dots , g_{i(2J)} \right) ^T$, where $g_{ij} \setminus \left\{ g_{i(2J)} \right\} \in G$ and $g_{i(2J)} = 0/0$. $\mathcal {T}$ has $2J - 1$ branches, whose lengths $\varvec{\beta } \in \mathbb {R}^{2J - 1}$ represent the expected number of somatic mutations per site. *h* and $\eta$ are the number of rate categories and shape, respectively, of a discrete Gamma distribution with mean equal 1 for modeling among-site substitution rate variation [48]. Hidden random variables $d, \mathcal {T}, \varvec{\beta }, \eta$ are estimated using MCMC from the posterior of the samples, while the fixed hyperparameter *h* takes value 4 by default.

Given deletion rate *d* (and thus *Q*) and branch length $\beta$, the seven-by-seven transition probability matrix $R(\beta )$ is computed as $R(\beta ) = \exp {(Q \beta )}$ [47].

### Model of raw read counts behind DelSIEVE

DelSIEVE’s input data for each cell $j \in \{ 1, \dots , J \}$ at each candidate site $i \in \{ 1, \dots , I \}$ comes in the form of $\mathcal {D}_{ij}^{(1)} = (\varvec{m}_{ij}, c_{ij})$, where $\varvec{m}_{ij} = \{ m_{ijk} \,|\, k = 1,2,3 \}$ are the read counts of three alternative nucleotides with values in descending order and $c_{ij}$ is the sequencing coverage (Fig. 1a; see Kang et al. [32] for explanation of how candidate sites are identified). For acquisition bias correction [49, 50], DelSIEVE also optionally takes raw read count data $\mathcal {D}^{(2)}$ from $I^\prime$ background sites that have a wildtype genotype.

We factorize the probability of observing raw read counts $\mathcal {D}_{ij}$ for cell *j* at site *i* into

2 $$\begin{aligned} P(\mathcal {D}_{ij}) = P(\varvec{m}_{ij} \,|\, c_{ij}) P(c_{ij}), \end{aligned}$$

where the former corresponds to the model of nucleotide read counts and the latter to the model of sequencing coverage.

#### Model of sequencing coverage.

A major, yet often overlooked, challenge in scDNA-seq is the highly uneven sequencing coverage. This happens because the genetic materials are amplified largely unequally during WGA. Similar to SIEVE, we employ a negative binomial distribution to capture the overdispersion existing in the sequencing coverage:

3 $$\begin{aligned} P(c \,|\, p, r) = \left( \begin{array}{c} {c+r-1} \\ {r-1}\end{array}\right) p^r (1-p)^c, \end{aligned}$$

where *p* and *r* are parameters. To improve interpretability, the distribution is reparameterized using mean $\mu$ and variance $\sigma ^2$:

4 $$\begin{aligned} \left\{ \begin{array}{l} p = \frac{\mu }{\sigma ^2},\\ r = \frac{\mu ^2}{\sigma ^2 - \mu }. \end{array}\right. \end{aligned}$$

We assume that $\mu _{ij}$ and $\sigma ^2_{ij}$ have the same form as in SIEVE, namely

5 $$\begin{aligned} \mu _{ij} & = \alpha _{ij} t s_j + \epsilon ,\nonumber \\ \sigma ^2_{ij} & = \mu _{ij} + \alpha ^2_{ij} \nu s_j^2. \end{aligned}$$

Here, *t* and $\nu$ are the mean and the variance of allelic coverage, respectively. $\alpha _{ij} \in \{ 0, 1, 2 \}$ represents the number of sequenced alleles. $\epsilon$ is a small number that is used to stabilize the computation in the case when $\alpha _{ij} = 0$, and by default we set $\epsilon = 10^{-6}$. With the extended genotype state space *G* in the DelSIEVE model, the true number of alleles at a site can either be zero (corresponding to genotype state $\{ \text {-} \}$), one (genotype states $\{ \text {0/-}, \text {1/-} \}$), or two ($\{ 0/0, 0/1, 1/1, 1/1^{\prime } \}$). On top of that, the possible occurrence of dropouts during scWGA could also alter the number of observed alleles at a site. Here, we model two types of dropout modes, the loss of one of the two alleles at a site (ADO) or the loss of both alleles (LDO). In ADO simulations and model configuration, only ADO events can occur. In LDO mode, both ADO and LDO may occur. The detailed description of ADO and LDO modes in DelSIEVE is in Additional file 1: Supplementary notes.

In Eq. (5), $s_j$ is the size factor of cell *j*, which makes sequencing coverage from different cells comparable, and which is estimated using

6 $$\begin{aligned} \hat{s}_j = \underset{i:c_{ij} \ne 0}{\text {median}} \frac{c_{ij}}{\left( \prod \nolimits _{\begin{array}{c} j^\prime = 1 \\ c_{ij^\prime } \ne 0 \end{array}}^{J^\prime } c_{ij^\prime } \right) ^{\!\frac{1}{J^\prime }}}, \end{aligned}$$

where $J^\prime$ is the number of cells with non-zero coverage at a site.

#### Model of nucleotide read counts.

The occurrence of dropouts could change the number of alleles sequenced for cell *j* at site *i*. As a result, the observed genotype $g_{ij}^\prime \in G$ could be different from the true genotype $g_{ij}$. The probability of $g_{ij}^\prime$ is $P(g_{ij}^\prime \,|\, g_{ij}, \alpha _{ij})$, which is defined in Table 1 for the ADO mode and in Table 2 for the LDO mode.

**Table 1:** Definition of the distribution of the observed genotype $g_{ij}^\prime$ conditional on the true genotype $g_{ij}$ and number of sequenced alleles $\alpha _{ij}$ under the ADO mode

$\varvec{g}_{\varvec{ij}}^{\varvec{\prime }}$	$\varvec{g}_{\varvec{ij}}$	$\varvec{\alpha }_{\varvec{ij}}$	$\varvec{P(g}_{\varvec{ij}}^{\varvec{\prime }} \,\varvec{|}\, \varvec{g}_{\varvec{ij}}\varvec{, \alpha }_{\varvec{ij}}\varvec{)}$
0/0	0/0	2	1
0/-	0/0	1	1
0/1	0/1	2	1
0/-	0/1	1	$\frac{1}{2}$
1/-	0/1	1	$\frac{1}{2}$
1/1	1/1	2	1
1/-	1/1	1	1
$1/1^{\prime }$	$1/1^{\prime }$	2	1
1/-	$1/1^{\prime }$	1	1
0/-	0/-	1	1
-	0/-	0	1
1/-	1/-	1	1
-	1/-	0	1
-	-	0	1
Others			0

**Table 2:** Definition of the distribution of the observed genotype $g_{ij}^\prime$ conditional on the true genotype $g_{ij}$ and number of sequenced alleles $\alpha _{ij}$ under the LDO mode

$\varvec{g}_{\varvec{ij}}^{\varvec{\prime }}$	$\varvec{g}_{\varvec{ij}}$	$\varvec{\alpha }_{\varvec{ij}}$	$\varvec{P(g}_{\varvec{ij}}^{\varvec{\prime }} \,\varvec{|}\, \varvec{g}_{\varvec{ij}}\varvec{, \alpha }_{\varvec{ij}}\varvec{)}$
0/0	0/0	2	1
0/-	0/0	1	1
-	0/0	0	1
0/1	0/1	2	1
0/-	0/1	1	$\frac{1}{2}$
1/-	0/1	1	$\frac{1}{2}$
-	0/1	0	1
1/1	1/1	2	1
1/-	1/1	1	1
-	1/1	0	1
$1/1^{\prime }$	$1/1^{\prime }$	2	1
1/-	$1/1^{\prime }$	1	1
-	$1/1^{\prime }$	0	1
0/-	0/-	1	1
-	0/-	0	1
1/-	1/-	1	1
-	1/-	0	1
-	-	0	1
Others			0

When $g_{ij}^\prime \in G \setminus \{ \text {-} \}$, we model $\varvec{m}_{ij}$, the read counts of three alternative nucleotides, conditional on the sequencing coverage $c_{ij}$ as a Dirichlet-Multinomial distribution:

7 $$\begin{aligned} P(\varvec{m}_{ij} \,|\, c_{ij}, \varvec{a}_{ij}) = \frac{F(c_{ij}, a_{ij0})}{\prod \nolimits _{k = 1:m_{ijk}> 0}^3 F(m_{ijk}, a_{ijk}) F(c_{ij} - \sum \nolimits _{k = 1}^3 m_{ijk}, a_{ij4})}, \end{aligned}$$

with parameters $\varvec{a}_{ij} = \{ a_{ijk} \,|\, k = 1, \dots , 4 \}$ and $a_{ij0} = \sum \nolimits _{k = 1}^4 a_{ijk}$. *F* is a function defined as

8 $$\begin{aligned} F(x,y) = \left\{ \begin{array}{l} x B(y, x),\ \text {if}\ x> 0,\\ 1,\ \text {otherwise,} \end{array}\right. \end{aligned}$$

where *B* is the beta function. Note that $c_{ij} - \sum \nolimits _{k = 1}^3 m_{ijk}$ is the read count of the reference nucleotide.

We reparameterize Eq. (7) by letting $\varvec{a}_{ij} = w_{ij} \varvec{f}_{ij}$. $w_{ij}$ captures the overdispersion in the assignment of $c_{ij}$ read counts among all nucleotides. $\varvec{f}_{ij} = \{ f_{ijk} \,|\, k = 1, \dots , 4 \}$, $\sum \nolimits _{k = 1}^4 f_{ijk} = 1$ is a vector of expected frequencies of each nucleotide, where the first three elements correspond to the three alternative nucleotides ordered decreasingly according to their read counts, and the last to the reference nucleotide. Depending on $g_{ij}^\prime$, $\varvec{f}_{ij}$ is given by

9 $$\begin{aligned} \varvec{f}_{ij} = \left\{ \begin{array}{l} \varvec{f}_1 = \left( \frac{1}{3} f, \frac{1}{3} f, \frac{1}{3} f, 1 - f \right) ,\ \text {if}\ g_{ij}^\prime = 0/0\ \text {or}\ \text {0/-}, \\ \varvec{f}_2 = \left( \frac{1}{2} - \frac{1}{3} f, \frac{1}{3} f, \frac{1}{3} f, \frac{1}{2} - \frac{1}{3} f \right) ,\ \text {if}\ g_{ij}^\prime = 0/1, \\ \varvec{f}_3 = \left( 1 - f, \frac{1}{3} f, \frac{1}{3} f, \frac{1}{3} f \right) ,\ \text {if}\ g_{ij}^\prime = 1/1\ \text {or}\ \text {1/-}, \\ \varvec{f}_4 = \left( \frac{1}{2} - \frac{1}{3} f, \frac{1}{2} - \frac{1}{3} f, \frac{1}{3} f, \frac{1}{3} f \right) ,\ \text {if}\ g_{ij}^\prime = 1/1^{\!\prime }, \end{array}\right. \end{aligned}$$

where *f* is the effective sequencing error rate, combining together amplification and sequencing errors. Note that amplification and sequencing errors, occurring during WGA phase and actual sequencing stage, respectively, are not modeled independently in our model. The reason is that even though they are different types of errors and occur independently at distinct technical stages, they have similar impacts to target nucleotides. Modeling them independently without additional information will likely result in non-identifiability issues. Thus, we model these two types of artifacts with a compound hidden random variable, *f*, to reflect the similarities of the roles they play. This means that instead of their respective values, we focus on the value that represents their joint effects, and thus we name *f* “effective sequencing error rate”.

The parameter $w_{ij}$ also depends on $g_{ij}^\prime$, where

10 $$\begin{aligned} w_{ij} = \begin{array}{l} w_1,\ \text {if}\ g_{ij}^{\prime } = 0/0,\ \text {0/-},\ 1/1,\ \text {or}\ \text {1/-}, \\ w_2,\ \text {if}\ g_{ij}^{\prime } = 0/1\ \text {or}\ 1/1^{\prime }, \end{array} \end{aligned}$$

and $w_1$ is the overdispersion term when $g_{ij}^\prime$ has only one type of nucelotide, and $w_2$ is the term when $g_{ij}^\prime$ has different types of nucelotides.

By plugging Eqs. (9) and (10) into Eq. (7), and additionally defining

$$\begin{aligned} P(\varvec{m}_{ij} | c_{ij}, g_{ij}^\prime = \text {-}, f, w_{ij}) = 1, \end{aligned}$$

we obtain

11 $$\begin{aligned} P(\left. \varvec{m}_{ij} \right| c_{ij}, g_{ij}^\prime , f, w_{ij}) = \left\{ \begin{array}{l} P_{0/0} = P \left( \left. \varvec{m}_{ij} \,\right| \, c_{ij}, \varvec{f}_1, w_1 \right) , \;\text {if}\ g_{ij}^{\prime } = 0/0, \\ P_{\text {0/-}} = P \left( \left. \varvec{m}_{ij} \,\right| \, c_{ij}, \varvec{f}_1, w_1 \right) , \;\text {if}\ g_{ij}^\prime = \text {0/-}, \\ P_{0/1} = P \left( \left. \varvec{m}_{ij} \,\right| \, c_{ij}, \varvec{f}_2, w_2 \right) , \;\text {if}\ g_{ij}^\prime = 0/1, \\ P_{1/1} = P \left( \left. \varvec{m}_{ij} \,\right| \, c_{ij}, \varvec{f}_3, w_1 \right) , \;\text {if}\ g_{ij}^\prime = 1/1, \\ P_{\text {1/-}} = P \left( \left. \varvec{m}_{ij} \,\right| \, c_{ij}, \varvec{f}_3, w_1 \right) , \;\text {if}\ g_{ij}^\prime = \text {1/-}, \\ P_{1/1^{\prime }} = P \left( \left. \varvec{m}_{ij} \,\right| \, c_{ij}, \varvec{f}_4, w_2 \right) , \;\text {if}\ g_{ij}^\prime = 1/1^{\!\prime }, \\ P_{\text {-}} = P(\left. \varvec{m}_{ij} \right| c_{ij}, f, w_{ij}) = 1, \;\text {if}\ g_{ij}^\prime = \text {-}. \end{array}\right. \end{aligned}$$

### Mutation event classification

DelSIEVE is able to discern 28 types of genotype transitions, which are classified into 17 types of mutation events (Table 3). Each genotype transition is a possible combination of single point mutations, single back mutations and single deletions. Single point mutations happen when 0 mutates to 1, or 1 and $1^{\prime }$ mutate to each other. Single back mutations occur when 1 or $1^{\prime }$ mutates to 0. Single deletions happen when an existing allele is lost during evolution, namely 0 or 1 deleted.

**Table 3:** Twenty-eight types of genotype transitions that DelSIEVE is able to identify, with their interpretation as mutation events

Genotype transition	Mutation event	Identifiable by	
		DelSIEVE	SIEVE
$0/0 \rightarrow 0/1$	Single point mutation	Yes	Yes
$0/0 \rightarrow 1/1$	Coincident homozygous double point mutation	Yes	Yes
$0/0 \rightarrow 1/1^{\prime }$	Coincident heterozygous double point mutation	Yes	Yes
$0/1 \rightarrow 0/0$	Single back mutation	Yes	Yes
$1/1 \rightarrow 0/1$	Single back mutation	Yes	Yes
$1/1^{\prime } \rightarrow 0/1$	Single back mutation	Yes	Yes
$1/1 \rightarrow 0/0$	Coincident double back mutation	Yes	Yes
$1/1^{\prime } \rightarrow 0/0$	Coincident double back mutation	Yes	Yes
$0/1 \rightarrow 1/1$	Homozygous single point mutation addition	Yes	Yes
$0/1 \rightarrow 1/1^{\prime }$	Heterozygous single point mutation addition	Yes	Yes
$1/1^{\prime } \rightarrow 1/1$	Homozygous substitute single point mutation	Yes	Yes
$1/1 \rightarrow 1/1^{\prime }$	Heterozygous substitute single point mutation	Yes	Yes
$0/0 \rightarrow \text {0/-}$	Single deletion (not LOH)	Yes	No
$1/1 \rightarrow \text {1/-}$	Single deletion (not LOH)	Yes	No
$0/1 \rightarrow \text {0/-}$	Single deletion (LOH)	Yes	No
$0/1 \rightarrow \text {1/-}$	Single deletion (LOH)	Yes	No
$1/1^{\prime } \rightarrow \text {1/-}$	Single deletion (LOH)	Yes	No
$0/0 \rightarrow \text {1/-}$	Coincident deletion and point mutation	Yes	No
$1/1 \rightarrow \text {0/-}$	Coincident deletion and back mutation	Yes	No
$1/1^{\prime } \rightarrow \text {0/-}$	Coincident deletion and back mutation	Yes	No
$\text {0/-} \rightarrow \text {1/-}$	Single deletion point mutation addition	Yes	No
$\text {1/-} \rightarrow \text {0/-}$	Single deletion back mutation addition	Yes	No
$\text {0/-} \rightarrow \text {-}$	Single deletion addition	Yes	No
$\text {1/-} \rightarrow \text {-}$	Single deletion addition	Yes	No
$0/0 \rightarrow \text {-}$	Coincident double deletions	Yes	No
$0/1 \rightarrow \text {-}$	Coincident double deletions	Yes	No
$1/1 \rightarrow \text {-}$	Coincident double deletions	Yes	No
$1/1^{\prime } \rightarrow \text {-}$	Coincident double deletions	Yes	No

The genotype transitions correspond to possible changes of genotypes on a branch from the parent node to the child node. If any of these events occurs on independent branches of the phylogenetic tree, it is also considered as a parallel evolution event. The first 12 genotype transitions are also identifiable with SIEVE. LOH in the table represents loss of heterozygosity

Since DelSIEVE encompasses the genotype state space modeled by SIEVE, it is capable of discerning all genotype transitions that SIEVE can handle, namely the first 12 rows in Table 3 (for detailed explanation see Kang et al. [32]). The mutation events that only DelSIEVE is able to discern are explained as follows. Single deletions that happen when one allele is deleted from genotypes in which both alleles originally had different nucleotides result in loss of heterozygosity (LOH) ($0/1 \rightarrow \text {0/-}$, $0/1 \rightarrow \text {1/-}$, and $1/1^{\prime } \rightarrow \text {1/-}$). Deletions that take place when one allele is deleted from genotypes in which both alleles originally contained the same nucleotide do not result in LOH ($0/0 \rightarrow \text {0/-}$ and $1/1 \rightarrow \text {1/-}$). The coincident deletion and point mutation type ($0/0 \rightarrow \text {1/-}$) refers to the case when one allele is deleted, and the other is mutated from the wildtype, while the coincident deletion and back mutation ($1/1 \rightarrow \text {0/-}$ and $1/1^{\prime } \rightarrow \text {0/-}$) happens when one allele is deleted, and the other is mutated back to the reference nucleotide. The single deletion mutation addition ($\text {0/-} \rightarrow \text {1/-}$) takes place when the only allele of the reference-remaining single deletion genotype is mutated to an alternative nucleotide, while the single deletion back mutation addition happens when the mutated allele of the alternative-remaining single deletion genotype is mutated back to the reference nucleotide. The single deletion addition ($\text {0/-} \rightarrow \text {-}$ and $\text {1/-} \rightarrow \text {-}$) refers to the case when the only allele is deleted of the reference- and alternative-remaining single deletion genotypes. Finally, for the coincident double deletions ($0/0 \rightarrow \text {-}$, $0/1 \rightarrow \text {-}$, $1/1 \rightarrow \text {-}$, and $1/1^{\prime } \rightarrow \text {-}$), both of the alleles existing before are deleted.

Further information on the DelSIEVE model and the simulation procedure is available in Additional file 1: Supplementary notes, with supporting references [51–66].

## Supplementary information

Additional file 1: Supplementary notes.

Additional file 2: Supplementary figures S1-S33.

Additional file 3: Supplementary tables S1-S4.

## Data Availability

We analyzed three published single-cell datasets. Raw sequencing data for these datasets are available from the BioProject (https://www.ncbi.nlm.nih.gov/bioproject) database under accession code PRJNA896550 (CRC28 [32, 67]), as well as from the Sequence Read Archive (SRA, https://www.ncbi.nlm.nih.gov/sra) database under accession codes SRA053195 (TNBC16 [35, 68]) and SRP067815 (CRC48 [40, 69]). DelSIEVE is implemented in Java and is accessible at https://github.com/szczurek-lab/DelSIEVE [70]. The simulator is hosted at https://github.com/szczureklab/DelSIEVE_simulator [71], and the reproducible benchmarking framework is available at https://github.com/szczurek-lab/DelSIEVE_benchmark_pipeline [72]. The scripts for generating all figures in this paper are hosted at https://github.com/szczurek-lab/DelSIEVE_analysis [73]. All aforementioned code are freely accessible under a GNU General Public License v3.0 license. The source code used in the manuscript is available on Zenodo [74–77]
