# Supplementary material for: DelSIEVE: cell phylogeny modeling of single nucleotide variants and deletions from single-cell DNA sequencing data
Source: Genome Biol. 2025 Aug 25;26:255. doi: 10.1186/s13059-025-03738-9 (PMC12376439; doi:10.1186/s13059-025-03738-9)
Supplement: Supplementary file 1 — Additional file 1: Supplementary notes. [file 13059_2025_3738_MOESM1_ESM.pdf]

DelSIEVE: cell phylogeny modeling of single nucleotide variants and deletions from single-cell DNA sequencing data

Supplementary notes

Senbai Kang[1], Nico Borgsmüller[2,3], Monica Valecha[4,5], Magda Markowska[1,6], Jack Kuipers[2,3], Niko Beerenwinkel[2,3], David Posada[4,5,7], and Ewa Szczurek[8,1*]

[1] *Faculty of Mathematics, Informatics and Mechanics, University of Warsaw, Warsaw, Poland*

[2] *Department of Biosystems Science and Engineering, ETH Zurich, 4058 Basel, Switzerland*

[3] *SIB Swiss Institute of Bioinformatics, 4058 Basel, Switzerland*

[4] *CINBIO, Universidade de Vigo, 36310 Vigo, Spain*

[5] *Galicia Sur Health Research Institute (IIS Galicia Sur), SERGAS-UVIGO*

[6] *Medical University of Warsaw, Postgraduate School of Molecular Medicine, Warsaw, Poland*

[7] *Department of Biochemistry, Genetics, and Immunology, Universidade de Vigo, 36310 Vigo, Spain*

[8] *Institute of AI for Health, Helmholtz Zentrum München, German Research Center for Environmental Health, Neuherberg, Germany*

[*] *Correspondence: ewa.szczurek@helmholtz-munich.de*

DelSIEVE model: continued

To model ADO mode using DelSIEVE, we introduce the corresponding prior distribution of α_{ij}, $P(\alpha_{ij} \,|\, g_{ij}, \theta_A)$, as defined in Additional file 3: Table S1, where θ_A denotes the allelic dropout rate under the ADO mode. One should consider the "ADO occurred" column as the value of an additional hidden random variable indicating the occurrence of an ADO, which will be marginalized out in the model. For example, the probability of an ADO when $g_{ij} = 0/\text{-}$ equals ${}^{\theta_A}\!/_2$, because there is only one allele left to be dropped out. For genotype -, it is certain that ADO has not occurred as there is no allele existing.

To generalize DelSIEVE to model both ADO and LDO, we allow more than one allele to drop out. $P(\alpha_{ij} \,|\, g_{ij}, \theta_L)$ is defined in Additional file 3: Table S2, where θ_L represents the allelic dropout rate under the LDO mode. We assume that the ADOs occur to each allele independently. For instance, when $g_{ij} = 0/0$, the probability of $\alpha_{ij} = 0$ is θ_L^2, happening only when both alleles drop out. For genotype 0/-, the sole allele drops out with probability θ_L, resulting in zero sequenced alleles.

DelSIEVE likelihood

Combining the statistical phylogenetic model and the model of raw read counts described above, the likelihood of the observed read count data under the DelSIEVE model becomes

$$P\left(\mathcal{D}^{(1)}, \mathcal{D}^{(2)} \,\middle|\, \mathcal{T}, \boldsymbol{\beta}, Q, h, \eta, t, v, \theta, f, w_1, w_2\right). \tag{S.1}$$

To simplify notation, we denote some variables in the statistical phylogenetic model as $\Theta = \{\mathcal{T}, \boldsymbol{\beta}, Q, h, \eta\}$ and some in the model of raw read counts as $\Phi = \{t, v, \theta, f, w_1, w_2\}$. By taking the logarithm, Equation (S.1) becomes

$$\log \mathcal{L}(\Theta, \Phi) = \log \mathcal{L}^{(1)}(\Theta, \Phi) + \log \mathcal{L}^{(2)}(\Theta, \Phi), \tag{S.2}$$

where $\mathcal{L}^{(1)}$ is the tree likelihood corrected for acquisition bias computed for candidate SNV sites in $\mathcal{D}^{(1)}$, while $\mathcal{L}^{(2)}$ is the likelihood computed for background sites in $\mathcal{D}^{(2)}$, referred to as the background likelihood.

Acquisition bias in the context of phylogenetic reconstruction refers to the overestimation of branch lengths (expected number of mutations per site) due to the use of only variable sites [49,

50]. Here, we correct for it following [51]:

$$\log \mathcal{L}^{(1)} = \log P\left(\mathcal{D}^{(1)} \,\middle|\, \Theta, \Phi\right) + I' \log\left(\frac{1}{I}\sum_{i=1}^{I} C_i\right), \tag{S.3}$$

where the first component is the uncorrected log-likelihood for SNV sites, and C_i in the second component is the likelihood of SNV site i being invariant (see below).

To compute $\log P\left(\mathcal{D}^{(1)} \,\middle|\, \Theta, \Phi\right)$ in Equation (S.3), we decompose it according to the probabilistic graphical model in Figure 1b. Assuming independent and identical evolution of each candidate variant site, $\log P\left(\mathcal{D}^{(1)} \,\middle|\, \Theta, \Phi\right)$ is defined as:

$$\begin{aligned}
\log P\left(\mathcal{D}^{(1)} \,\middle|\, \Theta, \Phi\right) &= \sum_{i=1}^{I} \log \sum_{\boldsymbol{g}_i^{(L)}, \boldsymbol{g}_i^{(A)} \setminus \{g_{i(2J)}\}} \left[P\left(\mathcal{D}_i^{(1)} \,\middle|\, \boldsymbol{g}_i^{(L)}, \Phi\right) \right. \\
&\qquad \left. \times P\left(\boldsymbol{g}_i^{(L)}, \boldsymbol{g}_i^{(A)} \setminus \{g_{i(2J)}\} \,\middle|\, \Theta\right) \right] \\
&= \sum_{i=1}^{I} \log \sum_{\boldsymbol{g}_i^{(L)}, \boldsymbol{g}_i^{(A)} \setminus \{g_{i(2J)}\}} \left[\prod_{j=1}^{J} P\left(\boldsymbol{m}_{ij}, c_{ij} \,|\, g_{ij}, \Phi\right) \right. \\
&\qquad \left. \times P\left(\boldsymbol{g}_i^{(L)}, \boldsymbol{g}_i^{(A)} \setminus \{g_{i(2J)}\} \,\middle|\, \Theta\right) \right] \\
&= \sum_{i=1}^{I} \sum_{j=1}^{J} \log \sum_{\boldsymbol{g}_i^{(L)}, \boldsymbol{g}_i^{(A)} \setminus \{g_{i(2J)}\}} \left[P\left(\boldsymbol{m}_{ij}, c_{ij} \,|\, g_{ij}, \Phi\right) \right. \\
&\qquad \left. \times P\left(\boldsymbol{g}_i^{(L)}, \boldsymbol{g}_i^{(A)} \setminus \{g_{i(2J)}\} \,\middle|\, \Theta\right) \right],
\end{aligned} \tag{S.4}$$

where $P(\boldsymbol{m}_{ij}, c_{ij} \,|\, g_{ij}, \Phi)$, representing the model of raw read counts, is similarly decomposed into

$$\begin{aligned}
P\left(\boldsymbol{m}_{ij}, c_{ij} \,|\, g_{ij}, \Phi\right) &= P\left(\boldsymbol{m}_{ij}, c_{ij} \,|\, g_{ij}, f, w_{ij}, t, v, \theta\right) \\
&= \sum_{\alpha_{ij}, g'_{ij}} P\left(\boldsymbol{m}_{ij}, c_{ij}, \alpha_{ij}, g'_{ij} \,|\, g_{ij}, f, w_{ij}, t, v, \theta\right) \\
&= \sum_{\alpha_{ij}, g'_{ij}} \left[P\left(\boldsymbol{m}_{ij} \,|\, c_{ij}, g'_{ij}, f, w_{ij}\right) P\left(g'_{ij} \,|\, g_{ij}, \alpha_{ij}\right) \right. \\
&\qquad \left. \times P\left(c_{ij} \,|\, \alpha_{ij}, t, v\right) P\left(\alpha_{ij} \,|\, g_{ij}, \theta\right) \right].
\end{aligned} \tag{S.5}$$

$P(c_{ij} \,|\, \alpha_{ij}, t, v)$ in Equation (S.5) is defined through Equations (3)-(5), and $P(\boldsymbol{m}_{ij} \,|\, c_{ij}, g'_{ij}, f, w_{ij})$ is defined in Equation (11). Under the ADO mode, $P(\alpha_{ij} \,|\, g_{ij}, \theta)$ and $P(g'_{ij} \,|\, g_{ij}, \alpha_{ij})$ are defined as shown in Additional file 3: Table S1 and Table 1, respectively, while under the LDO mode in Additional file 3: Table S2 and Table 2, respectively. As a result, Equation (S.5) takes distinct

forms under different dropout modes.

For the ADO mode, Equation (S.5) is further represented as

$$P\left(\boldsymbol{m}_{ij}, c_{ij} \,|\, g_{ij}, \Phi\right) = \begin{cases} P_{0/0} \cdot P(c_{ij} \,|\, \alpha_{ij} = 2, t, v) \cdot (1-\theta) \\ \qquad + P_{0/\text{-}} \cdot P(c_{ij} \,|\, \alpha_{ij} = 1, t, v) \cdot \theta, \text{ if } g_{ij} = 0/0, \\ P_{0/1} \cdot P(c_{ij} \,|\, \alpha_{ij} = 2, t, v) \cdot (1-\theta) \\ \qquad + \dfrac{1}{2}(P_{0/\text{-}} + P_{1/\text{-}}) \cdot P(c_{ij}|\alpha_{ij} = 1, t, v) \cdot \theta, \text{ if } g_{ij} = 0/1, \\ P_{1/1} \cdot P(c_{ij} \,|\, \alpha_{ij} = 2, t, v) \cdot (1-\theta) \\ \qquad + P_{1/\text{-}} \cdot P(c_{ij} \,|\, \alpha_{ij} = 1, t, v) \cdot \theta, \text{ if } g_{ij} = 1/1, \\ P_{1/1'} \cdot P(c_{ij} \,|\, \alpha_{ij} = 2, t, v) \cdot (1-\theta) \\ \qquad + P_{1/\text{-}} \cdot P(c_{ij} \,|\, \alpha_{ij} = 1, t, v) \cdot \theta, \text{ if } g_{ij} = 1/1', \\ P_{0/\text{-}} \cdot P(c_{ij} \,|\, \alpha_{ij} = 1, t, v) \cdot (1 - \dfrac{\theta}{2}) \\ \qquad + P_{\text{-}} \cdot P(c_{ij} \,|\, \alpha_{ij} = 0, t, v) \cdot \dfrac{\theta}{2}, \text{ if } g_{ij} = 0/\text{-}, \\ P_{1/\text{-}} \cdot P(c_{ij} \,|\, \alpha_{ij} = 1, t, v) \cdot (1 - \dfrac{\theta}{2}) \\ \qquad + P_{\text{-}} \cdot P(c_{ij} \,|\, \alpha_{ij} = 0, t, v) \cdot \dfrac{\theta}{2}, \text{ if } g_{ij} = 1/\text{-}, \\ P_{\text{-}} \cdot P(c_{ij} \,|\, \alpha_{ij} = 0, t, v), \text{ if } g_{ij} = \text{-}\,. \end{cases} \tag{S.6}$$

For the LDO mode, Equation (S.5) is

$$
P\left(\boldsymbol{m}_{ij}, c_{ij} \,|\, g_{ij}, \Phi\right) = \begin{cases}
P_{0/0} \cdot P(c_{ij} \,|\, \alpha_{ij} = 2, t, v) \cdot (1-\theta)^2 \\
\quad + P_{0/\text{-}} \cdot P(c_{ij} \,|\, \alpha_{ij} = 1, t, v) \cdot 2 \cdot \theta \cdot (1-\theta) \\
\quad + P_{\text{-}} \cdot P(c_{ij} \,|\, \alpha_{ij} = 0, t, v) \cdot \theta^2, \text{ if } g_{ij} = 0/0, \\
P_{0/1} \cdot P(c_{ij} \,|\, \alpha_{ij} = 2, t, v) \cdot (1-\theta)^2 \\
\quad + (P_{0/\text{-}} + P_{1/\text{-}}) \cdot P(c_{ij} | \alpha_{ij} = 1, t, v) \cdot \theta \cdot (1-\theta) \\
\quad + P_{\text{-}} \cdot P(c_{ij} \,|\, \alpha_{ij} = 0, t, v) \cdot \theta^2, \text{ if } g_{ij} = 0/1, \\
P_{1/1} \cdot P(c_{ij} \,|\, \alpha_{ij} = 2, t, v) \cdot (1-\theta)^2 \\
\quad + P_{1/\text{-}} \cdot P(c_{ij} \,|\, \alpha_{ij} = 1, t, v) \cdot 2 \cdot \theta \cdot (1-\theta) \\
\quad + P_{\text{-}} \cdot P(c_{ij} \,|\, \alpha_{ij} = 0, t, v) \cdot \theta^2, \text{ if } g_{ij} = 1/1, \\
P_{1/1'} \cdot P(c_{ij} \,|\, \alpha_{ij} = 2, t, v) \cdot (1-\theta)^2 \\
\quad + P_{1/\text{-}} \cdot P(c_{ij} \,|\, \alpha_{ij} = 1, t, v) \cdot 2 \cdot \theta \cdot (1-\theta) \\
\quad + P_{\text{-}} \cdot P(c_{ij} \,|\, \alpha_{ij} = 0, t, v) \cdot \theta^2, \text{ if } g_{ij} = 1/1', \\
P_{0/\text{-}} \cdot P(c_{ij} \,|\, \alpha_{ij} = 1, t, v) \cdot (1-\theta) \\
\quad + P_{\text{-}} \cdot P(c_{ij} \,|\, \alpha_{ij} = 0, t, v) \cdot \theta, \text{ if } g_{ij} = 0/\text{-}, \\
P_{1/\text{-}} \cdot P(c_{ij} \,|\, \alpha_{ij} = 1, t, v) \cdot (1-\theta) \\
\quad + P_{\text{-}} \cdot P(c_{ij} \,|\, \alpha_{ij} = 0, t, v) \cdot \theta, \text{ if } g_{ij} = 1/\text{-}, \\
P_{\text{-}} \cdot P(c_{ij} \,|\, \alpha_{ij} = 0, t, v), \text{ if } g_{ij} = \text{-}\,.
\end{cases} \tag{S.7}
$$

Equation (S.4) is computed efficiently using Felsenstein's pruning algorithm [52]. For I candidate SNV sites, J cells and K genotype states in G (for DelSIEVE $K = 7$), the time complexity of the Felsenstein's pruning algorithm is $\mathcal{O}(IJK^2)$.

Since in the second component of Equation (S.3), C_i corresponds to the likelihood of candidate SNV site i being invariant, it is computed as the joint probability of $\mathcal{D}_i$ and $\boldsymbol{g}_i^{(L)} = 0/0$:

$$
\begin{aligned}
C_i &= P\left(\mathcal{D}_i^{(1)}, \boldsymbol{g}_i^{(L)} = 0/0 \,\middle|\, \Theta, \Phi\right) \\
&= P\left(\mathcal{D}_i^{(1)} \,\middle|\, \boldsymbol{g}_i^{(L)} = 0/0, \Phi\right) \sum_{\boldsymbol{g}_i^{(A)} \setminus \{g_{i(2J)}\}} P\left(\boldsymbol{g}_i^{(L)} = 0/0, \boldsymbol{g}_i^{(A)} \setminus \{g_{i(2J)}\} \,\middle|\, \Theta\right) \\
&= \prod_{j=1}^{J} P\left(\boldsymbol{m}_{ij}, c_{ij} \,|\, g_{ij} = 0/0, \Phi\right) \sum_{\boldsymbol{g}_i^{(A)} \setminus \{g_{i(2J)}\}} P\left(\boldsymbol{g}_i^{(L)} = 0/0, \boldsymbol{g}_i^{(A)} \setminus \{g_{i(2J)}\} \,\middle|\, \Theta\right),
\end{aligned} \tag{S.8}
$$

which is computed similarly to Equation (S.4), but with g_{ij} for $j = 1, \dots J$ fixed to 0/0. In fact, C_i and $\log P\left(\mathcal{D}_i^{(1)} \,\middle|\, \Theta, \Phi\right)$ are computed simultaneously in the implementation for optimized efficiency.

To efficiently compute $\log \mathcal{L}^{(2)}$, the background likelihood in Equation (S.2), we make several simplifications similar to SIEVE. Specifically, we assume that each cell at each background site has the wildtype genotype and is sequenced without dropouts and with at least one read per allele. We also assume that $P(c_{ij} \,|\, \alpha_{ij}, t, v) = 1$ and $P\left(\boldsymbol{g}_i^{(L)} = 0/0, \boldsymbol{g}_i^{(A)} \setminus \{g_{i(2J)}\} \,\middle|\, \Theta\right) = 1$, thereby ignoring the model of sequencing coverage and the tree log-likelihood for the background sites i for $i = 1, \dots I'$. With an alternative form of the Dirichlet-multinomial distribution, $\log \mathcal{L}^{(2)}$ is approximately and efficiently computed by

$$\begin{aligned}
\log \mathcal{L}^{(2)}(f, w_1) &= \sum_{i=1}^{I'} \sum_{j=1}^{J} \log P_{0/0} \\
&= \sum_{i=1}^{I'} \sum_{j=1}^{J} \log \left[\frac{\Gamma(w_1)\Gamma(c_{ij}+1)}{\Gamma(c_{ij}+w_1)} \prod_{k=1}^{3} \frac{\Gamma\left(m_{ijk} + \frac{1}{3} f w_1\right)}{\Gamma\left(\frac{1}{3} f w_1\right)\Gamma(m_{ijk}+1)} \right. \\
&\qquad \left. \times \frac{\Gamma\left(c_{ij} - \sum_{k=1}^{3} m_{ijk} + (1-f)w_1\right)}{\Gamma((1-f)w_1)\Gamma\left(c_{ij} - \sum_{k=1}^{3} m_{ijk} + 1\right)} \right] \\
&= I'J \left[\log\Gamma(w_1) - 3\log\Gamma\left(\frac{1}{3} f w_1\right) - \log\Gamma((1-f)w_1) \right] \\
&\quad + \sum_{c=1}^{\max(c_{ij})} N_c (\log\Gamma(c+1) - \log\Gamma(c+w_1)) \\
&\quad + \sum_{k=1}^{3} \sum_{m_k=1}^{\max(m_{ijk})} N_{m_k} \left(\log\Gamma\left(m_k + \frac{1}{3} f w_1\right) - \log\Gamma(m_k+1) \right) \\
&\quad + \sum_{c - \sum_{k=1}^{3} m_k = 1}^{\max(c_{ij} - \sum_{k=1}^{3} m_{ijk})} N_{c - \sum_{k=1}^{3} m_k} \left(\log\Gamma\left(c - \sum_{k=1}^{3} m_k + (1-f)w_1\right) \right. \\
&\qquad \left. - \log\Gamma\left(c - \sum_{k=1}^{3} m_k + 1\right) \right),
\end{aligned} \tag{S.9}$$

where $P_{0/0}$ is defined in Equation (11). Across I' background sites and J cells, N_c, N_{m_k} for $k = 1, 2, 3$, and $N_{c - \sum_{k=1}^{3} m_k}$ represent the unique occurrences of sequencing coverage c, of alternative nucleotide read counts m_k for $k = 1, 2, 3$, and of reference nucleotide read counts $c - \sum_{k=1}^{3} m_k$, respectively. Some terms, namely $\log\Gamma(c+1)$, $-\log\Gamma(m_k+1)$ for $k = 1, 2, 3$, and $-\log\Gamma\left(c - \sum_{k=1}^{3} m_k + 1\right)$, are constants, and thus they are not updated in the MCMC iterations.

The time complexity of Equation (S.9) is $\mathcal{O}(c)$, where c is the number of unique values in the set of values representing sequencing coverage and read counts for all four nucleotides across I'

background sites and J cells. Since generally $IJK^2 \gg c$, the overall worst-case time complexity of model likelihood is $\mathcal{O}(IJK^2)$. It is worth noting that given I candidate variant sites and J cells, the time complexity of DelSIEVE is around three times greater than that of SIEVE due to the expanded genotype state space.

Priors

Similar to SIEVE, we use prior distributions predefined and implemented in BEAST 2 for some of the hidden random variables in the DelSIEVE model. For the cell phylogeny given by $\mathcal{T}$ and $\boldsymbol{\beta}$, we set a prior following the Kingman coalescent process with an exponentially growing population, denoted

$$P(\mathcal{T}, \boldsymbol{\beta} \mid M, e), \tag{S.10}$$

where M and e are hidden random variables, representing the scaled population size and the exponential growth rate, respectively. The analytical form of Equation (S.10) is defined at length in [53].

The default assumed distribution for M in BEAST 2 is

$$P(M \mid \delta) = \frac{1}{\delta}, \tag{S.11}$$

where δ is the current proposed value of M.

As for e, the default prior is

$$e \mid \lambda, \epsilon \sim \text{Laplace}(\lambda, \epsilon), \tag{S.12}$$

where the default values of the fixed parameters are mean $\lambda = 10^{-3}$ and scale $\epsilon = 30.7$.

For η in Equation (1), an exponential prior distribution is chosen:

$$\eta \mid \gamma \sim \exp(\gamma), \tag{S.13}$$

where $\gamma = 1$.

For the relative deletion rate d, a uniform prior distribution is used:

$$d \mid \varphi \sim \text{Uniform}(0, \varphi), \tag{S.14}$$

where $\varphi = 1$.

For the hidden random variables in the model of sequencing coverage in Equations (3)-(5), a non-informative prior is set for t:

$$t \,|\, \rho \;\sim\; \text{Uniform}(0, \rho), \tag{S.15}$$

where $\rho = 1000$, while the prior for v is

$$v \,|\, \zeta \;\sim\; \exp(\zeta), \tag{S.16}$$

where $\zeta = 25$.

For the allelic dropout rate θ_A defined under the ADO (Additional file 3: Table S1) or θ_L under the LDO mode (Additional file 3: Table S2), we use a non-informative prior:

$$\theta \,|\, u \;\sim\; \text{Uniform}(0, u), \tag{S.17}$$

where $u = 1$.

Regarding the hidden random variables in the model of nucleotide read counts in Equations (7), (9) and (10), an exponential prior is set for f:

$$f \,|\, \tau \;\sim\; \exp(\tau), \tag{S.18}$$

where $\tau = 0.025$, and a log normal prior for both w_1 and w_2:

$$\begin{aligned} w_1 \,|\, \xi_1, \psi_1 \;&\sim\; \text{Log-Normal}(\xi_1, \psi_1), \\ w_2 \,|\, \xi_2, \psi_2 \;&\sim\; \text{Log-Normal}(\xi_2, \psi_2), \end{aligned} \tag{S.19}$$

where we choose for w_1 the log-transformed mean $\xi_1 = 3.9$ (150 for untransformed) and the standard deviation $\psi_1 = 1.5$, and for w_2 the log-transformed mean $\xi_2 = 0.9$ (10 for untransformed) and the standard deviation $\psi_2 = 1.7$. The mean is log-transformed using

$$\xi_{\text{transformed}} = \log(\xi_{\text{untransformed}}) - \frac{\psi^2}{2}.$$

These values of the fixed parameters in Equation (S.19) are chosen to cover a wide range of possible values for w_1 and w_2.

Posterior and MCMC

The posterior distribution of the hidden random variables is:

$$\begin{aligned}
&P\left(\mathcal{T},\boldsymbol{\beta},M,e,\eta,d,t,v,\theta,f,w_1,w_2 \,\middle|\, \mathcal{D}^{(1)},\mathcal{D}^{(2)}\right)\\
=&\frac{1}{Z}P\left(\mathcal{D}^{(1)},\mathcal{D}^{(2)} \,\middle|\, \mathcal{T},\boldsymbol{\beta},Q,\eta,t,v,\theta,f,w_1,w_2\right)\\
&\times P(\mathcal{T},\boldsymbol{\beta}\,|\,M,e)P(M\,|\,\delta)P(e\,|\,\lambda,\epsilon)\\
&\times P(\eta\,|\,\gamma)P(Q\,|\,d)P(d\,|\,\varphi)\\
&\times P(t\,|\,\rho)P(v\,|\,\zeta)P(\theta\,|\,u)P(f\,|\,\tau)\\
&\times P(w_1\,|\,\xi_1,\psi_1)P(w_2\,|\,\xi_2,\psi_2),
\end{aligned} \tag{S.20}$$

where $Z = P(\mathcal{D}^{(1)},\mathcal{D}^{(2)})$ is a normalization constant, and the likelihood of the model and priors for hidden random variables are defined in Section DelSIEVE likelihood and Section Priors, respectively. To simplify the notation, we denote the hidden random variables in Equation (S.20) as $\Lambda = \{\mathcal{T},\boldsymbol{\beta},M,e,\eta,d,t,v,\theta,f,w_1,w_2\}$.

Since Z in Equation (S.20) is intractable to calculate, we leverage BEAST2's MCMC algorithm with Metropolis-Hastings kernel to sample from the posterior distribution. In this algorithm, a new state of the hidden random variables Λ^* is proposed based on its current state Λ following a proposal distribution $q(\Lambda^*\,|\,\Lambda)$. $q(\Lambda^*\,|\,\Lambda)$ is designed to ensure the reversibility and ergodicity of the underlying Markov chain. For DelSIEVE, in each iteration, a new state of a randomly selected hidden variable is accepted with probability

$$\min\left\{1, \frac{P\left(\Lambda^*\,\middle|\,\mathcal{D}^{(1)},\mathcal{D}^{(2)}\right)q(\Lambda\,|\,\Lambda^*)}{P\left(\Lambda\,\middle|\,\mathcal{D}^{(1)},\mathcal{D}^{(2)}\right)q(\Lambda^*\,|\,\Lambda)}\right\}. \tag{S.21}$$

We employ exactly the same proposal distributions as in SIEVE. Briefly, regarding the branch lengths of the tree, the heights of the internal nodes are adjusted. For the tree topology, we use multiple moves, including subtree swapping, Wilson-Balding, and subtree sliding, where the last two moves also change branch lengths as a side effect. With respect to unknown parameters, scaling and random Gaussian walks are used. For detailed description of the aforementioned moves, refer to Drummond *et al.* [53] and Kang *et al.* [32].

To achieve more accurate parameter and tree estimates, DelSIEVE employs a two-stage sampling strategy similar to SIEVE.

Variant calling, dropout calling, and maximum likelihood gene annotation

In the computation of model likelihood using Equations (S.4) and (S.5), we marginalize out some hidden random variables: $\boldsymbol{g}_i^{(L)}$, $\boldsymbol{g}_i^{(A)}$, g'_{ij} and α_{ij}. Hence, the direct results from the MCMC sampling process are the posterior distributions of the cell phylogeny and other unknown hidden random variables. We obtain the estimates of those marginalized hidden random variables in a post-processing step. Specifically, we use the max-sum algorithm [54], by fixing the maximum clade credibility tree [55] and parameters estimated from the MCMC posterior samples using TreeAnnotator and Tracer [56], respectively. As a result, genotypes, dropout states, as well as the locations of mutated genes on the inferred cell phylogeny are determined by identifying the maximum likelihood states of $\boldsymbol{g}_i^{(L)}$, g'_{ij} and α_{ij}, as well as $\boldsymbol{g}_i^{(A)}$, respectively.

ScDNA-seq data simulator

We generated simulated data by modifying the simulator we had used in Kang *et al.* [32]. The simulator builds upon a previous simulation method, Cellcoal [57]. The first change we made was to expand the rate matrix, according to which each genomic site evolved along the tree (Additional file 3: Table S3) . The rate matrix contains 14 genotypes encoded with nucleotides, allowing for point mutations, back point mutations, and deletions. It has one parameter, the deletion rate, which is measured relatively to the mutation rate. Another change was that we implemented the LDO mode to allow more than one dropout to occur at each site for each cell. The simulator takes the same input configuration as the simulator in Kang *et al.* [32] does.

The simulation process was similar to that in Kang *et al.* [32]. Briefly, with a given number of cells, a binary cell lineage tree was first simulated following a neutral coalescent process with exponential growth under a strict molecular clock, where the mutation rate is constant. For a given number of genomic sites, each site was initialized by randomly selecting one of four nucleotides to have a reference genotype. Next, with a given mutation rate and a relative deletion rate, each site was evolved independently along the tree following the rate matrix defined in Additional file 3: Table S3. A genomic site was considered as a true SNV site if at least one cell had a genotype that was not wildtype. Dropouts were then added on top of the simulated genotypes under either ADO or LDO mode, as long as there were non-deleted alleles. We recorded the true dropout states for all cells at the true SNV sites. Size factors in Equation (6) were generated from a normal distribution with the mean = 1.2 and the variance = 0.2. The sequencing coverage was simulated using a negative binomial distribution following Equations (3)-(5),

which closely followed our model assumptions. In order to break such association between model assumption and data simulation, we made it optional to generate sequencing coverages following the procedure originally implemented in CellCoal. The read counts of each nucleotide were then generated following a multinomial distribution.

Simulation design

We designed a series of simulations to benchmark the performance of DelSIEVE. We reused and modified the benchmarking framework we used for SIEVE.

Simulations only considering SNVs

We assumed that 40 tumor cells were sampled from an exponentially growing population, whose growth rate and effective population size are 10^{-4} and 10^4, respectively. We used the same mutation rates as in the SIEVE benchmark, namely 10^{-6}, 8×10^{-6} and 3×10^{-5}. We selected two relative deletion rates: 0.1 and 0.25.

For each mutation rate, we simulated a number of genomic sites such that the preselection procedure would produce a certain amount of candidate variant sites, and additionally a high enough number of background sites so that they provide signal for branch length correction. At the same time, we kept the overall number of sites limited for computational efficiency reasons. Specifically, for a mutation rate of 10^{-6}, we evolved 10^4 genomic sites to have around $400 \sim 700$ candidate variant sites. For this simulated data, the resulting ratio of background to SNV sites was around 5. For a mutation rate of 8×10^{-6}, 10^4 genomic sites were chosen to have around 4×10^3 background sites. For mutation rate 3×10^{-5}, 1.2×10^5 genomic sites were chosen to have at least 2.5×10^3 background sites. For the higher mutation rates of 8×10^{-6} and 3×10^{-5}, the chosen numbers of genomic sites resulted in $> 5 \times 10^3$ and $> 1.1 \times 10^5$ true SNV sites, respectively. Next, the obtained genomic sites were subsetted to ensure that the number of true SNV sites in the final simulated data for different mutation rates were within the same range, and that for the higher mutation rates 8×10^{-6} and 3×10^{-5} the ratio between the number of background sites and the true SNV sites was kept similar to the data for the lowest mutation rate (at least 5). To this end, we first computed a targeted number of true SNV sites for each simulated dataset n_{target} using

$$n_{\text{target}} = \min(700, \frac{n'}{5}),$$

where n' is the number of background sites. Next, we randomly selected n_{target} sites out of the true SNV sites. Together with the n' background sites, the selected n_{target} true SNV sites formed the new simulated data.

We considered both ADO and LDO mode. The allelic dropout rate for the former was $\theta_A = 0.3$, and for the latter $\theta_L = 0.163$ (both are the default setups in Cellcoal [57]).

We had different combinations of t and v in Equations (3)-(5) for various coverage qualities. For simulated data referred to as high coverage quality, we used high mean ($t = 20$) and low variance ($v = 2$) of allelic coverage. For medium coverage quality data, we used high mean ($t = 20$) and medium variance ($v = 10$). For low coverage quality data, we fixed low mean ($t = 5$) and high variance ($v = 20$).

Other parameters were fixed when simulating the data. We set w_1 and w_2 in Equation (10) to 100 and 2.5, respectively. Moreover, we set both the amplification and sequencing error rate to 10^{-3}, and thus the effective sequencing error rate in Equation (9) was $f \approx 2 \times 10^{-3}$.

Overall, we designed 36 simulation scenarios, each repeated 10 times.

Furthermore, for each of those genotypes related to deletions, we filtered out results if the proportion of simulated ground truth was less than 0.1%. We also excluded results with a mutation rate of 10^{-6} as too few deletions were generated (less than 0.3%, 0.7% and 0.005% for alternative-left single deletion, reference-left single deletion and double deletions, respectively). For the same reason, results were also excluded for double deletions with a mutation rate of 8×10^{-6} and for double mutant genotypes with a mutation rate of 10^{-6}, both of which had less than 0.2% corresponding genotypes generated.

Simulations considering SNVs, CNAs and doublets

To simulate CNAs, we selected a specific simulation scenario described above with the following parameters: 40 cells, medium mutation rate (8×10^{-6}), high relative deletion rate 0.25, medium coverage quality ($t = 20$, $v = 10$), and single ADO mode ($\theta_A = 0.3$). We chose two levels of CNA prevalence: $^1/_3$ and $^2/_3$ of all genomic sites. CNAs were then added using the same procedure as described in Kang *et al.* [32], with the following modification regarding genotype adjustment. If a site in a cell had only one allele before simulating CNAs, i.e., the genotype was originally either 0/- (reference-remaining) or 1/- (alternative-remaining), amplifications would change the underlying genotype to either 0/0 or 1/1, and deletions would remove the only allele, resulting in double deletions genotype (-). If there were two alleles at a site in a cell, amplifications would

not change the underlying genotype, whereas deletions would accordingly.

To simulate doublets following the procedure described in the previous section, we selected two levels of doublet rates: 0.02 and 0.1.

Additionally, we considered a particularly challenging simulation scenario, where a high prevalence of CNAs ($^2/_3$ of all sites) and a high doublet rate (0.1) were added simultaneously.

Overall, we designed five simulation scenarios for CNAs and/or doublets, each repeated 10 times.

Variant calling and phylogenetic accuracy

For assessing the results of variant and dropout calling, standard performance measures such as precision, recall, F1 score, and false positive rate (FPR) were used. These measures were computed for DelSIEVE and SIEVE in the task of calling deletions as well as dropouts, and computed for Monovar in addition to DelSIEVE and SIEVE in the task of calling single and double mutant. Moverover, in order to compare to SCIPhIN, these measures were also computed for DelSIEVE, SIEVE and Monovar with respect to the occurrence of deletions and/or single and double mutant (see Section Configurations of methods in Additional file 1: Supplementary notes). Note that, for the benchmark of genotype and single ADO calling in the presence of doublets, we only considered singlets by excluding cells with doublets. The reason is that, since DelSIEVE leverages the cell phylogeny to share information across all single cells, genotype inference is implicitly interconnected among cells. Consequently, we aimed to evaluate whether the presence of doublets indirectly affected the accuracy of genotype and single ADO inference for singlets.

Both DelSIEVE and SCIPhIN identify deletions at preselected candidate sites. Hence, we subsetted the true deletions to those at the candidate variant sites when computing the metrics.

To assess the accuracy of cell phylogeny reconstruction, we used the same measurements as in Kang *et al.* [32], namely the BS distance [58] for both the tree topology and branch lengths, as well as the normalized RF distance [59] for the tree topology only (see Kang *et al.* [32]). For DelSIEVE, SIEVE and SiFit, we computed both the BS and the normalized RF distance in the rooted tree mode. For SCIPhIN, we only computed the normalized RF distance as it only infers a rooted tree without branch lengths. We used the R package phangorn to compute the BS and normalized RF distances [60].

Configurations of methods

For Monovar (commit 68fbb68), we used the true values of θ and f as priors for the false negative and false positive rates, and default values for other options.

For SCIPhIN (commit 27e5ca6), we gave it the true value of f to avoid estimating its mean error rate (option "wildMean"), and ran it with 10^6 iterations with zygosity learned (option "lz" set to 1). We also set the penalty of computing the loss (option "llp") and parallel score (option "lpp") to 30. The command line was as follows:

```
sciphin -l 1000000 --lz 1 --ll 1 --lp 1 --llp 30 --lpp 30 --ese 0 \
--wildMean 0.002
```

Note that SCIPhIN does not in its output differentiate deletions from SNVs; that is, it reports both SNVs and deletions simply as mutations.

To run SiFit (commit 9dc3774), we fed the required data with variants called by Monovar using a ternary matrix. We used the true values of θ and f as the prior for false negative rate and the estimated false positive rate, respectively. We ran it with 2×10^5 iterations.

For SIEVE, originally it only supported ADO mode. To allow comparison to DelSIEVE in LDO mode, we implemented LDO for SIEVE as well.

We enforced a strict molecular clock model for DelSIEVE and SIEVE, both of which were run for 2×10^6 and 1.5×10^6 iterations for the first and the second sampling stages, respectively. The deletion rate was inferred in the second sampling stage as it is related to the branch lengths of the cell phylogeny. Both DelSIEVE and SIEVE were configured to match the dropout mode (ADO or LDO) employed during the simulation process. Additionally, it is necessary to evaluate the performance of DelSIEVE and SIEVE when their configured dropout modes are distinct from that used to simulate a specific dataset. To this end, we selected the extremest simulation scenarios for both the dropout modes (see Section Simulation design in Additional file 1: Supplementary notes), namely the lowest and highest mutation rates (10^{-6} and 3×10^{-5}, respectively), the lowest and highest coverage qualities ($t = 20$, $v = 2$ and $t = 5$, $v = 20$, respectively), and the highest relative deletion rate (0.25).

On the real datasets, we used a log-normal relaxed molecular clock model to account for branch-wise substitution rate variation for DelSIEVE. To obtain better mixed Markov chains, we used an optimized relaxed clock model [61] rather than the default one in BEAST 2. We increased the number of iterations for both stages to 4×10^6 and 3.5×10^6, respectively. Both

the deletion rate and parameters introduced by the relaxed molecular clock model were explored in the second sampling stage. The first 10% of all samples were discarded for both sampling stages to remove samples from the burn-in phase. As the simulation results showed that the performance of DelSIEVE is not sensitive to the configured dropout mode, we applied DelSIEVE in ADO mode to the real datasets. To evaluate the convergence of the DelSIEVE model, we ran an additional MCMC chain for each real dataset using a different random seed and dispersed initial values.

To run Ginkgo on the real datasets, sample bam files were first converted to bed files using `bamtobed` function from bedtools (v2.28.0). With the bed files as input, we ran Ginkgo with default settings, where hg19 reference genome, 1,000,000 bin size and bwa were used as the aligner, and euclidian distance as the clustering option.

To run Sequenza on the real datasets, we used the `bam2seqz` command in the sequenza-utils package to convert bam files for the corresponding bulk samples to the Sequenza file format, which was subsequently binned with the seqz_binning command, using a window size of 50. With this file as input, we used the `sequenza.fit` command from Sequenza v3.0.0 to estimate the ploidy.

SNVs were annotated using Annovar (version 2020 Jun. 08) [62]. The cell phylogeny was plotted in R (version 4.2.3) [63] using ggtree [64], and the genotype heatmap was plotted using ComplexHeatmap [65]. Besides, the comparison of sequencing coverages reported by DelSIEVE and Sequenza was performed and plotted using ggstatsplot [66].

Runtime analysis

For runtime analysis, we created two additional simulation scenarios with the following common parameters: 100 cells, medium mutation rate (8×10^{-6}), high relative deletion rate (0.25), medium coverage quality ($t = 20$, $v = 10$), and single ADO mode ($\theta_A = 0.3$). In the first scenario, five replicate datasets were generated with a median number 798 of candidate variant sites, while for the second scenario, another five replicates were simulated with a median number 1585 of candidate variant sites. Both stages of DelSIEVE and SIEVE were run for 10^5 iterations under the default multithreading mode, with the same number of sites per thread.
