# Supplementary material for: DelSIEVE: cell phylogeny modeling of single nucleotide variants and deletions from single-cell DNA sequencing data
Source: Genome Biol. 2025 Aug 25;26:255. doi: 10.1186/s13059-025-03738-9 (PMC12376439; doi:10.1186/s13059-025-03738-9)
Supplement: Supplementary file 2 — Additional file 2: Supplementary figures S1-S33. [file 13059_2025_3738_MOESM2_ESM.pdf]

DelSIEVE: cell phylogeny modeling of single nucleotide variants and deletions from single-cell DNA sequencing data

Supplementary figures

Senbai Kang[1], Nico Borgsmüller[2,3], Monica Valecha[4,5], Magda Markowska[1,6], Jack Kuipers[2,3], Niko Beerenwinkel[2,3], David Posada[4,5,7], and Ewa Szczurek[8,1*]

[1] *Faculty of Mathematics, Informatics and Mechanics, University of Warsaw, Warsaw, Poland*

[2] *Department of Biosystems Science and Engineering, ETH Zurich, 4058 Basel, Switzerland*

[3] *SIB Swiss Institute of Bioinformatics, 4058 Basel, Switzerland*

[4] *CINBIO, Universidade de Vigo, 36310 Vigo, Spain*

[5] *Galicia Sur Health Research Institute (IIS Galicia Sur), SERGAS-UVIGO*

[6] *Medical University of Warsaw, Postgraduate School of Molecular Medicine, Warsaw, Poland*

[7] *Department of Biochemistry, Genetics, and Immunology, Universidade de Vigo, 36310 Vigo, Spain*

[8] *Institute of AI for Health, Helmholtz Zentrum München, German Research Center for Environmental Health, Neuherberg, Germany*

[*] *Correspondence: ewa.szczurek@helmholtz-munich.de*

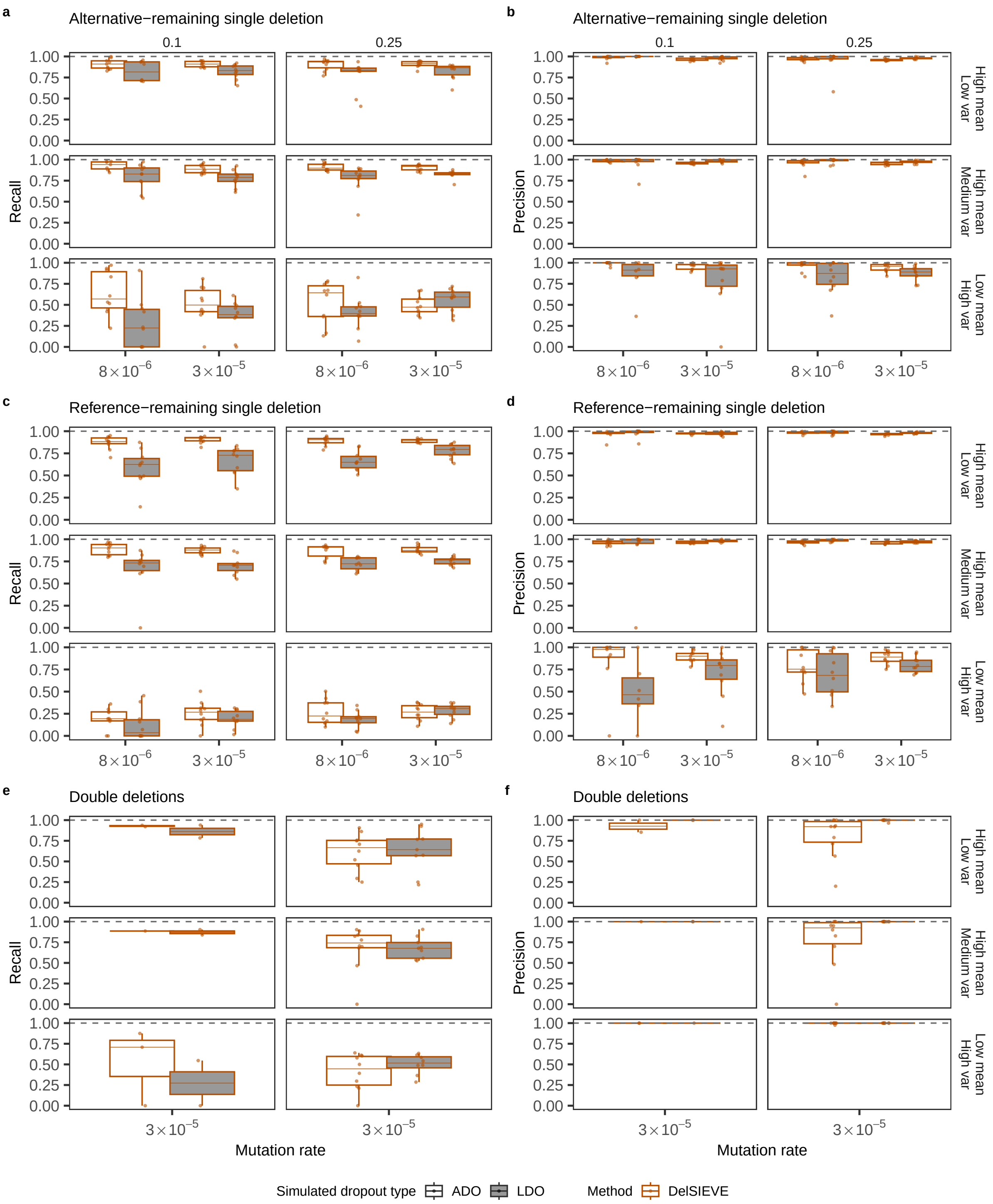

a
Alternative−remaining single deletion
b
Alternative−remaining single deletion
c
Reference−remaining single deletion
d
Reference−remaining single deletion
e
Double deletions
f
Double deletions
0.1
0.25
Recall
Precision
High mean Low var
High mean Medium var
Low mean High var
1.00
0.75
0.50
0.25
0.00
8×10−6
3×10−5
Mutation rate
Simulated dropout type
ADO
LDO
Method
DelSIEVE

Fig. S1 ***(previous page)*****: Recall and precision for the benchmark of calling deletions.** Varying are the mutation rate (the horizontal axis), the relative deletion rate (the vertical strip), the coverage quality (the horizontal strip) and the simulated dropout type (the shaded or blank boxes). Each simulation is repeated $n = 10$ times with each repetition denoted by colored dots. The gray dashed lines represent the optimal values of each metric. Box plots comprise medians, boxes covering the interquartile range (IQR), and whiskers extending to 1.5 times the IQR below and above the box. Data points were removed if the proportion of simulated ground truth was less than 0.1%. Both DelSIEVE and SIEVE were configured to match the dropout mode (ADO or LDO) employed during the simulation process. **a-b**, Box plots of the recall (**a**) and the precision (**b**) for calling alternative-left single deletion. **c-d**, Box plots of the recall (**c**) and the precision (**d**) for calling reference-left single deletion. **e-f**, Box plots of the recall (**e**) and the precision (**f**) for calling double deletions, where the results when mutation rate was 8×10^{-6} were omitted as very few double deletions were generated (less than 0.2%).

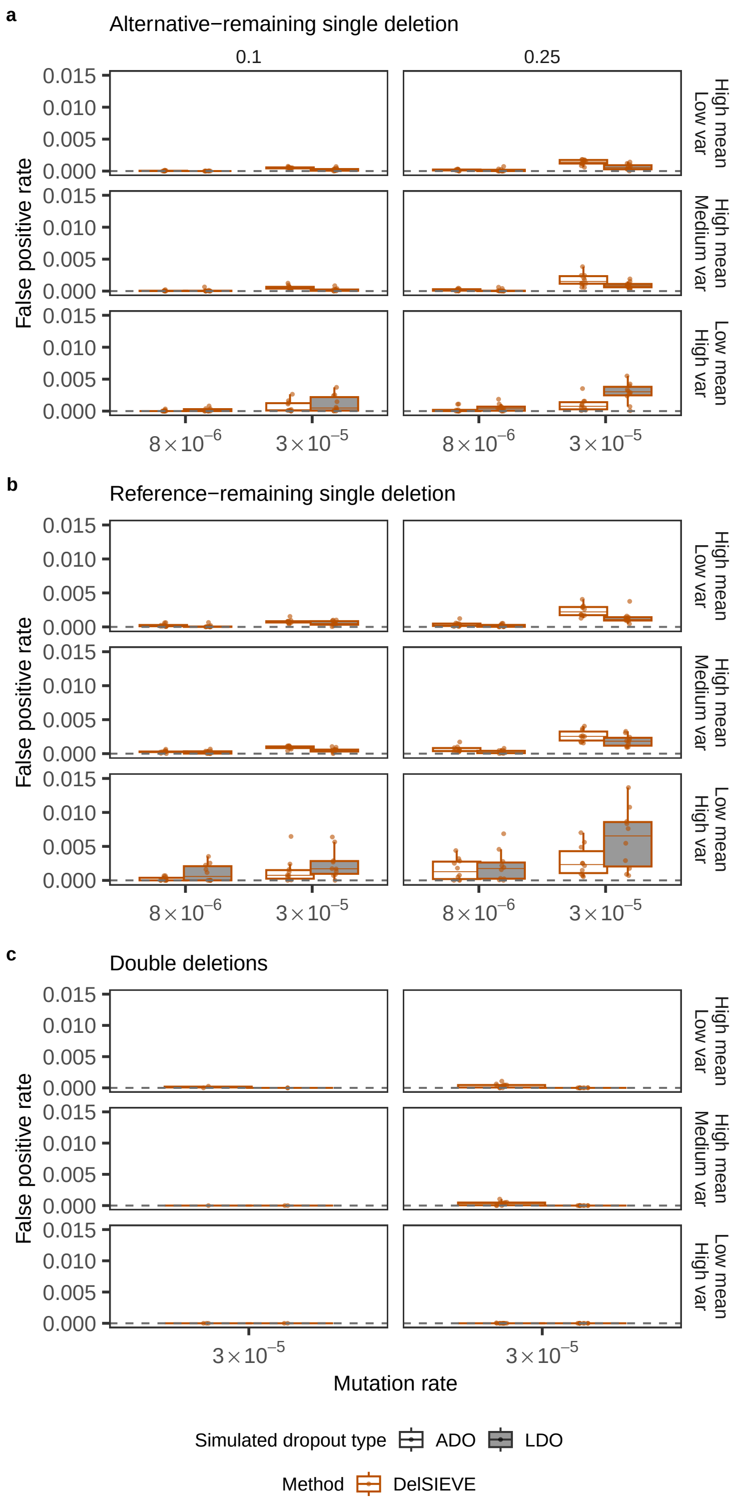
a
Alternative−remaining single deletion
0.1
0.25
False positive rate
0.015
0.010
0.005
0.000
High mean Low var
High mean Medium var
Low mean High var
8×10^−6
3×10^−5
b
Reference−remaining single deletion
c
Double deletions
Mutation rate
Simulated dropout type ADO LDO
Method DelSIEVE

Fig. S2 ***(previous page)*****: False positive rate (FPR) for the benchmark of calling deletions.** Varying are the mutation rate (the horizontal axis), the relative deletion rate (the vertical strip), the coverage quality (the horizontal strip) and the simulated dropout type (the shaded or blank boxes). Each simulation is repeated $n = 10$ times with each repetition denoted by colored dots. The gray dashed lines represent the optimal values of each metric. Box plots comprise medians, boxes covering the interquartile range (IQR), and whiskers extending to 1.5 times the IQR below and above the box. Data points were removed if the proportion of simulated ground truth was less than 0.1%. Both DelSIEVE and SIEVE were configured to match the dropout mode (ADO or LDO) employed during the simulation process. **a-c**, Box plots of the FPR for calling alternative-left single deletion (**a**), reference-left single deletion (**b**), and double deletions (**c**). The results in **c** when mutation rate was 8×10^{-6} were omitted as very few double deletions were generated (less than 0.2%).

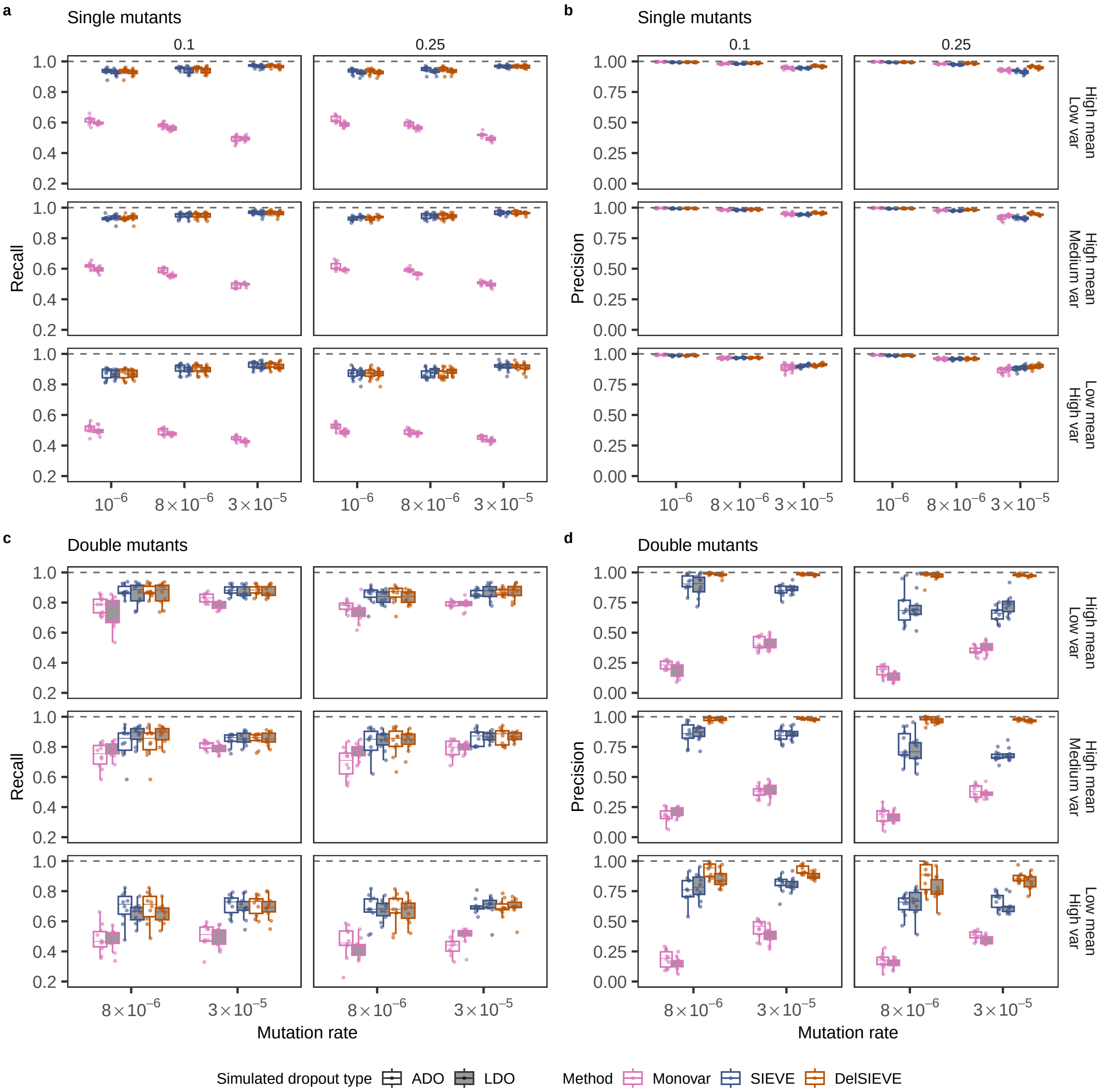

Fig. S3: Recall and precision for the benchmark of calling single and double mutant. Varying are the mutation rate (the horizontal axis), the relative deletion rate (the vertical strip), the coverage quality (the horizontal strip) and the simulated dropout type (the shaded or blank boxes). Each simulation is repeated $n = 10$ times with each repetition denoted by colored dots. The gray dashed lines represent the optimal values of each metric. Box plots comprise medians, boxes covering the interquartile range (IQR), and whiskers extending to 1.5 times the IQR below and above the box. Both DelSIEVE and SIEVE were configured to match the dropout mode (ADO or LDO) employed during the simulation process. **a-b**, Box plots of the recall (**a**) and the precision (**b**) for calling single mutant. **c-d**, Box plots of the recall (**c**) and the precision (**d**) for calling double mutant, where the results when mutation rate was 10^{-6} were omitted as very few double mutant were generated (less than 0.2%).

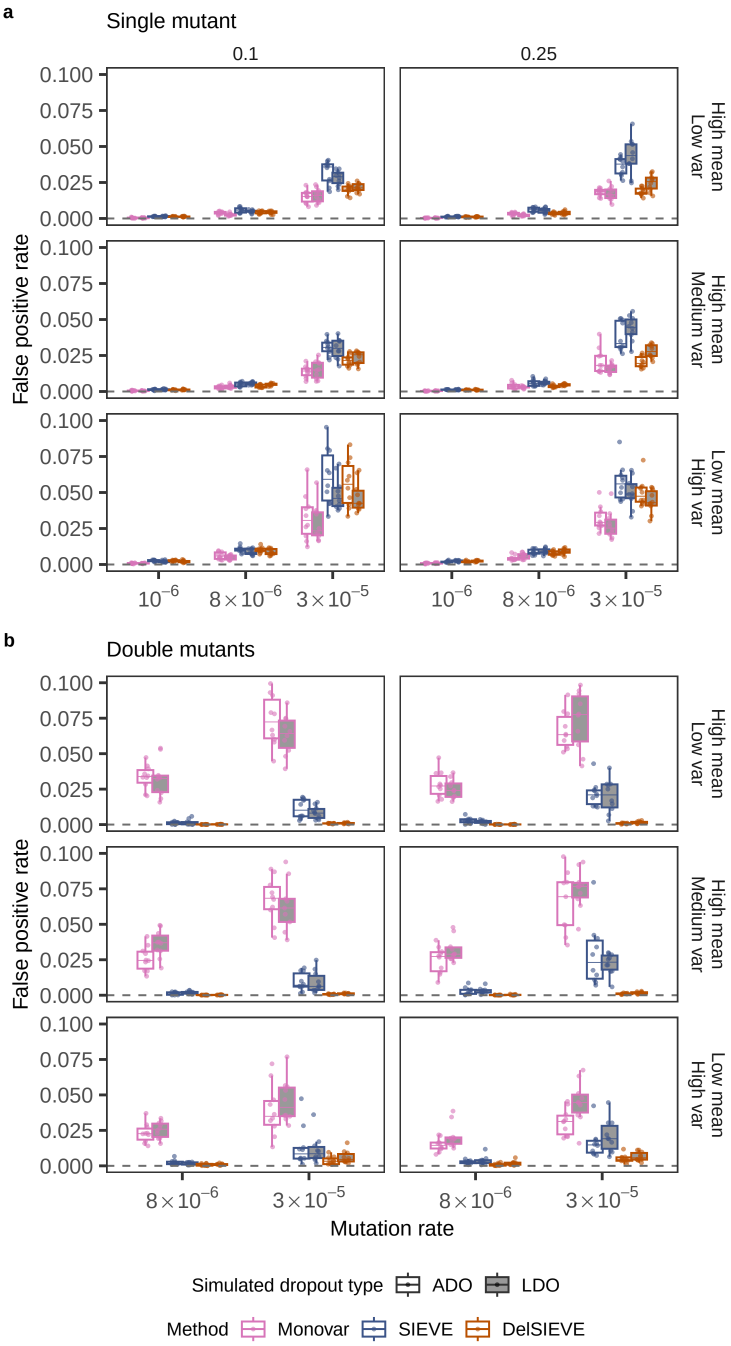

Fig. S4: False positive rate (FPR) for the benchmark of calling single and double mutant. Varying are the mutation rate (the horizontal axis), the relative deletion rate (the vertical strip), the coverage quality (the horizontal strip) and the simulated dropout type (the shaded or blank boxes). Each simulation is repeated $n = 10$ times with each repetition denoted by colored dots. The gray dashed lines represent the optimal values of each metric. Box plots comprise medians, boxes covering the interquartile range (IQR), and whiskers extending to 1.5 times the IQR below and above the box. Both DelSIEVE and SIEVE were configured to match the dropout mode (ADO or LDO) employed during the simulation process. **a-b**, Box plots of the FPR for calling single mutant (**a**) and double mutant (**b**). The results in **b** when mutation rate was 10^{-6} were omitted as very few double mutant were generated (less than 0.2%).

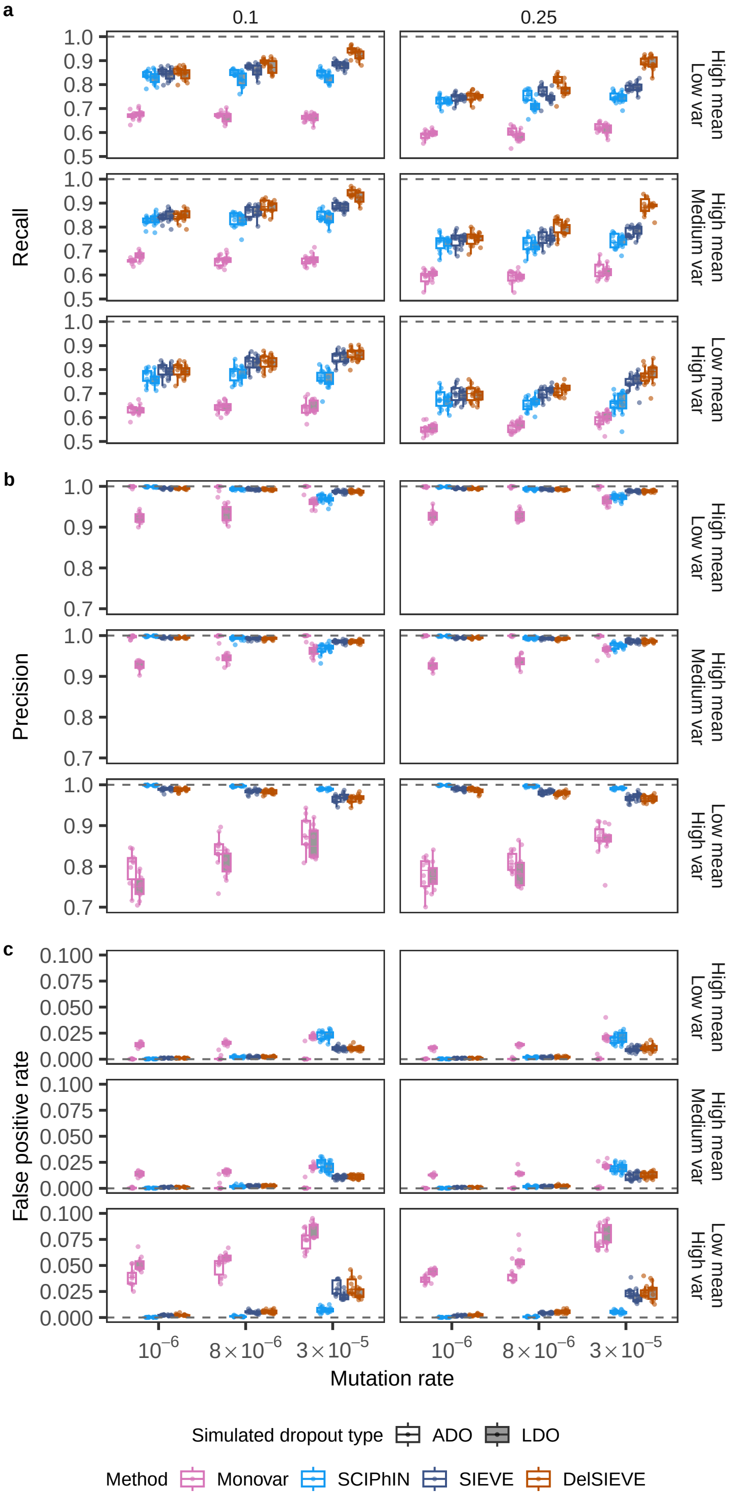

Fig. S5: Recall, precision and false positive rate (FPR) for the benchmark of the DelSIEVE model. Varying are the mutation rate (the horizontal axis), the relative deletion rate (the vertical strip), the coverage quality (the horizontal strip), and the simulated dropout type (the shaded or blank boxes). Each simulation is repeated $n = 10$ times, with each repetition denoted by colored dots. The gray dashed lines represent the optimal values of each metric. Box plots comprise medians, boxes covering the interquartile range (IQR), and whiskers extending to 1.5 times the IQR below and above the box. Data points were removed if the proportion of simulated ground truth was less than 0.1%. Both DelSIEVE and SIEVE were configured to match the dropout mode (ADO or LDO) employed during the simulation process. **a-c**, Box plots of the recall (**a**), precision (**b**) and FPR (**c**) for calling general "mutations", composed of all genotypes other than wildtype.

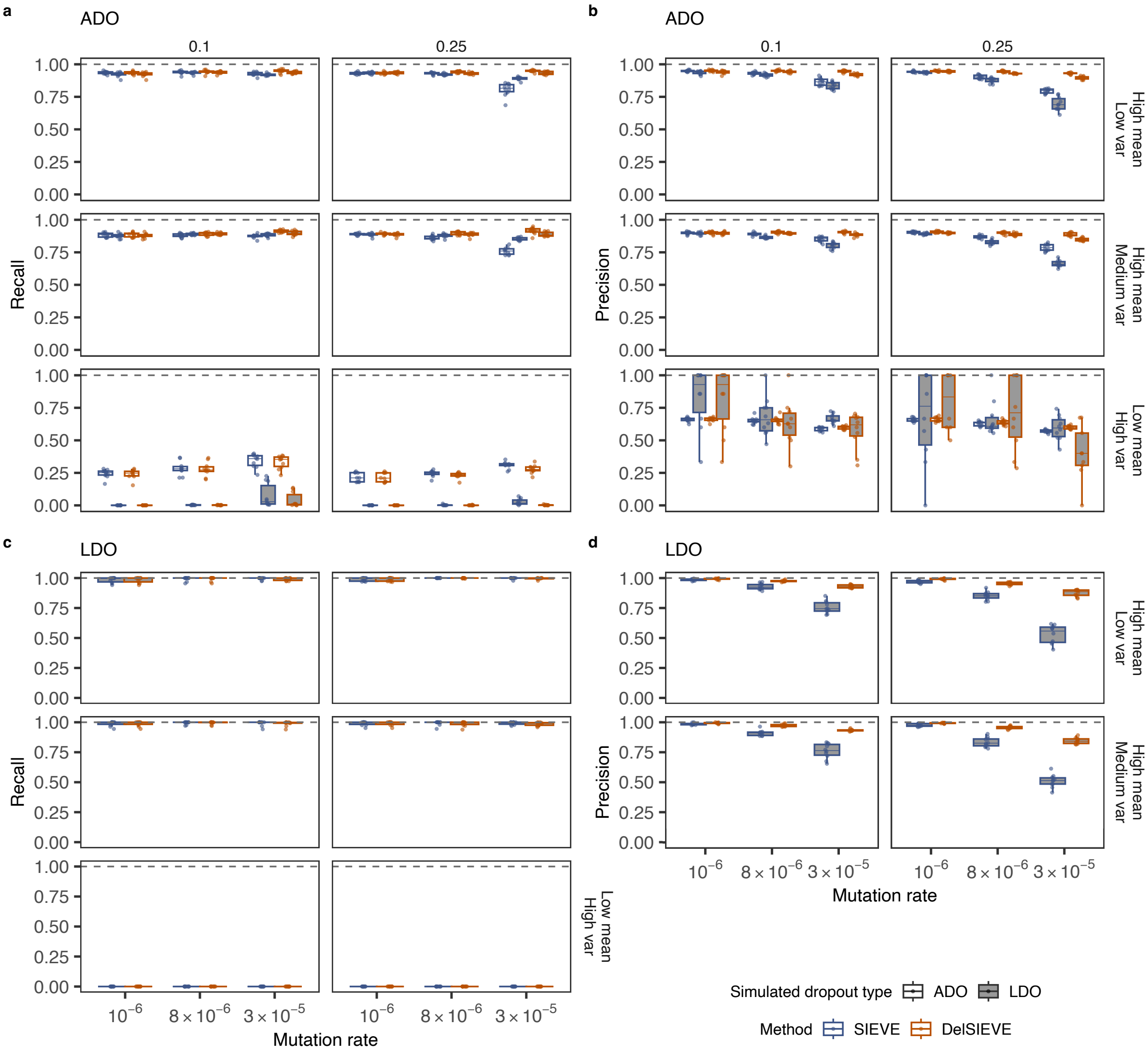

Fig. S6: Recall and precision for the benchmark of calling ADO and LDO. Varying are the mutation rate (the horizontal axis), the relative deletion rate (the vertical strip), the coverage quality (the horizontal strip) and the simulated dropout type (the shaded or blank boxes). Each simulation is repeated $n = 10$ times with each repetition denoted by colored dots. The gray dashed lines represent the optimal values of each metric. Box plots comprise medians, boxes covering the interquartile range (IQR), and whiskers extending to 1.5 times the IQR below and above the box. Both DelSIEVE and SIEVE were configured to match the dropout mode (ADO or LDO) employed during the simulation process. **a-b**, Box plots of the recall (**a**) and the precision (**b**) for calling single ADO. **c-d**, Box plots of the recall (**c**) and the precision (**d**) for calling locus dropout, where the precision were unavailable in **d** when data was of low coverage quality due to zero called locus dropout.

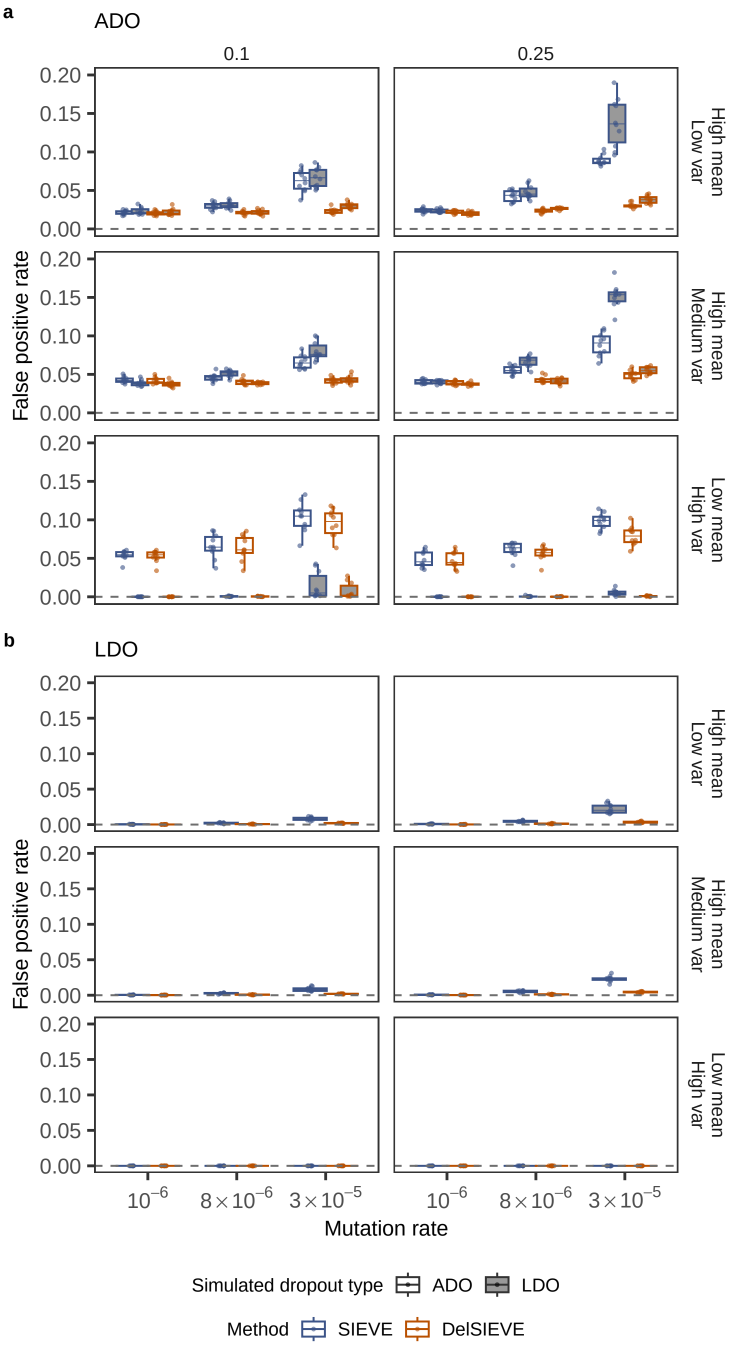

Fig. S7: False positive rate (FPR) for the benchmark of calling ADO and LDO. Varying are the mutation rate (the horizontal axis), the relative deletion rate (the vertical strip), the coverage quality (the horizontal strip) and the simulated dropout type (the shaded or blank boxes). Each simulation is repeated $n = 10$ times with each repetition denoted by colored dots. The gray dashed lines represent the optimal values of each metric. Box plots comprise medians, boxes covering the interquartile range (IQR), and whiskers extending to 1.5 times the IQR below and above the box. Both DelSIEVE and SIEVE were configured to match the dropout mode (ADO or LDO) employed during the simulation process. **a-b**, Box plots of the FPR for calling single ADO (**a**) and locus dropout (**b**).

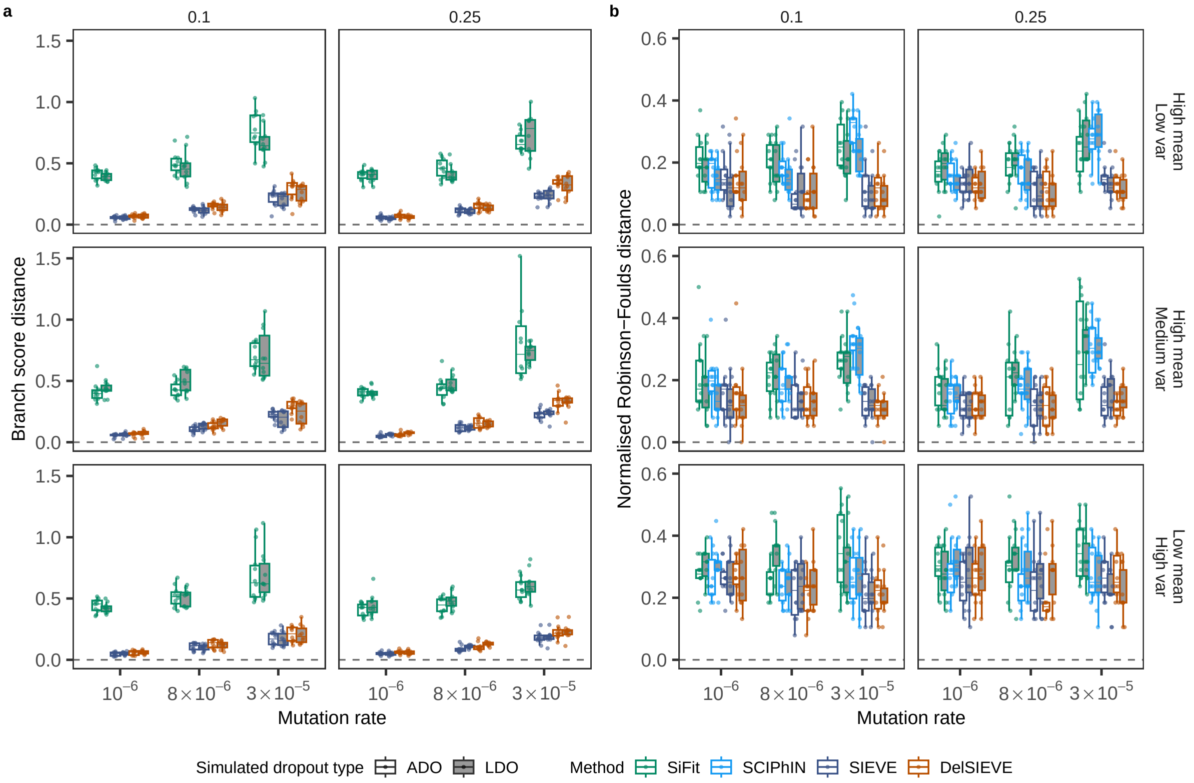

Fig. S8: Benchmark of tree inference accuracy. Varying are the mutation rate (the horizontal axis), the relative deletion rate (the vertical strip), the coverage quality (the horizontal strip) and the simulated dropout type (the shaded or blank boxes). Each simulation is repeated $n = 10$ times with each repetition denoted by colored dots. The gray dashed lines represent the optimal values of each metric. Box plots comprise medians, boxes covering the interquartile range (IQR), and whiskers extending to 1.5 times the IQR below and above the box. Both DelSIEVE and SIEVE were configured to match the dropout mode (ADO or LDO) employed during the simulation process. **a-b**, Box plots of the BS distance where the branch lengths are taken into account (**a**) and the normalized RF distance where only tree topology is considered (**b**).

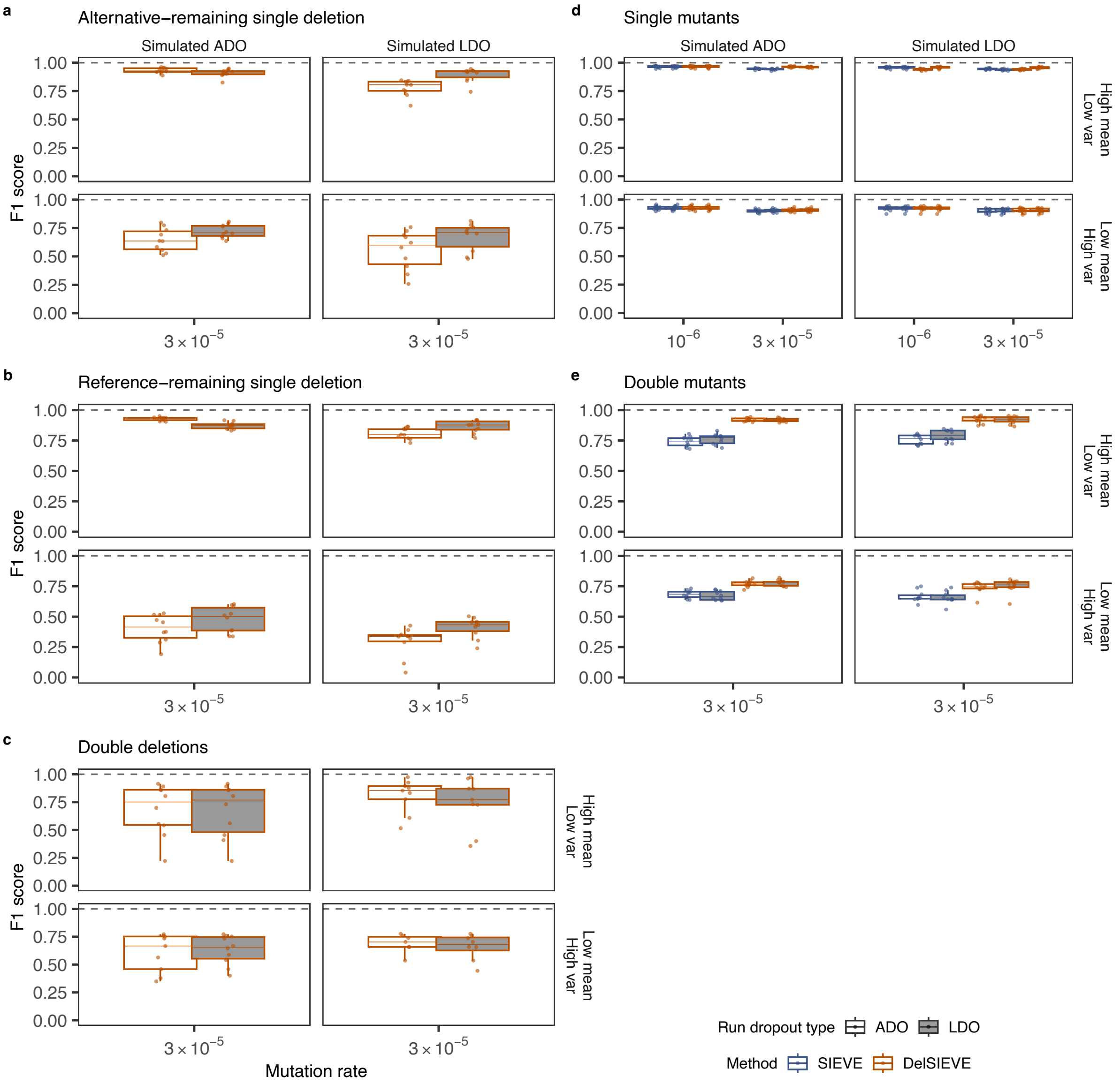

Fig. S9: F1 score for the benchmark of calling all genotypes other than wildtype. Varying are the mutation rate (the horizontal axis), the dropout type used to simulate the data (the vertical strip), the coverage quality (the horizontal strip), and the dropout type used to configure DelSIEVE and SIEVE (the shaded or blank boxes). Each simulation is repeated $n = 10$ times, with each repetition denoted by colored dots. The gray dashed lines represent the optimal values of each metric. Box plots comprise medians, boxes covering the interquartile range (IQR), and whiskers extending to 1.5 times the IQR below and above the box. Both DelSIEVE and SIEVE were run under ADO and LDO modes, regardless of that used to simulate the data. Data points were removed if the proportion of simulated ground truth was less than 0.1%. **a-e**, Box plots of the F1 score for calling alternative-left single deletion (**a**), reference-left single deletion (**b**), double deletions (**c**), single mutant (**d**), and double mutant (**e**).

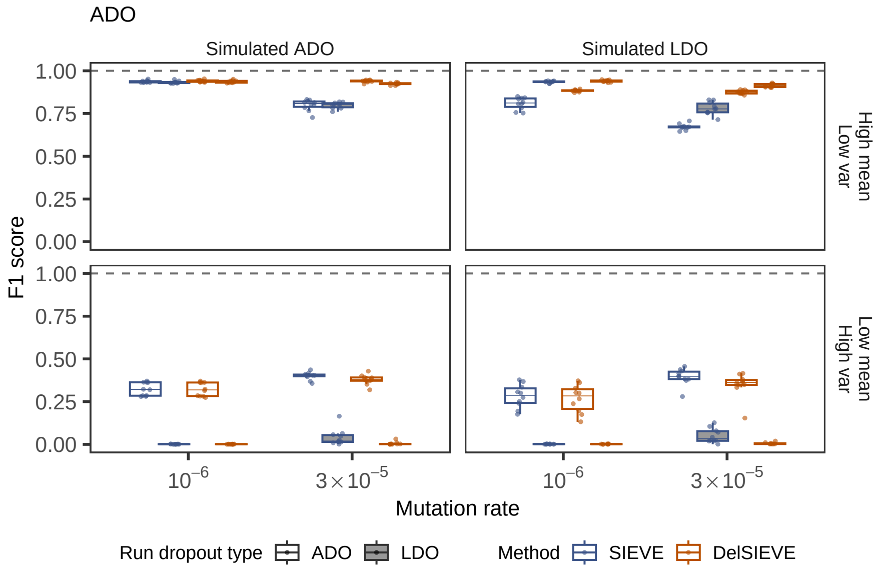

Fig. S10: F1 score for the benchmark of calling ADO. Varying are the mutation rate (the horizontal axis), the dropout type used to simulate the data (the vertical strip), the coverage quality (the horizontal strip), and the dropout type used to configure DelSIEVE and SIEVE (the shaded or blank boxes). Each simulation is repeated $n = 10$ times, with each repetition denoted by colored dots. The gray dashed lines represent the optimal values of each metric. Box plots comprise medians, boxes covering the interquartile range (IQR), and whiskers extending to 1.5 times the IQR below and above the box. Both DelSIEVE and SIEVE were run under ADO and LDO mode, regardless of that used to simulate the data.

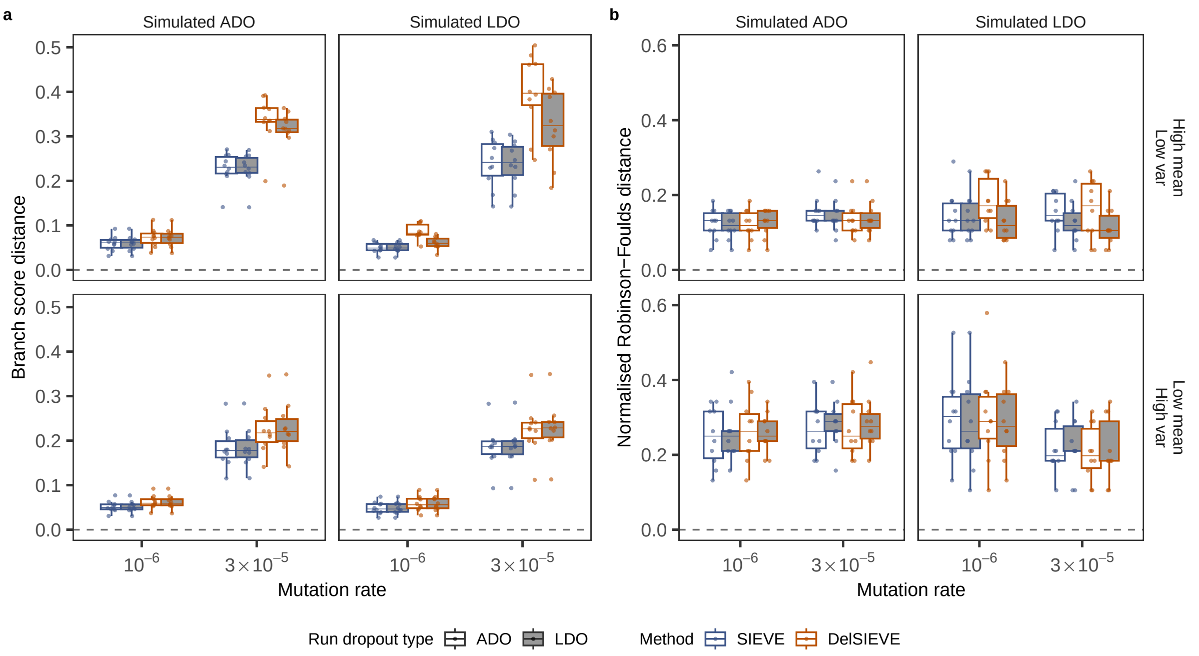

Fig. S11: Benchmark of tree inference accuracy. Varying are the mutation rate (the horizontal axis), the dropout type used to simulate the data (the vertical strip), the coverage quality (the horizontal strip), and the dropout type used to configure DelSIEVE and SIEVE (the shaded or blank boxes). Each simulation is repeated $n = 10$ times, with each repetition denoted by colored dots. The gray dashed lines represent the optimal values of each metric. Box plots comprise medians, boxes covering the interquartile range (IQR), and whiskers extending to 1.5 times the IQR below and above the box. Both DelSIEVE and SIEVE were run under ADO and LDO mode, regardless of that used to simulate the data. **a-b**, Box plots of the BS distance where the branch lengths are taken into account (**a**) and the normalized RF distance where only tree topology is considered (**b**).

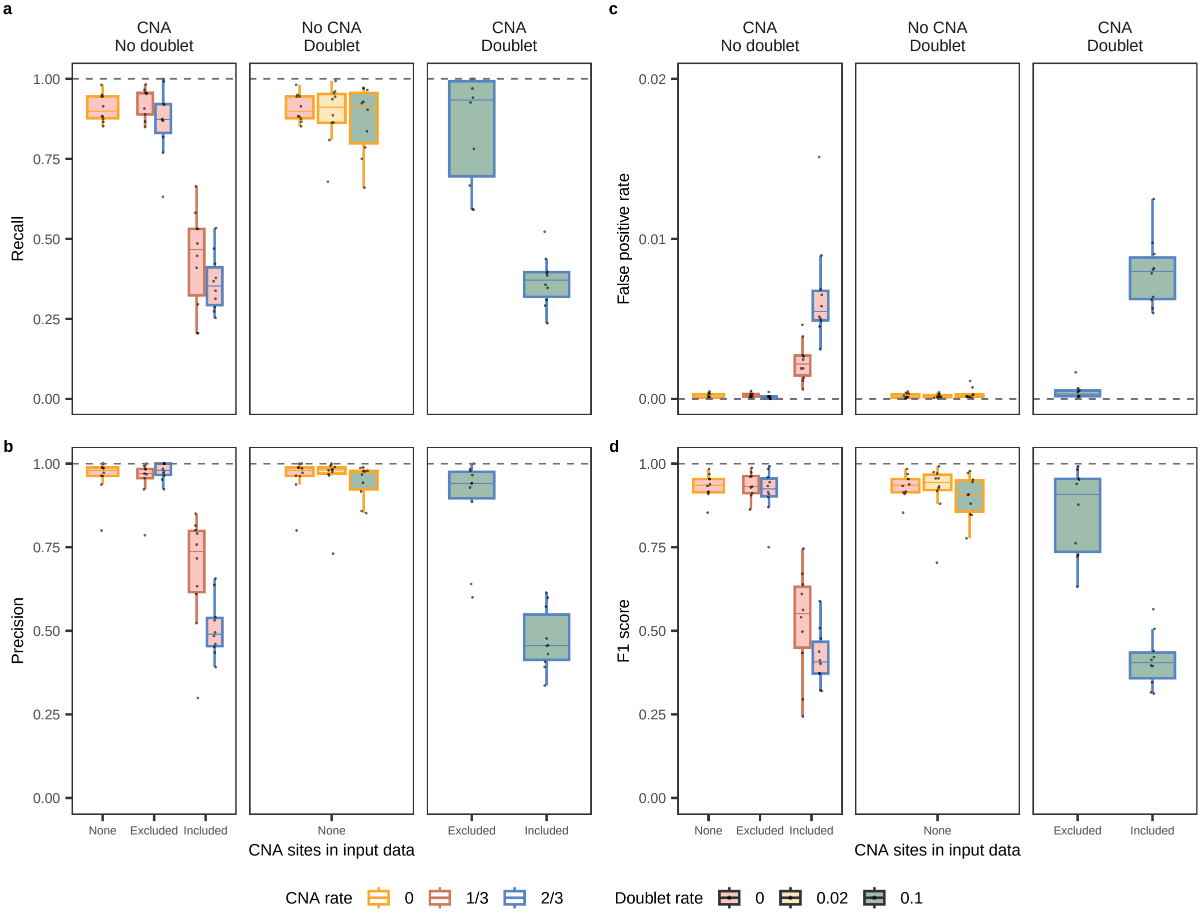

Fig. S12: Alternative-remaining genotype calling results of the DelSIEVE model considering CNAs and/or doublets. Varying are the status of CNA sites in input data (the horizontal axis), the CNA rate (the boxes with colored frames), the doublet rate (the boxes filled with colors), as well as the simulation scenarios of CNAs and doublets (the vertical strip). Each simulation is repeated $n = 10$ times, with each repetition denoted by a black dot. The gray dashed lines represent the optimal values of each metric. Box plots comprise medians, boxes covering the interquartile range (IQR), and whiskers extending to 1.5 times the IQR below and above the box. **a-d**, Box plots of the alternative-remaining genotype calling results measured by recall (**a**), precision (**b**), false positive rate (**c**) and F1 score (**d**).

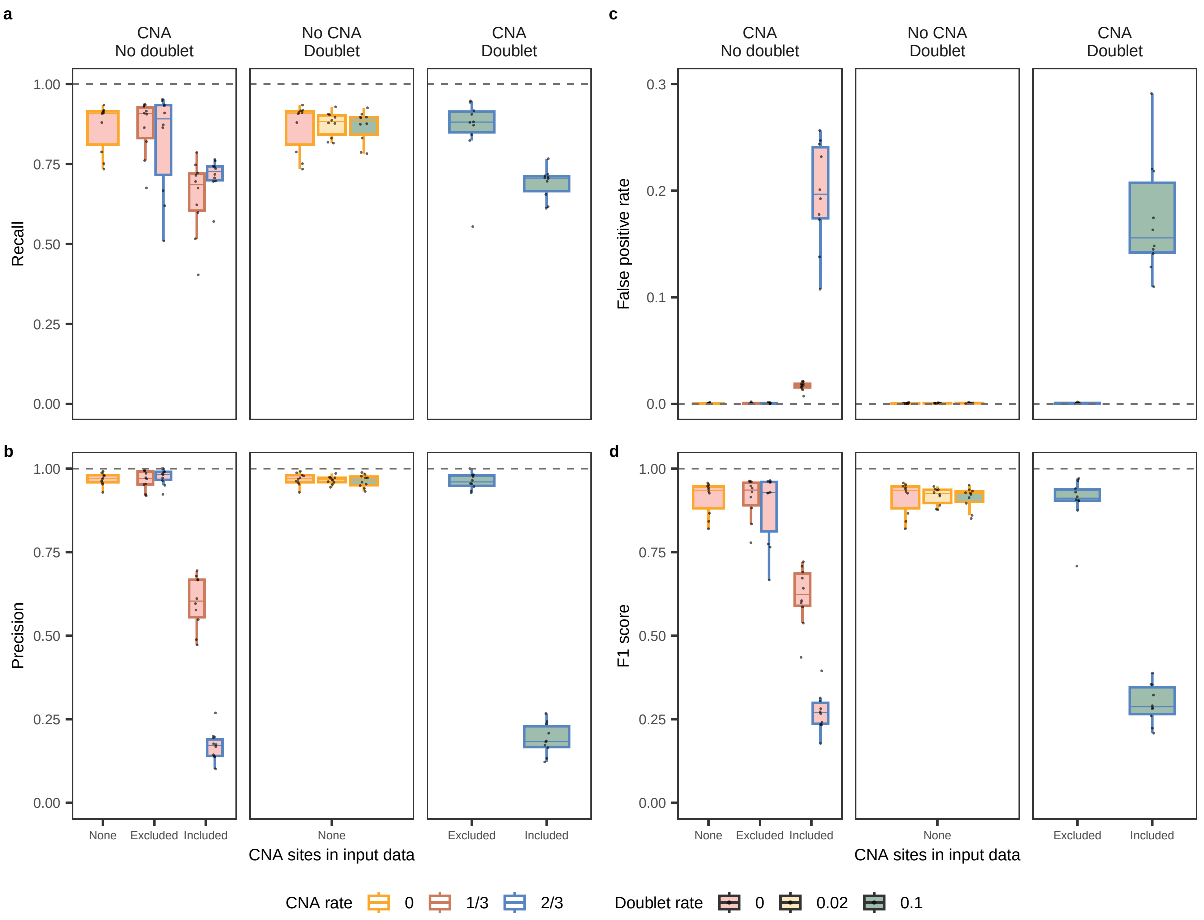

Fig. S13: Reference-remaining genotype calling results of the DelSIEVE model considering CNAs and/or doublets. Varying are the status of CNA sites in input data (the horizontal axis), the CNA rate (the boxes with colored frames), the doublet rate (the boxes filled with colors), as well as the simulation scenarios of CNAs and doublets (the vertical strip). Each simulation is repeated $n = 10$ times, with each repetition denoted by a black dot. The gray dashed lines represent the optimal values of each metric. Box plots comprise medians, boxes covering the interquartile range (IQR), and whiskers extending to 1.5 times the IQR below and above the box. **a-d**, Box plots of the reference-remaining genotype calling results measured by recall (**a**), precision (**b**), false positive rate (**c**) and F1 score (**d**).

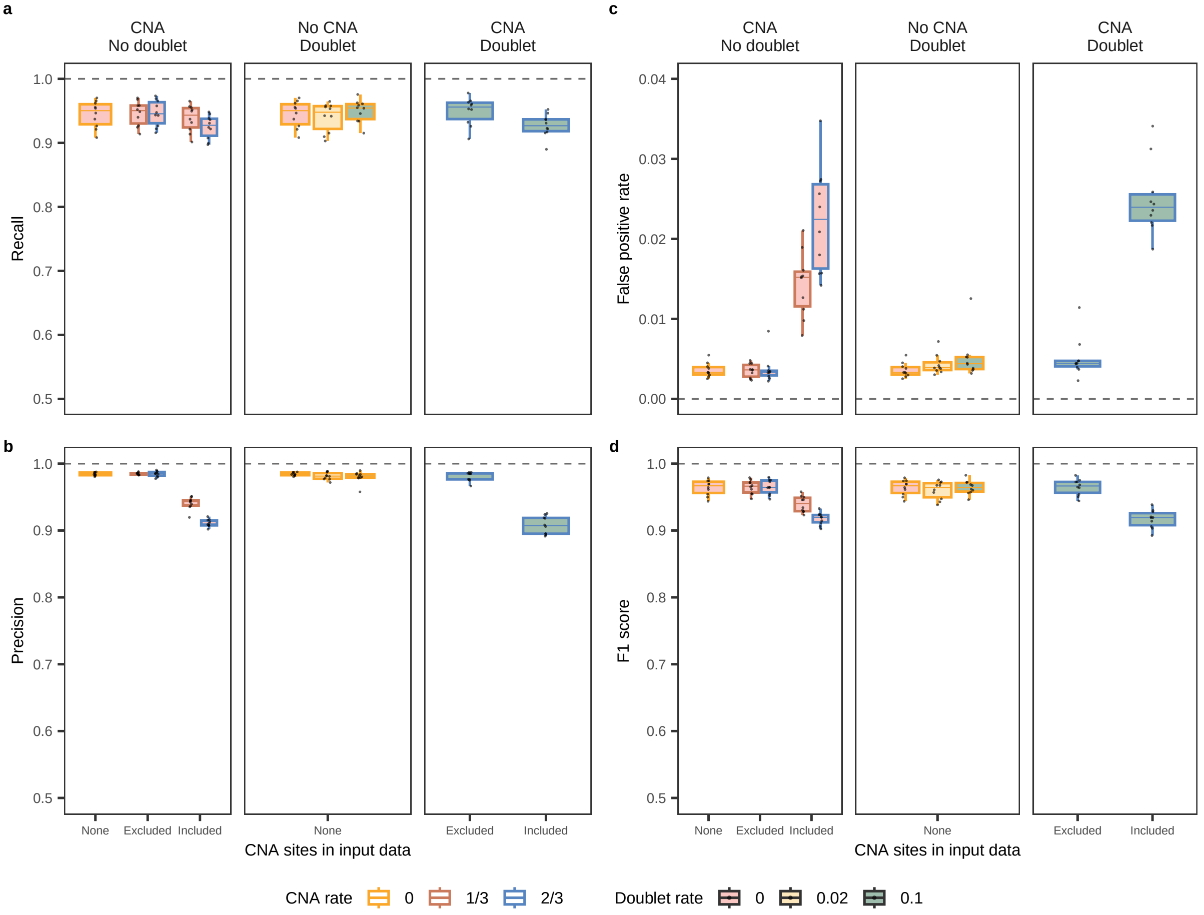

Fig. S14: Single mutant genotype calling results of the DelSIEVE model considering CNAs and/or doublets. Varying are the status of CNA sites in input data (the horizontal axis), the CNA rate (the boxes with colored frames), the doublet rate (the boxes filled with colors), as well as the simulation scenarios of CNAs and doublets (the vertical strip). Each simulation is repeated $n = 10$ times, with each repetition denoted by a black dot. The gray dashed lines represent the optimal values of each metric. Box plots comprise medians, boxes covering the interquartile range (IQR), and whiskers extending to 1.5 times the IQR below and above the box. **a-d**, Box plots of the single mutant genotype calling results measured by recall (**a**), precision (**b**), false positive rate (**c**) and F1 score (**d**).

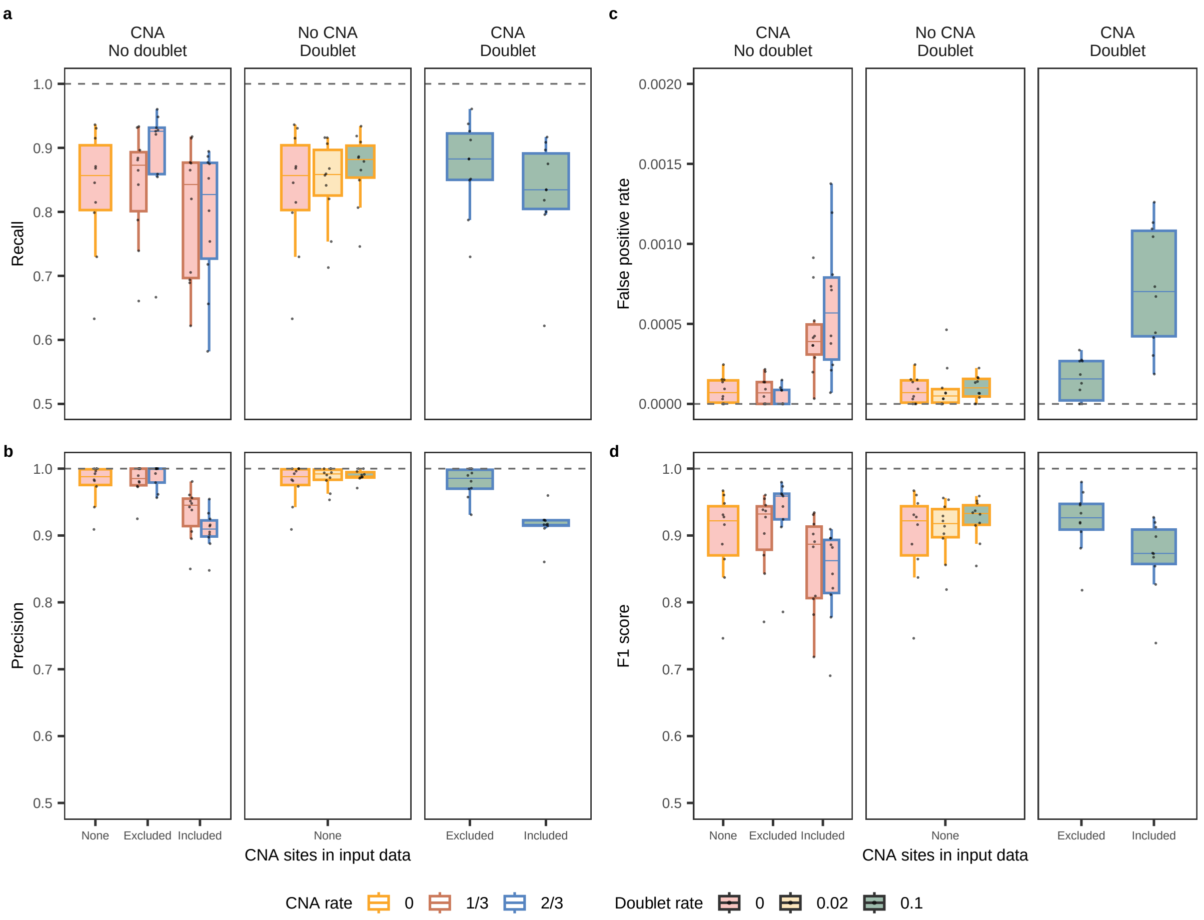

Fig. S15: Double mutant genotype calling results of the DelSIEVE model considering CNAs and/or doublets. Varying are the status of CNA sites in input data (the horizontal axis), the CNA rate (the boxes with colored frames), the doublet rate (the boxes filled with colors), as well as the simulation scenarios of CNAs and doublets (the vertical strip). Each simulation is repeated $n = 10$ times, with each repetition denoted by a black dot. The gray dashed lines represent the optimal values of each metric. Box plots comprise medians, boxes covering the interquartile range (IQR), and whiskers extending to 1.5 times the IQR below and above the box. **a-d**, Box plots of the double mutant genotype calling results measured by recall (**a**), precision (**b**), false positive rate (**c**) and F1 score (**d**).

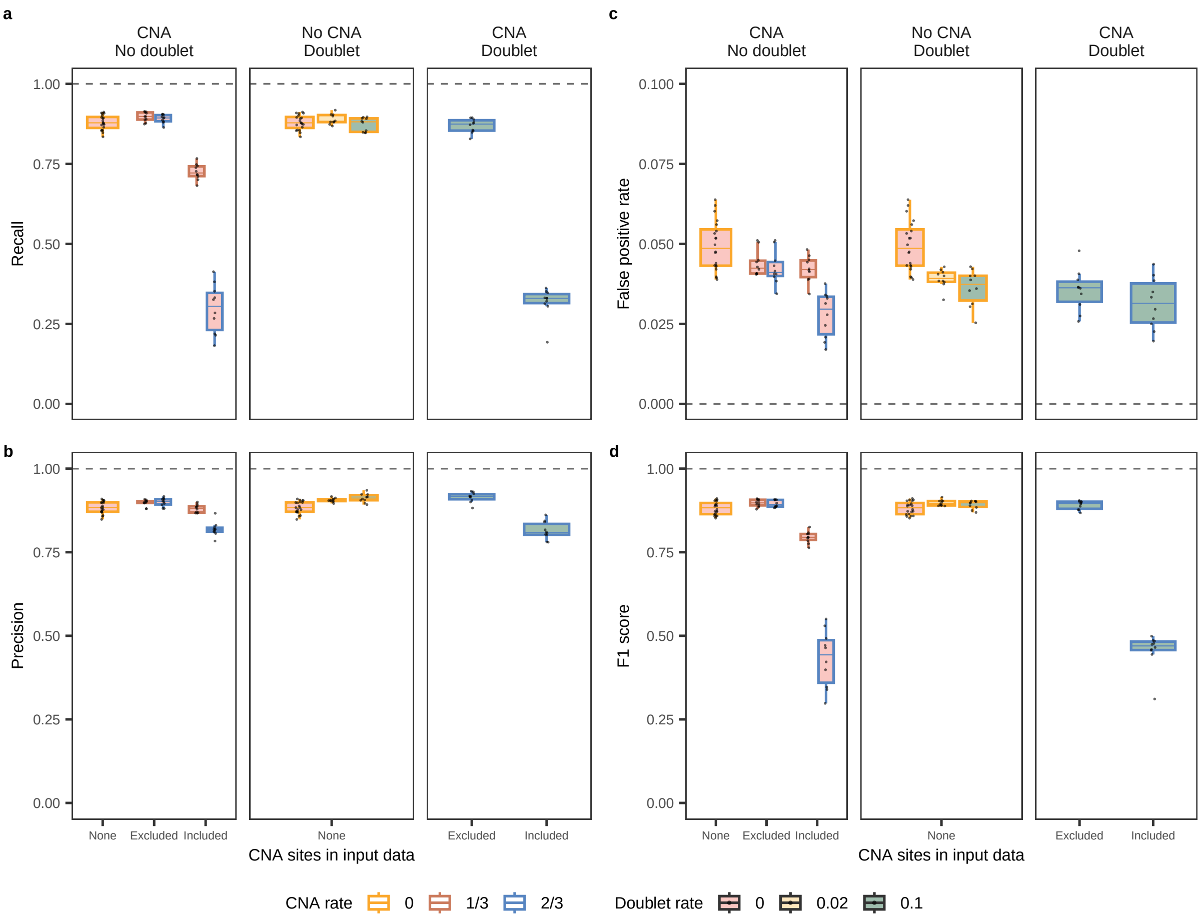

Fig. S16: Single ADO calling results of the DelSIEVE model considering CNAs and/or doublets. Varying are the status of CNA sites in input data (the horizontal axis), the CNA rate (the boxes with colored frames), the doublet rate (the boxes filled with colors), as well as the simulation scenarios of CNAs and doublets (the vertical strip). Each simulation is repeated $n = 10$ times, with each repetition denoted by a black dot. The gray dashed lines represent the optimal values of each metric. Box plots comprise medians, boxes covering the interquartile range (IQR), and whiskers extending to 1.5 times the IQR below and above the box. **a-d**, Box plots of the single ADO calling results measured by recall (**a**), precision (**b**), false positive rate (**c**) and F1 score (**d**).

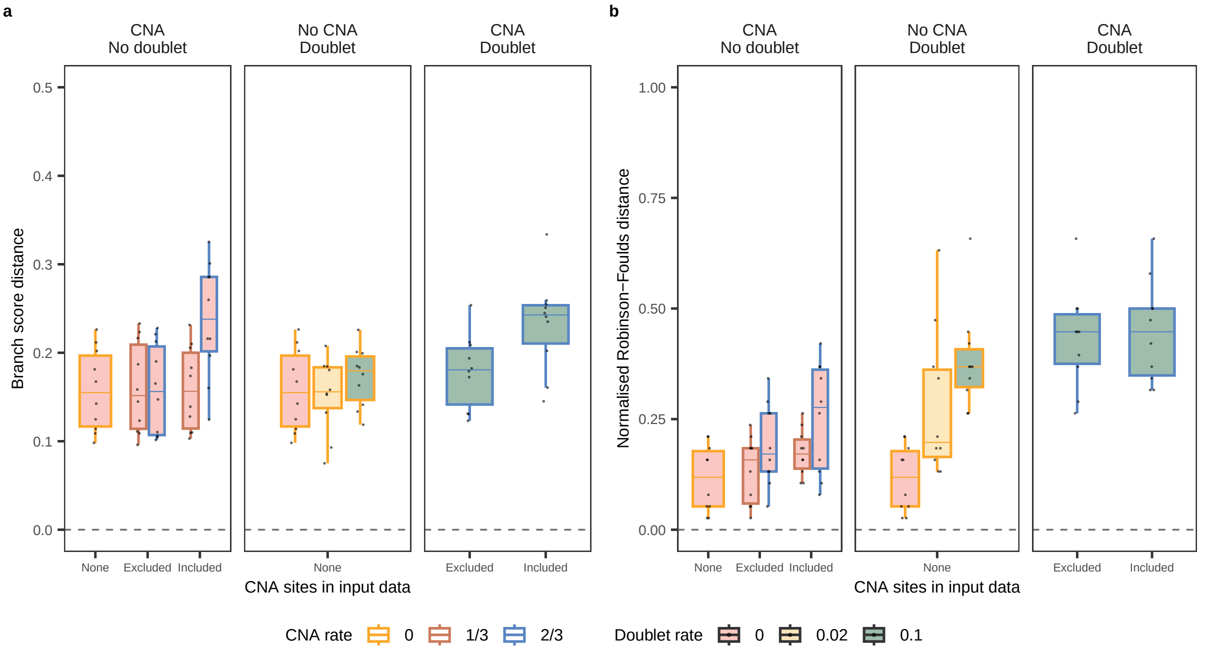

Fig. S17: Tree inference results of the DelSIEVE model considering CNAs and/or doublets. Varying are the status of CNA sites in input data (the horizontal axis), the CNA rate (the boxes with colored frames), the doublet rate (the boxes filled with colors), as well as the simulation scenarios of CNAs and doublets (the vertical strip). Each simulation is repeated $n = 10$ times, with each repetition denoted by a black dot. The gray dashed lines represent the optimal values of each metric. Box plots comprise medians, boxes covering the interquartile range (IQR), and whiskers extending to 1.5 times the IQR below and above the box. **a-b**, Box plots of the tree inference results measured by BS distance where the branch lengths are taken into account (**a**) and normalized RF distance where only tree topology is considered (**b**).

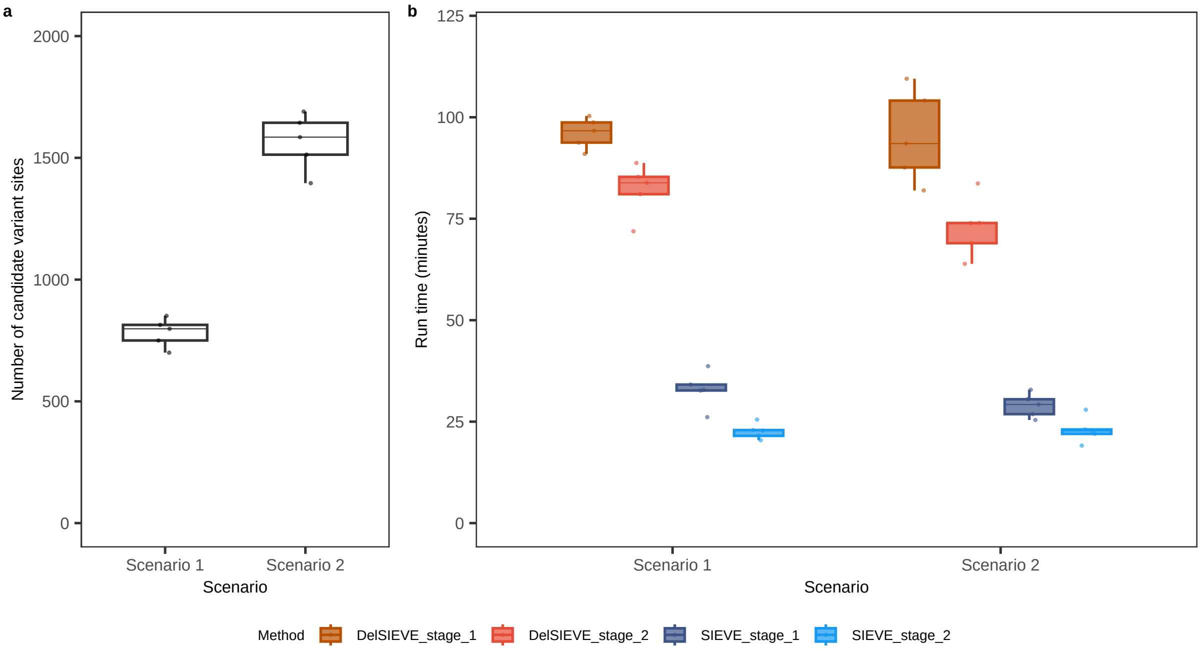

Fig. S18: Runtime evaluation of DelSIEVE against SIEVE. Varying are the simulation scenario of different numbers of candidate variant sites (the horizontal axis) and the run time for 10^5 iterations (colored boxes). Each simulation is repeated $n = 5$ times, with each repetition denoted by a dot. Box plots comprise medians, boxes covering the interquartile range (IQR), and whiskers extending to 1.5 times the IQR below and above the box. **a-b**, Box plots of the number of candidate variant sites of datasets in each simulation scenario (**a**) and the run time for 10^5 iterations for both stages of DelSIEVE and SIEVE (**b**).

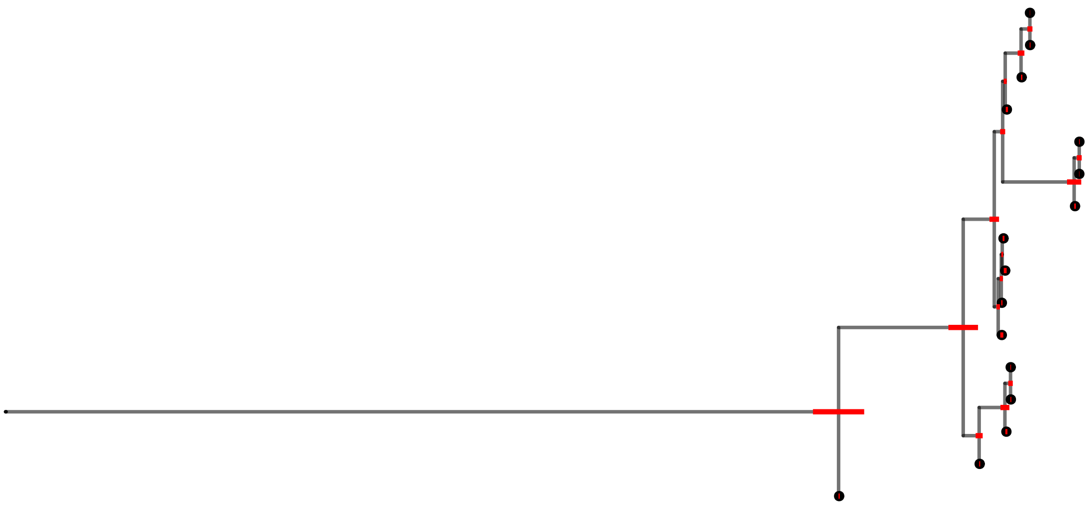

Fig. S19: Illustration of branch lengths of the phylogenetic tree inferred from TNBC16 [35] by DelSIEVE. Shown is exactly the same tree as in Figure 4, except that cell names, subclone posterior probabilities and gene annotations are removed and no branches are folded. Red bars annotated to internal nodes except the root are the 95% HPD intervals of the corresponding branch lengths.

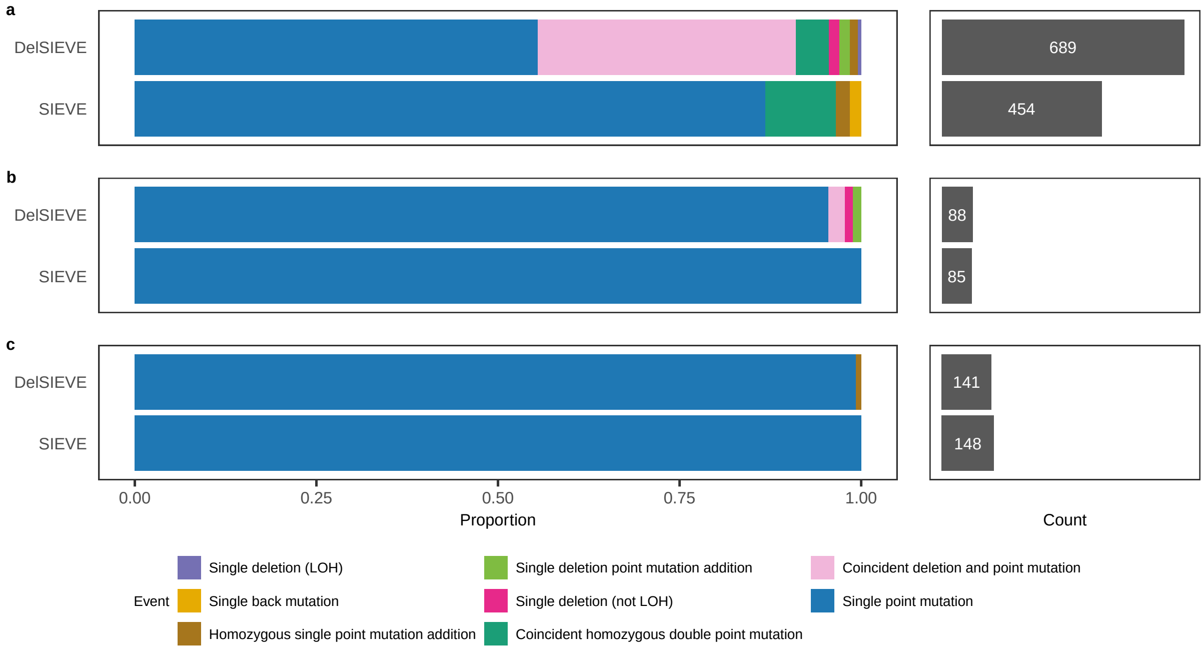

Fig. S20: Distribution of mutation event types in non-synonymous genes analyzed by DelSIEVE and SIEVE for all real datasets. For each real dataset, the column to the left is the proportions of mutation event types, while the column to the right is the total counts of all mutation event type. Mutation event types are of the same colors as in the corresponding trees. **a-c**, Distribution of mutation event types in non-synonymous genes analyzed by DelSIEVE and SIEVE for TNBC16 [35] (**a**), CRC28 [32] (**b**), and CRC48 [40] (**c**).

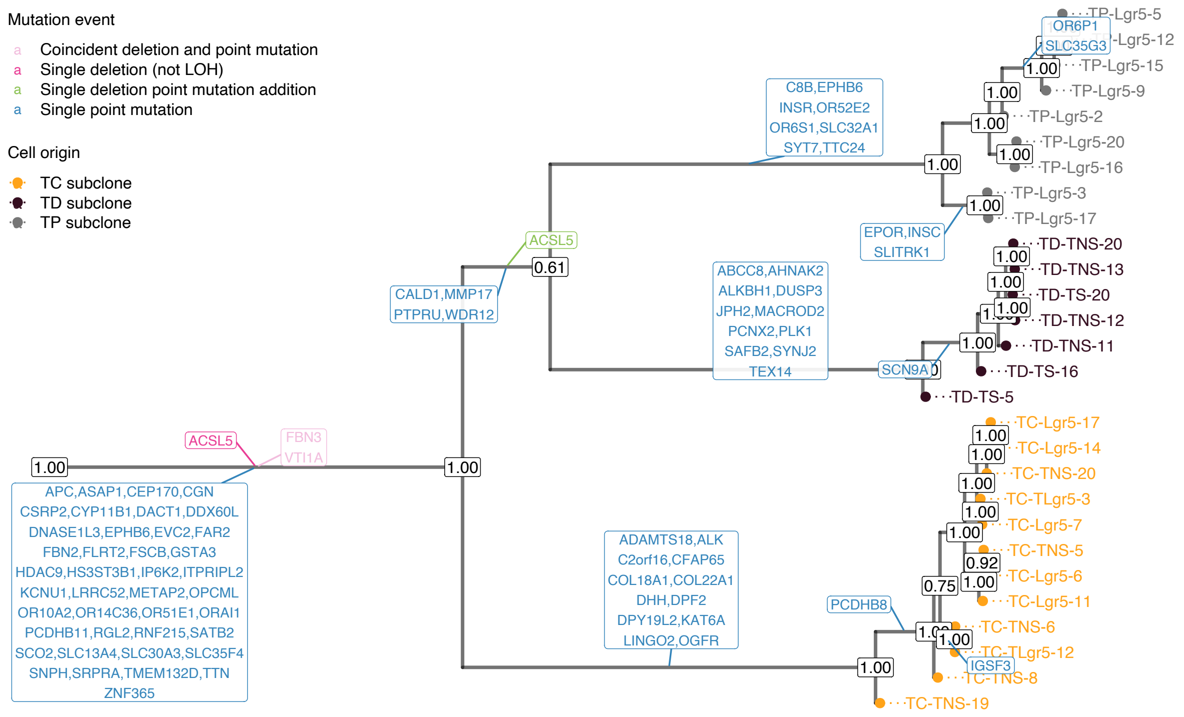

Fig. S21: Results of phylogenetic inference for the CRC28 [32] dataset. Shown is DelSIEVE's maximum clade credibility tree. Tumor cell names are annotated to the leaves of the tree. The exceptionally long trunk has been folded (marked by slashes). Cells are colored according to the corresponding biopsies. The numbers at each node represent the posterior probabilities (threshold $p > 0.5$). At each branch, depicted in different colors are non-synonymous genes that are either CRC-related single mutations (in blue) or other mutation events (in other colors).

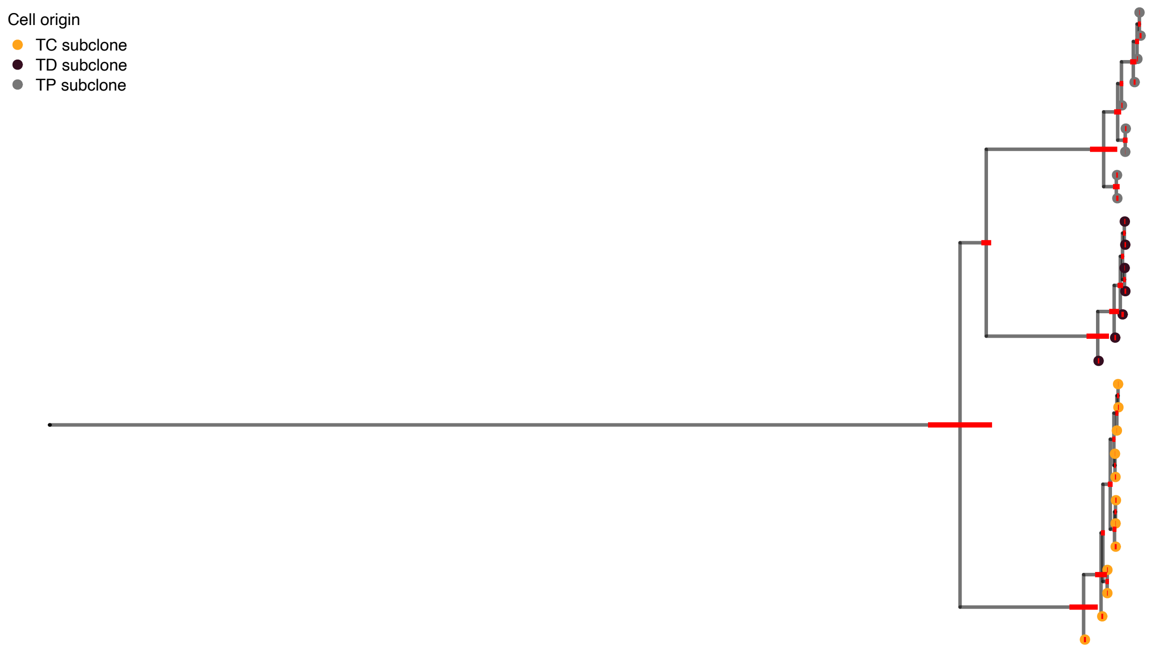

Fig. S22: Illustration of branch lengths of the phylogenetic tree inferred from CRC28 [18] by DelSIEVE. Shown is exactly the same tree as in Figure S21, except that cell names, subclone posterior probabilities and gene annotations are removed and no branches are folded. Red bars annotated to internal nodes except the root are the 95% HPD intervals of the corresponding branch lengths.

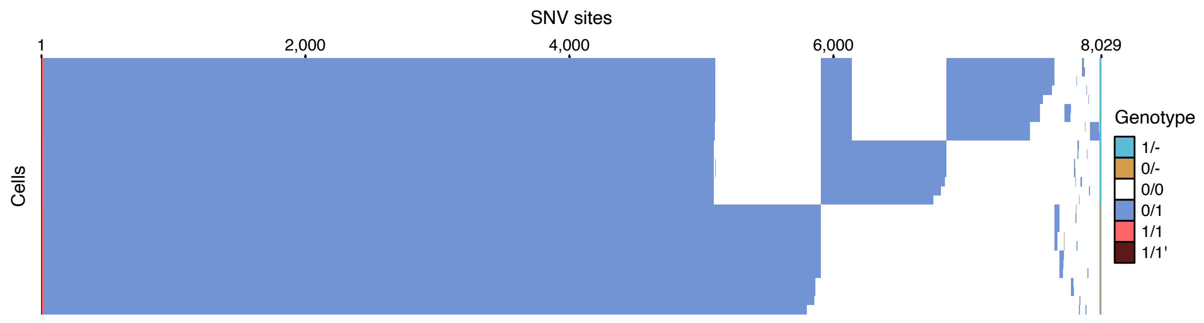

Fig. S23: Results of variant calling for the CRC28 dataset [18]. Cells in the row are in the same order as that of leaves in the phylogenetic tree in Figure S21.

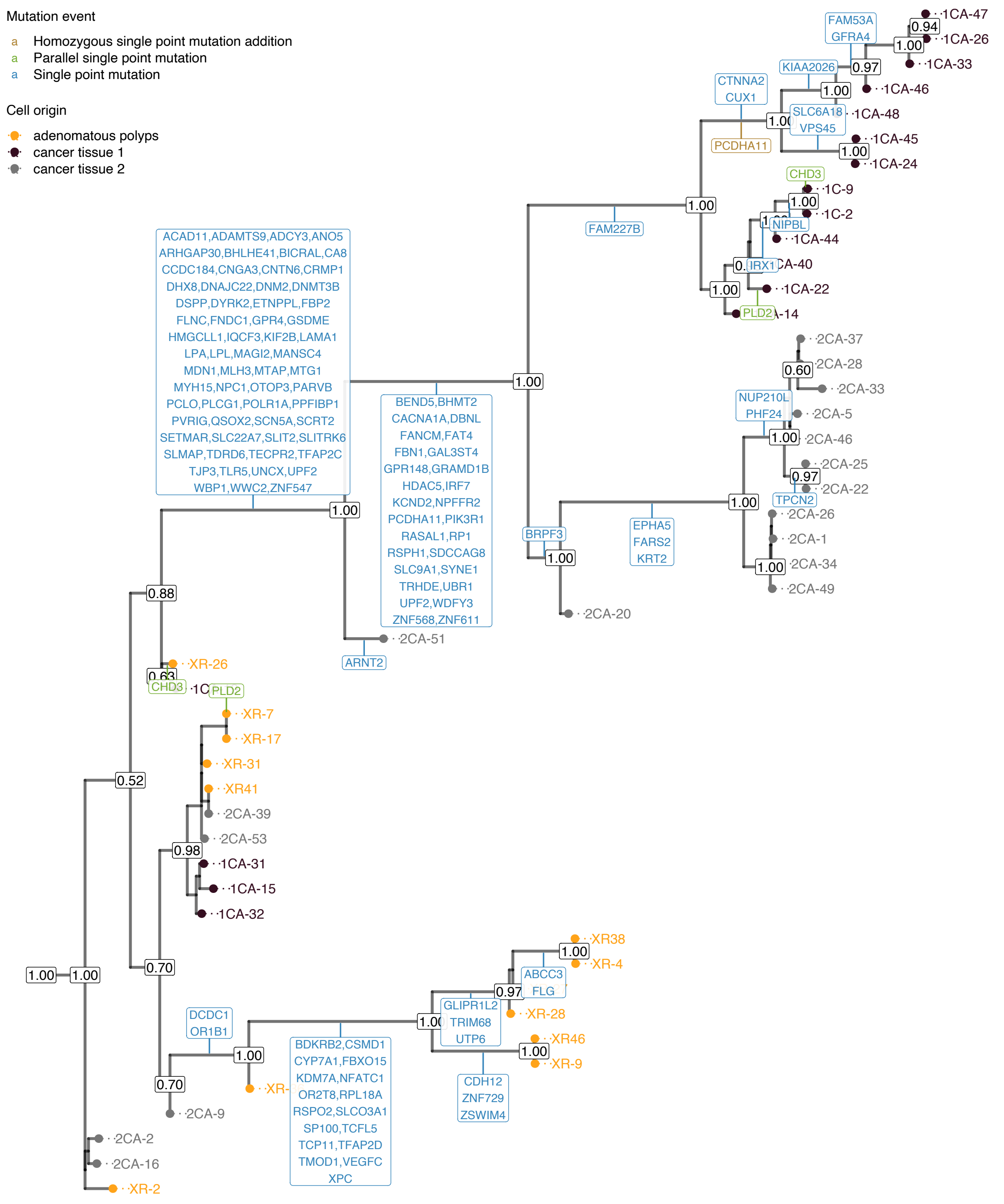

Fig. S24: Results of phylogenetic inference for the CRC48 dataset [40]. Shown is DelSIEVE's maximum clade credibility tree. Tumor cell names are annotated to the leaves of the tree. Three exceptionally long branches are folded with the number of slashes proportional to the branch lengths. Cells are colored according to the corresponding biopsies. The numbers at each node represent the posterior probabilities (threshold $p > 0.5$). At each branch, depicted in different colors are non-synonymous genes that are either CRC-related single mutations (in blue) or other mutation events (in other colors).

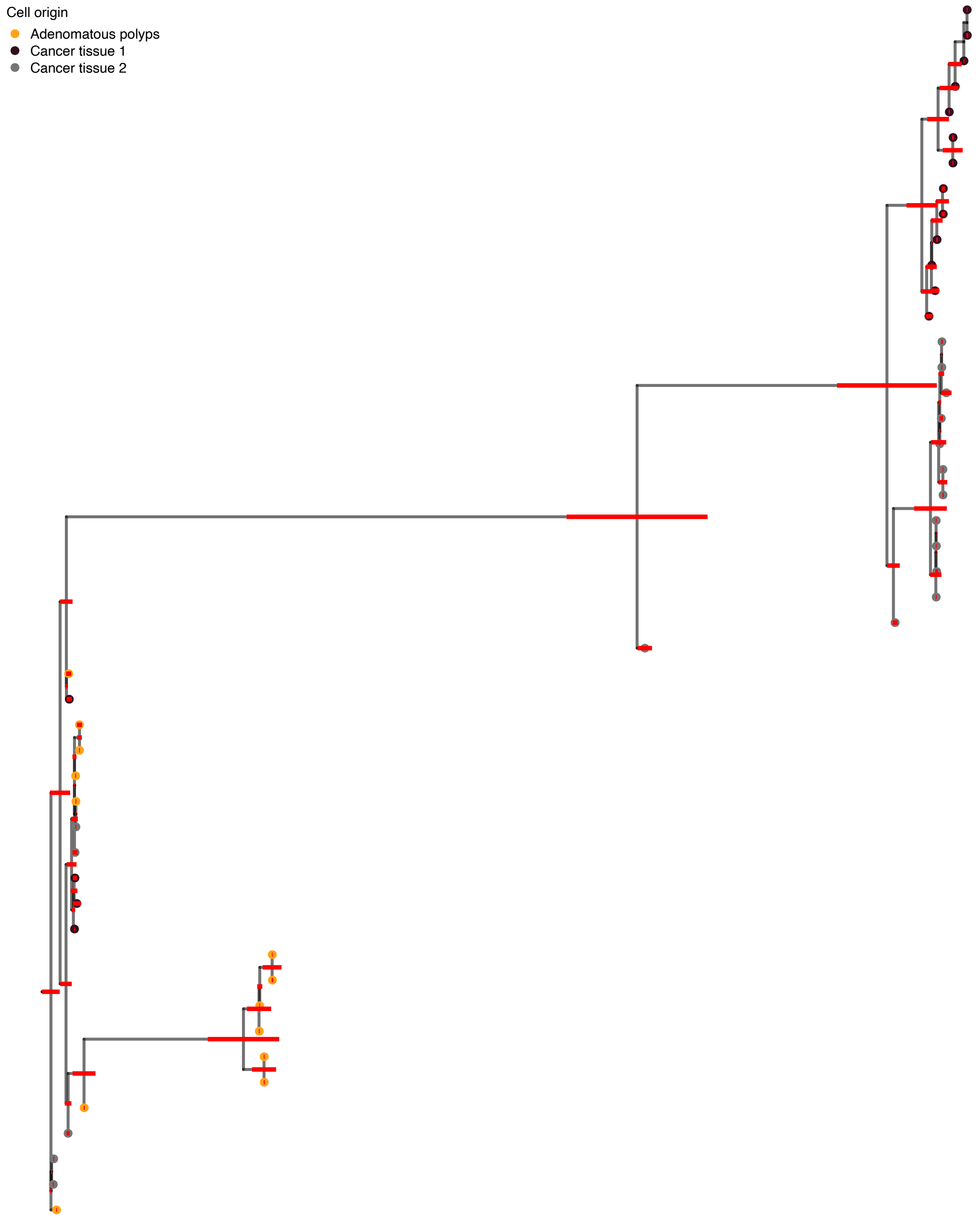

Fig. S25: Illustration of branch lengths of the phylogenetic tree inferred from CRC48 [40] by DelSIEVE. Shown is exactly the same tree as in Figure S24, except that cell names, subclone posterior probabilities and gene annotations are removed and no branches are folded. Red bars annotated to internal nodes except the root are the 95% HPD intervals of the corresponding branch lengths.

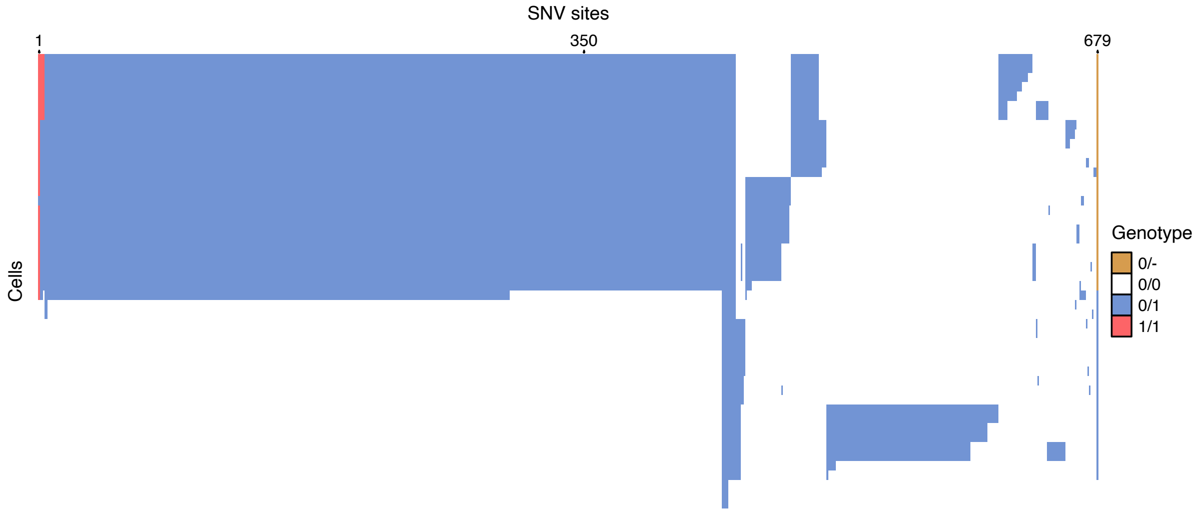

Fig. S26: Results of variant calling for the CRC48 dataset [40]. Cells in the row are in the same order as that of leaves in the phylogenetic tree in Figure S24.

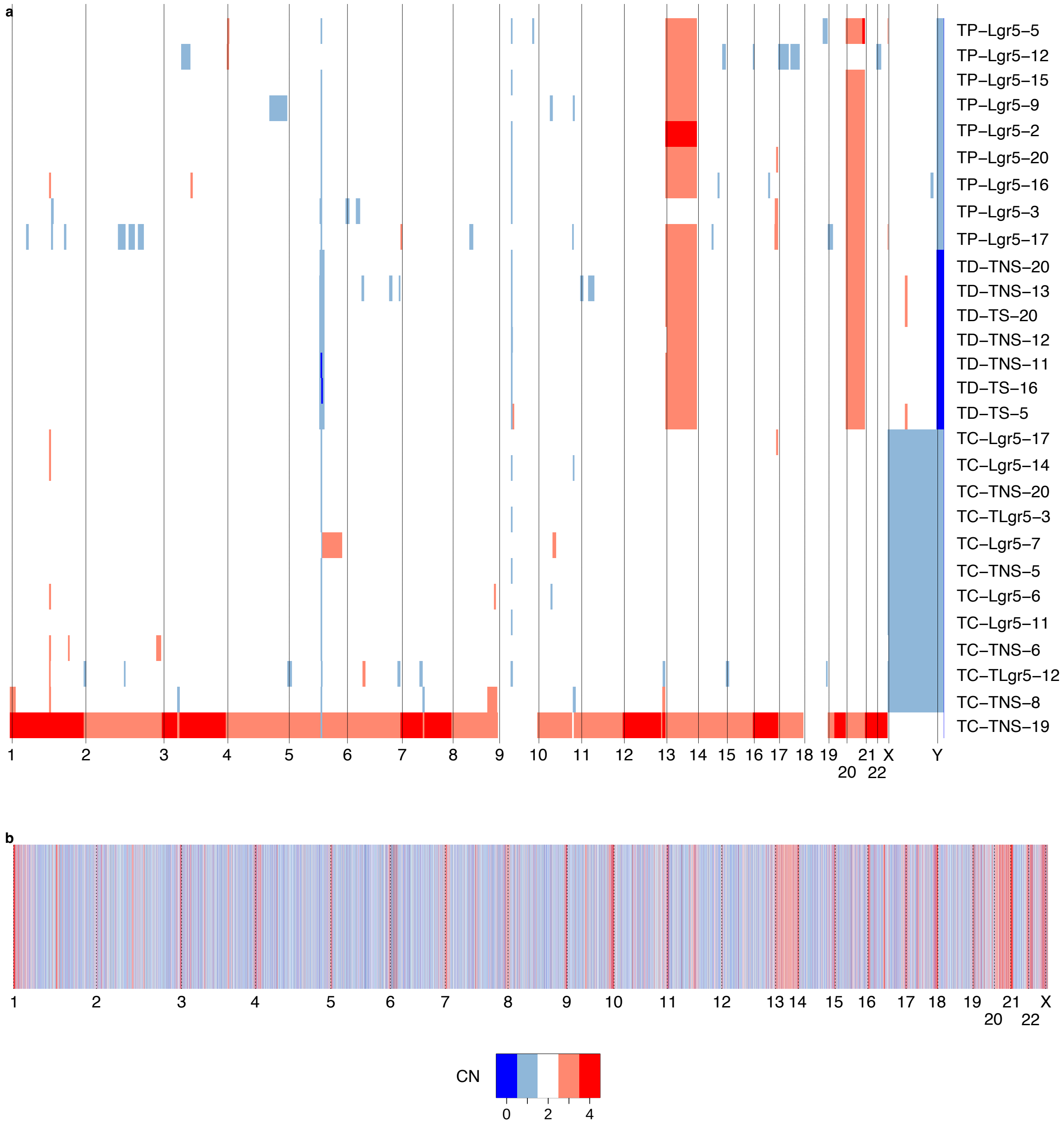

Fig. S27: Heatmaps of copy numbers (CNs) across whole genome of cells in CRC28 [18] reported by Ginkgo and Sequenza. The horizontal axis represents the indices of chromosomesm, and the vertical axis represents either single cells (in **a**, by Ginkgo) or bulk-seq sample (in **b**, by Sequenza).

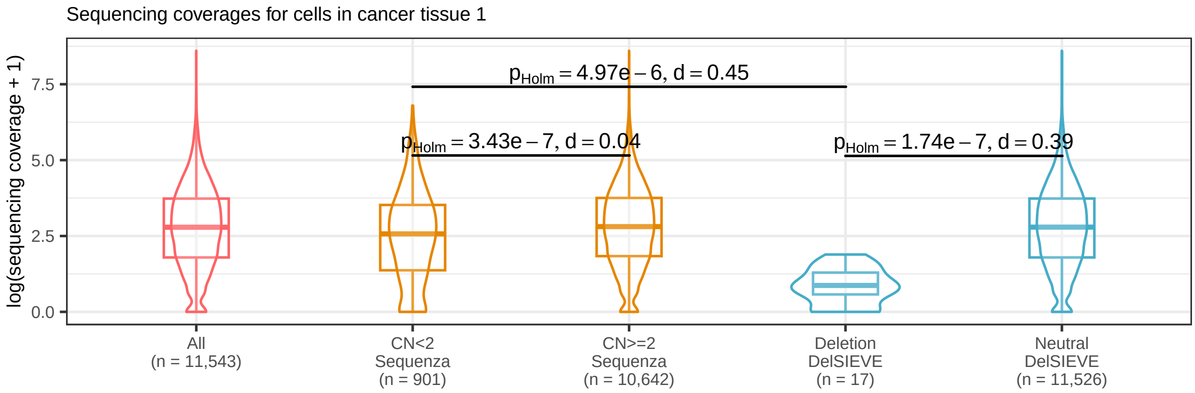

Fig. S28: Results of subclone-wise sequencing coverage comparison for cells in cancer tissue 1 of CRC48 [40] between DelSIEVE and Sequenza. Compared were the sites shared between the input data of both methods. The resolution of variant calling was subclone-wise in order to conduct a fair comparison. For Sequenza, sites were divided into two groups with copy number (CN) < 2 and ≥ 2, respectively. For DelSIEVE, sites were divided into two groups, one with deletions, the other copy neutral. Sequencing coverage transformed with $\log p1$ across those cells in the subclone at all sites were plotted for reference. In each group, the violin and the box plots showed the distribution of the sequencing coverage. The total number of dots in each group, which was the product of the number of cells (17) and the number of sites in each group, was marked with n on the horizontal axis. Box plots comprise medians, boxes covering the interquartile range (IQR), and whiskers extending to 1.5 times the IQR below and above the box. Within- and between-group comparisons were conducted between CN < 2 and ≥ 2 of Sequenza, between deletions and copy neutral of DelSIEVE, and between CN < 2 of Sequenza and deletions of DelSIEVE. Each comparison was conducted on the sequencing coverage on the original scale, showing the result of Mann-Whitney U test, with the p-value corrected by Holm–Bonferroni method and the absolute value of the effect size (Cohen's d).

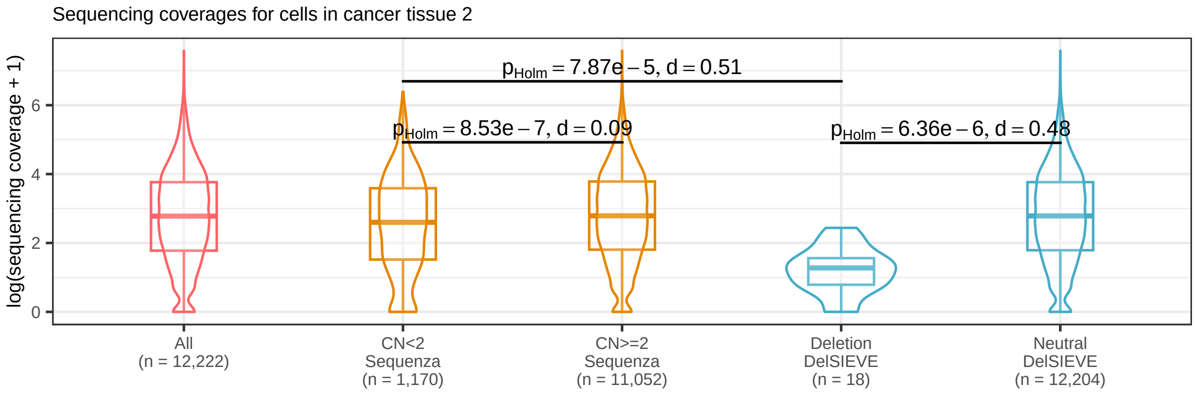

Fig. S29: Results of subclone-wise sequencing coverage comparison for cells in cancer tissue 2 of CRC48 [40] between DelSIEVE and Sequenza. Compared were the sites shared between the input data of both methods. The resolution of variant calling was subclone-wise in order to conduct a fair comparison. For Sequenza, sites were divided into two groups with copy number (CN) < 2 and ≥ 2, respectively. For DelSIEVE, sites were also divided into two groups, one with deletions, the other copy neutral. Sequencing coverage transformed with $\log p1$ across those cells in the subclone at all sites were plotted for reference. In each group, the violin and the box plots showed matched the color of the method and the distribution of the sequencing coverage. The total number of dots in each group, which was the product of the number of cells (18) and the number of sites in each group, was marked with n on the horizontal axis. Box plots comprise medians, boxes covering the interquartile range (IQR), and whiskers extending to 1.5 times the IQR below and above the box. Within- and between-group comparisons were conducted between CN < 2 and ≥ 2 of Sequenza, between deletions and copy neutral of DelSIEVE, and between CN < 2 of Sequenza and deletions of DelSIEVE. Each comparison was conducted on the sequencing coverage on the original scale, showing the result of Mann-Whitney U test, with the p-value corrected by Holm–Bonferroni method and the absolute value of the effect size (Cohen's d).

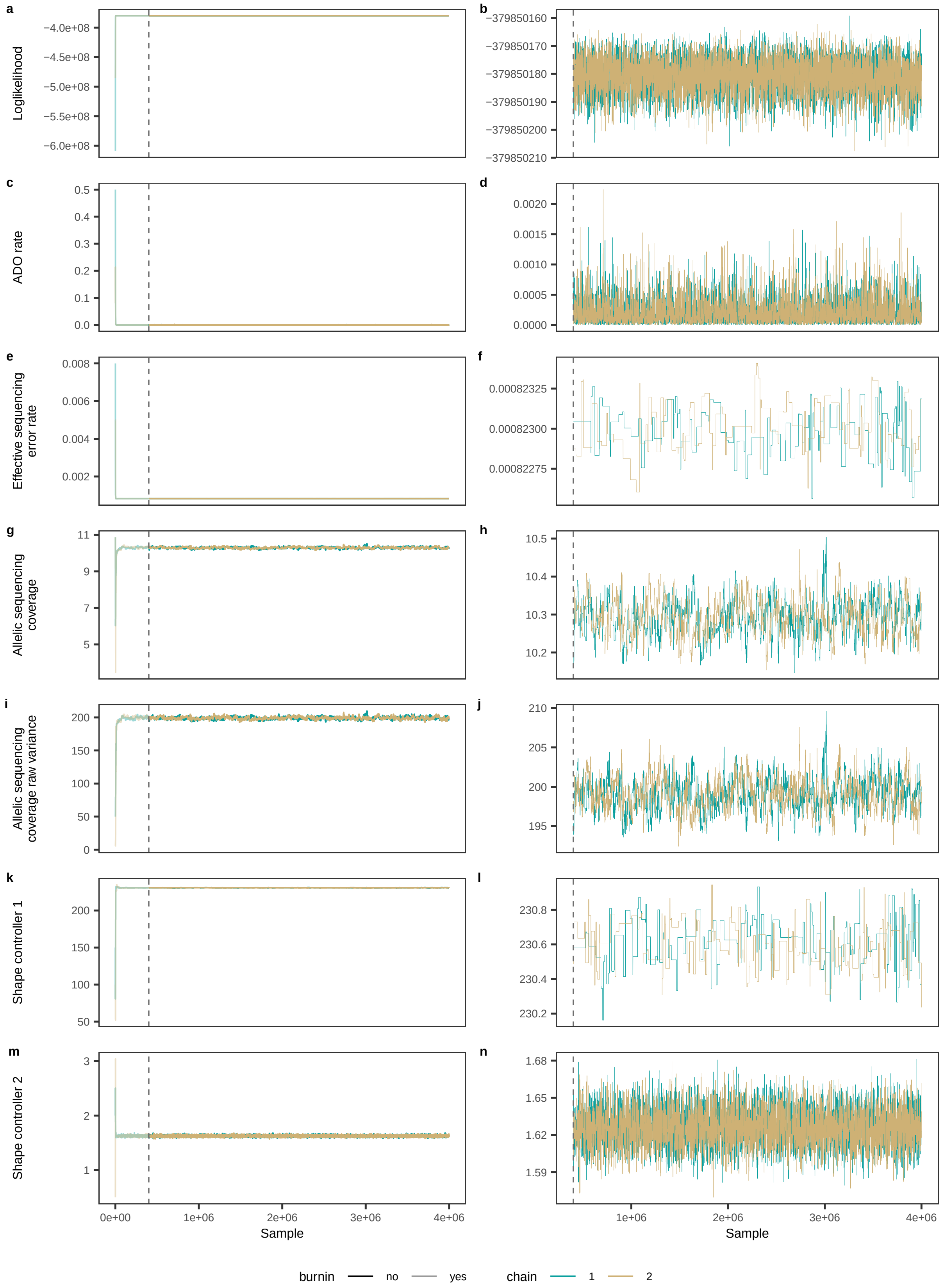

a
b
c
d
e
f
g
h
i
j
k
l
m
n
Loglikelihood
ADO rate
Effective sequencing error rate
Allelic sequencing coverage
Allelic sequencing coverage raw variance
Shape controller 1
Shape controller 2
Sample
Sample
burnin
no
yes
chain
1
2

Fig. S30 ***(previous page)*****: Trace plots of two individual MCMC chains underlying DelSIEVE for TNBC16 [35].** The vertical dashed line means that samples taken before it (10% of all samples) are discarded as they are from the burn-in phase. The columns to the left plot all samples, while the columns to the right plot only samples that are not from the burn-in phase. **a-n**, Trace plots for loglikelihood (**a-b**), ADO rate (**c-d**), effective sequencing error rate (**e-f**), allelic sequencing coverage (**g-h**), allelic sequencing coverage raw variance (**i-j**), shape controller 1 (**k-l**), and shape controller 2 (**m-n**).

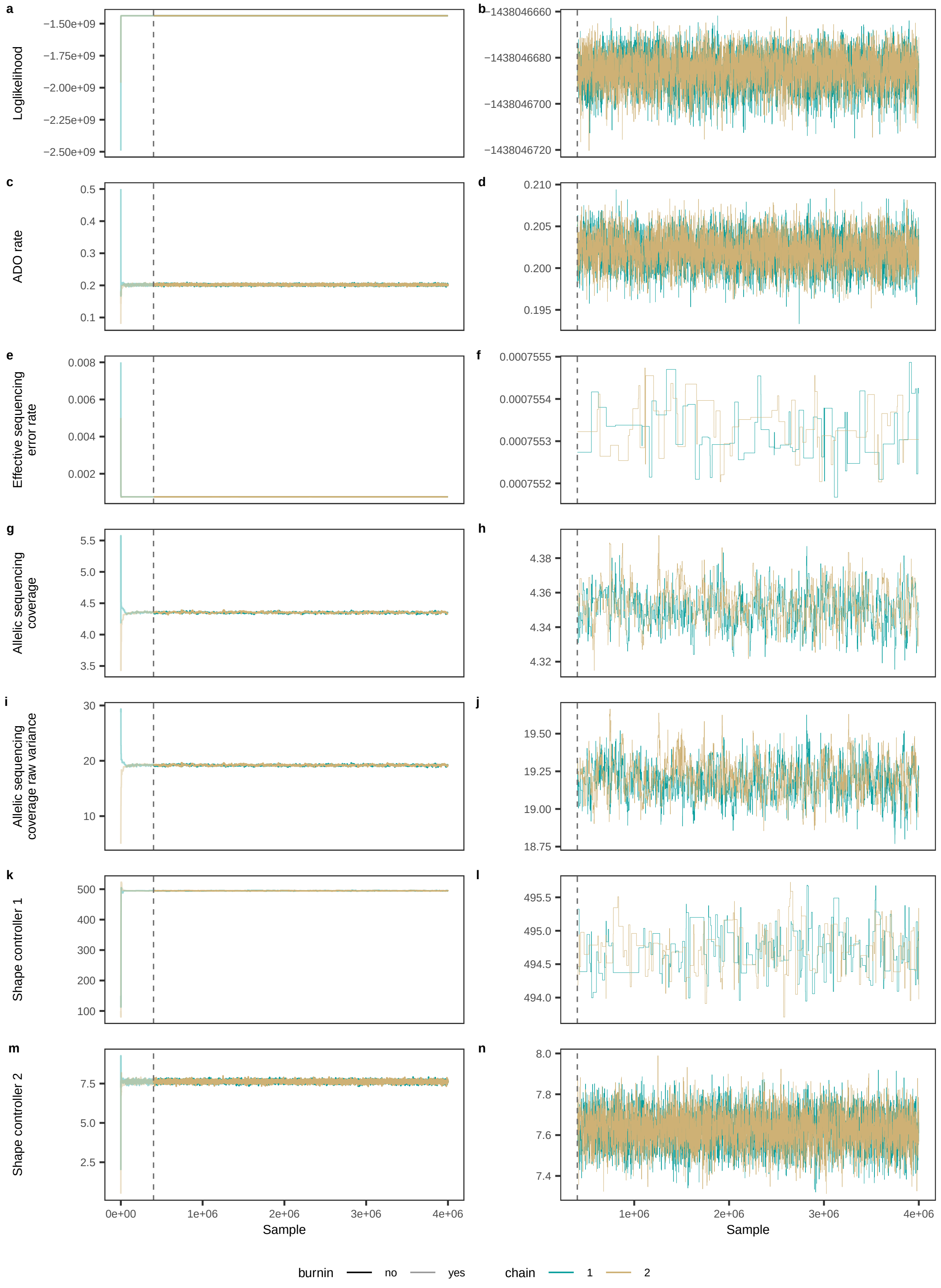
a
b
c
d
e
f
g
h
i
j
k
l
m
n
Loglikelihood
ADO rate
Effective sequencing error rate
Allelic sequencing coverage
Allelic sequencing coverage raw variance
Shape controller 1
Shape controller 2
Sample
Sample
burnin
no
yes
chain
1
2

Fig. S31 ***(previous page)*****: Trace plots of two individual MCMC chains underlying DelSIEVE for CRC28 [32].** The vertical dashed line means that samples taken before it (10% of all samples) are discarded as they are from the burn-in phase. The columns to the left plot all samples, while the columns to the right plot only samples that are not from the burn-in phase. **a-n**, Trace plots for loglikelihood (**a-b**), ADO rate (**c-d**), effective sequencing error rate (**e-f**), allelic sequencing coverage (**g-h**), allelic sequencing coverage raw variance (**i-j**), shape controller 1 (**k-l**), and shape controller 2 (**m-n**).

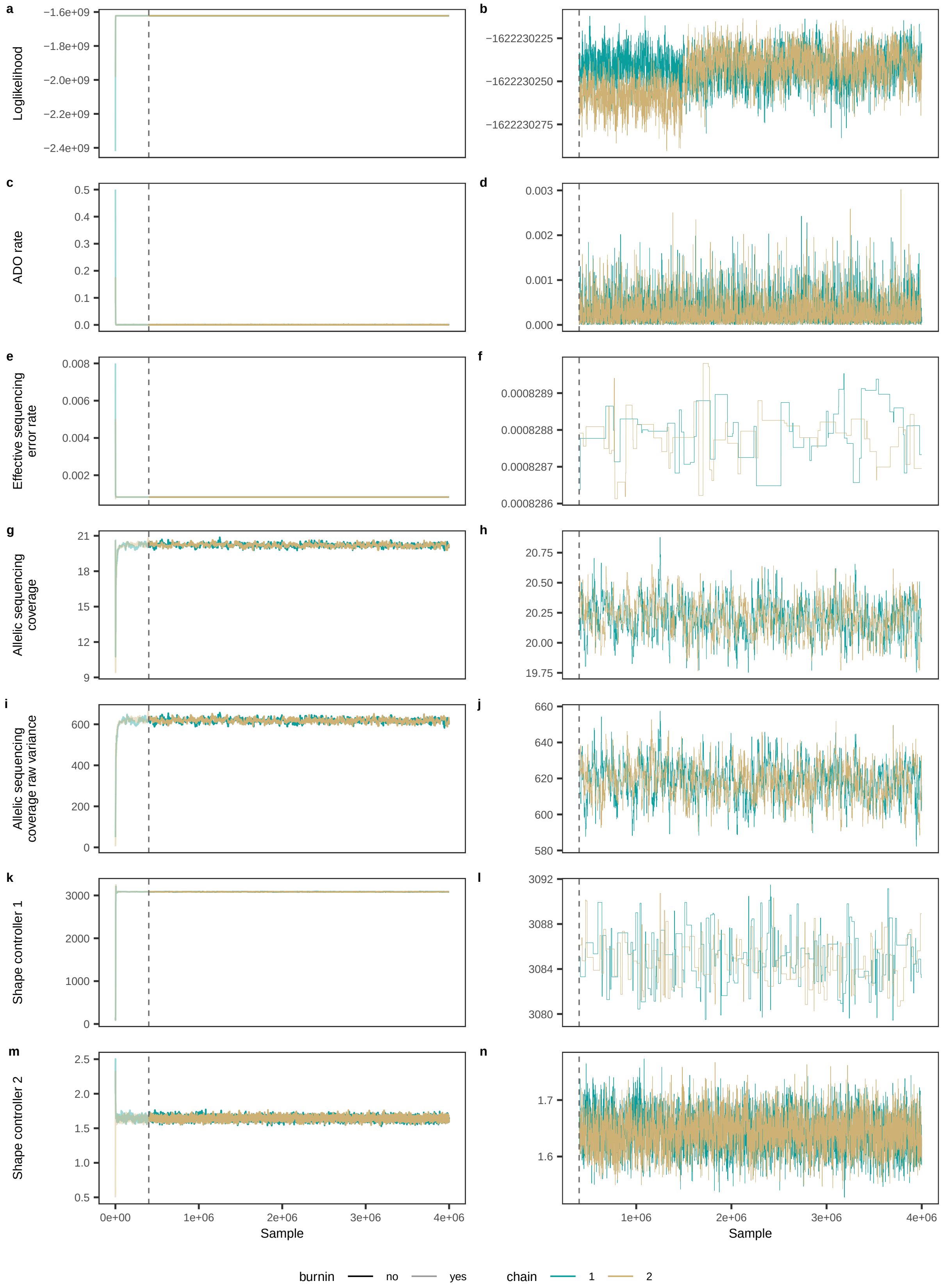
a
b
c
d
e
f
g
h
i
j
k
l
m
n
Loglikelihood
ADO rate
Effective sequencing error rate
Allelic sequencing coverage
Allelic sequencing coverage raw variance
Shape controller 1
Shape controller 2
Sample
burnin no yes
chain 1 2

Fig. S32 ***(previous page)*****: Trace plots of two individual MCMC chains underlying DelSIEVE for CRC48 [40].** The vertical dashed line means that samples taken before it (10% of all samples) are discarded as they are from the burn-in phase. The columns to the left plot all samples, while the columns to the right plot only samples that are not from the burn-in phase. **a-n**, Trace plots for loglikelihood (**a-b**), ADO rate (**c-d**), effective sequencing error rate (**e-f**), allelic sequencing coverage (**g-h**), allelic sequencing coverage raw variance (**i-j**), shape controller 1 (**k-l**), and shape controller 2 (**m-n**).

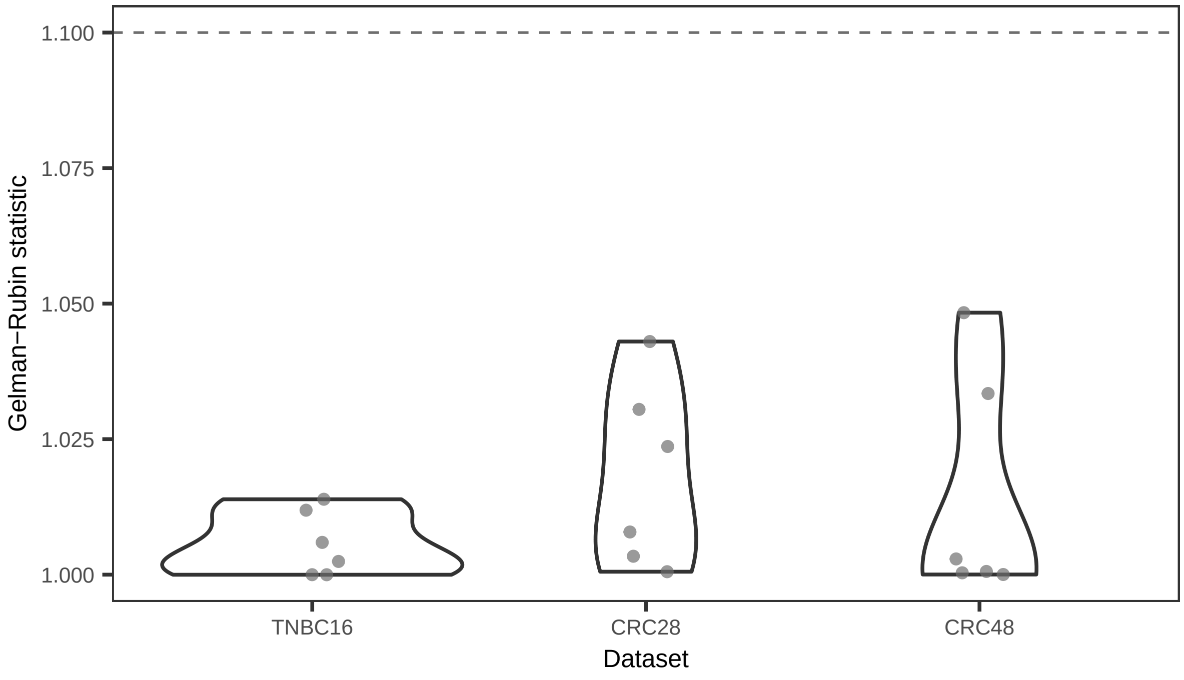

Fig. S33: Violin plot of the Gelman-Rubin statistic to diagnose MCMC chains for real datasets. Each point in the plot correspond to a hidden random variable plotted in Figures S30 to S32, except for the loglikelihood. The horizontal dashed line is the threshold below which the MCMC chain is considered converged.
