# Supplementary material for: DelSIEVE: cell phylogeny modeling of single nucleotide variants and deletions from single-cell DNA sequencing data
Source: Genome Biol. 2025 Aug 25;26:255. doi: 10.1186/s13059-025-03738-9 (PMC12376439; doi:10.1186/s13059-025-03738-9)
Supplement: Supplementary file 3 — Additional file 3: Supplementary tables S1-S4. [file 13059_2025_3738_MOESM3_ESM.pdf]

DelSIEVE: cell phylogeny modeling of single nucleotide variants and deletions from single-cell DNA sequencing data

Supplementary tables

Senbai Kang[1], Nico Borgsmüller[2,3], Monica Valecha[4,5], Magda Markowska[1,6], Jack Kuipers[2,3], Niko Beerenwinkel[2,3], David Posada[4,5,7], and Ewa Szczurek[8,1*]

[1] *Faculty of Mathematics, Informatics and Mechanics, University of Warsaw, Warsaw, Poland*

[2] *Department of Biosystems Science and Engineering, ETH Zurich, 4058 Basel, Switzerland*

[3] *SIB Swiss Institute of Bioinformatics, 4058 Basel, Switzerland*

[4] *CINBIO, Universidade de Vigo, 36310 Vigo, Spain*

[5] *Galicia Sur Health Research Institute (IIS Galicia Sur), SERGAS-UVIGO*

[6] *Medical University of Warsaw, Postgraduate School of Molecular Medicine, Warsaw, Poland*

[7] *Department of Biochemistry, Genetics, and Immunology, Universidade de Vigo, 36310 Vigo, Spain*

[8] *Institute of AI for Health, Helmholtz Zentrum München, German Research Center for Environmental Health, Neuherberg, Germany*

[*] *Correspondence: ewa.szczurek@helmholtz-munich.de*

Table S1: Definition of the distribution of the number of sequenced alleles (α_{ij}) conditional on the true genotype (g_{ij}) and on the allelic ADO rate (θ_A) under the ADO mode for DelSIEVE.

α_{ij}	g_{ij}	ADO occurred	$P(\alpha_{ij} \mid g_{ij}, \theta_A)$
1	0/0	Yes	θ_A
2	0/0	No	$1 - \theta_A$
1	0/1	Yes	θ_A
2	0/1	No	$1 - \theta_A$
1	1/1	Yes	θ_A
2	1/1	No	$1 - \theta_A$
1	$1/1'$	Yes	θ_A
2	$1/1'$	No	$1 - \theta_A$
0	0/-	Yes	$\theta_A/2$
1	0/-	No	$1 - \theta_A/2$
0	1/-	Yes	$\theta_A/2$
1	1/-	No	$1 - \theta_A/2$
0	-	No	1
Others			0

Table S2: Definition of the distribution of α_{ij} conditional on g_{ij} and θ_L under LDO mode for DelSIEVE. Definition of the distribution of the number of sequenced alleles (α_{ij}) conditional on the true genotype (g_{ij}) and on the allelic ADO rate (θ_L) under the LDO mode for DelSIEVE.

α_{ij}	g_{ij}	Number of alleles dropped out	$P(\alpha_{ij} \mid g_{ij}, \theta_L)$
0	0/0	2	θ_L^2
1	0/0	1	$2\theta_L(1-\theta_L)$
2	0/0	0	$(1-\theta_L)^2$
0	0/1	2	θ_L^2
1	0/1	1	$2\theta_L(1-\theta_L)$
2	0/1	0	$(1-\theta_L)^2$
0	1/1	2	θ_L^2
1	1/1	1	$2\theta_L(1-\theta_L)$
2	1/1	0	$(1-\theta_L)^2$
0	$1/1'$	2	θ_L^2
1	$1/1'$	1	$2\theta_L(1-\theta_L)$
2	$1/1'$	0	$(1-\theta_L)^2$
0	0/-	1	θ_L
1	0/-	0	$1-\theta_L$
0	1/-	1	θ_L
1	1/-	0	$1-\theta_L$
0	-	0	1
Others			0

Table S3: Evolutionary rate matrix used in the simulator to generate the simulated data for DelSIEVE. Genotypes are encoded with nucleotides rather than numbers. d is the deletion rate measured relatively to the mutation rate. The diagonal elements are denoted by dots, and have negative values equal to the sum of the other entries in the same row, ensuring that the sum of each row equals zero.

	A/A	A/C	A/G	A/T	C/C	C/G	C/T	G/G	G/T	T/T	-A	-C	-G	-T	-
A/A	.	$1/3$	$1/3$	$1/3$	0	0	0	0	0	0	d	0	0	0	0
A/C	$1/6$	.	$1/6$	$1/6$	$1/6$	$1/6$	$1/6$	0	0	0	$d/2$	$d/2$	0	0	0
A/G	$1/6$	$1/6$	.	$1/6$	0	$1/6$	0	$1/6$	$1/6$	0	$d/2$	0	$d/2$	0	0
A/T	$1/6$	$1/6$	$1/6$	.	0	0	$1/6$	0	$1/6$	$1/6$	$d/2$	0	0	$d/2$	0
C/C	0	$1/3$	0	0	.	$1/3$	$1/3$	0	0	0	0	d	0	0	0
C/G	0	$1/6$	$1/6$	0	$1/6$	.	$1/6$	$1/6$	$1/6$	0	0	$d/2$	$d/2$	0	0
C/T	0	$1/6$	0	$1/6$	$1/6$	$1/6$	.	0	$1/6$	$1/6$	0	$d/2$	0	$d/2$	0
G/G	0	0	$1/3$	0	0	$1/3$	0	.	$1/3$	0	0	0	d	0	0
G/T	0	0	$1/6$	$1/6$	0	$1/6$	$1/6$	$1/6$	.	$1/6$	0	0	$d/2$	$d/2$	0
T/T	0	0	0	$1/3$	0	0	$1/3$	0	$1/3$	.	0	0	0	d	0
-A	0	0	0	0	0	0	0	0	0	0	.	$1/6$	$1/6$	$1/6$	$d/2$
-C	0	0	0	0	0	0	0	0	0	0	$1/6$	.	$1/6$	$1/6$	$d/2$
-G	0	0	0	0	0	0	0	0	0	0	$1/6$	$1/6$	.	$1/6$	$d/2$
-T	0	0	0	0	0	0	0	0	0	0	$1/6$	$1/6$	$1/6$	.	$d/2$
-	0	0	0	0	0	0	0	0	0	0	0	0	0	0	.

Table S4: Summary of fractions of predicted genotypes by DelSIEVE and SIEVE for three analyzed real datasets. Entries marked with NA denote that the corresponding method does not call the specific genotype.

		-	1/-	0/-	0/0	0/1	1/1	$1/1'$
TNBC16	DelSIEVE	0	11.51%	0.07%	15.58%	69.82%	2.99%	0.03%
	SIEVE	NA	NA	NA	15.54%	75.11%	9.30%	0.05%
CRC28	DelSIEVE	0	0.15%	0.02%	25.02%	74.59%	0.16%	0.06%
	SIEVE	NA	NA	NA	25.02%	74.64%	0.28%	0.06%
CRC48	DelSIEVE	0	0	0.08%	59.61%	40.17%	0.14%	0
	SIEVE	NA	NA	NA	59.48%	40.50%	0.02%	0
